# Development of [^18^F]AmBF_3_ Tetrazine for Radiolabeling of Peptides: Preclinical Evaluation and PET Imaging of [^18^F]AmBF_3_-PEG_7_-Tyr^3^-Octreotide in an AR42J Pancreatic Carcinoma Model

**DOI:** 10.1021/acs.bioconjchem.2c00231

**Published:** 2022-06-16

**Authors:** Sofia Otaru, Andreas Paulus, Surachet Imlimthan, Iida Kuurne, Helena Virtanen, Heidi Liljenbäck, Tuula Tolvanen, Tatsiana Auchynnikava, Anne Roivainen, Kerttuli Helariutta, Mirkka Sarparanta, Anu J. Airaksinen

**Affiliations:** † Radiochemistry, Department of Chemistry, University of Helsinki, P.O. Box 55, FI-00014 Helsinki, Finland; ‡ Turku PET Centre, University of Turku, Kiinamyllynkatu 4-8, FI-20520 Turku, Finland; § Department of Chemistry, University of Turku, FI-20014 Turku, Finland; ∥ Turku Center for Disease Modeling, Institute of Biomedicine, University of Turku, FI-20520 Turku, Finland; ⊥ Department of Medical Physics, Turku University Hospital, FI-20521 Turku, Finland

## Abstract

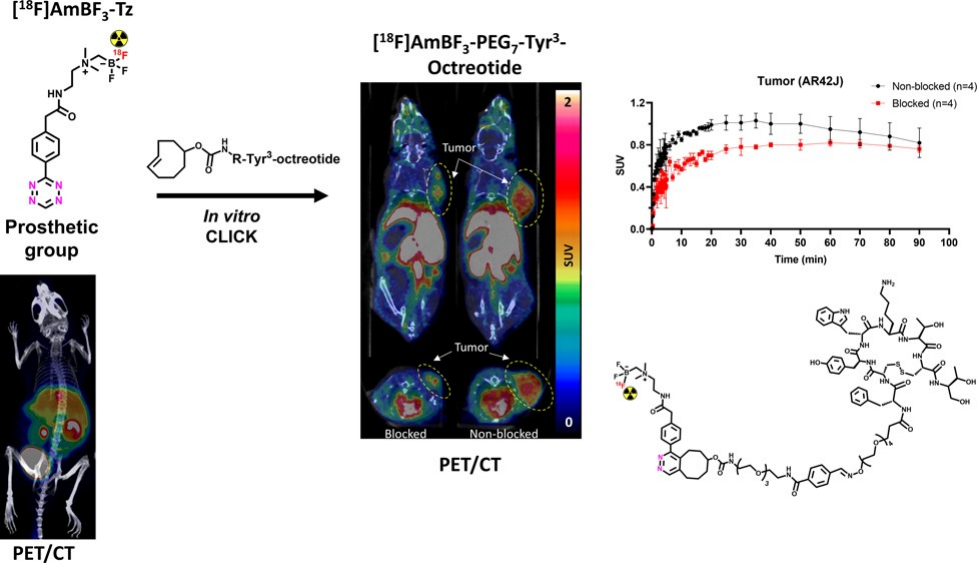

Radiolabeled peptides have emerged as highly specific agents for targeting receptors expressed in tumors for therapeutic and diagnostic purposes. Peptides developed for positron emission tomography (PET) are typically radiolabeled using prosthetic groups or bifunctional chelators for fast “kit-like” incorporation of the radionuclide into the structure. A novel [^18^F]alkylammoniomethyltrifluoroborate ([^18^F]AmBF_3_) tetrazine (Tz), [^18^F]AmBF_3_-Tz, was developed for the [^18^F]fluorination of *trans*-cyclooctene (TCO)-modified biomolecules using Tyr^3^-octreotides (TOCs) as model peptides. [^18^F]AmBF_3_-Tz (A_m_ = 15.4 ± 9.2 GBq/μmol, *n* = 14) was evaluated in healthy mice by *ex vivo* biodistribution and PET/computed tomography (CT), where the radiolabel in the prosthetic group was found stable *in vivo*, indicated by the low bone uptake in tibia (0.4 ± 0.1% ID/g, *t* = 270 min). TCO-TOCs tailored with polyethylene glycol (PEG) linkers were radiolabeled with [^18^F]AmBF_3_-Tz, forming two new tracers, [^18^F]AmBF_3_-PEG_4_-TOC (A_m_ = 2.8 ± 1.8 GBq/μmol, *n* = 3) and [^18^F]AmBF_3_-PEG_7_-TOC (A_m_ of 6.0 ± 3.4 GBq/μmol, *n* = 13), which were evaluated by cell uptake studies and *ex vivo* biodistribution in subcutaneous AR42J rat pancreatic carcinoma tumor-bearing nude mice. The tracer demonstrating superior behavior *ex vivo*, the [^18^F]AmBF_3_-PEG_7_-TOC, was further evaluated with PET/CT, where the tracer provided clear tumor visualization (SUV_baseline_ = 1.01 ± 0.07, *vs* SUV_blocked_ = 0.76 ± 0.04) at 25 min post injection. The novel AmBF_3_-Tz demonstrated that it offers potential as a prosthetic group for rapid radiolabeling of biomolecules in mild conditions using bioorthogonal chemistry.

## Introduction

Biomolecules are increasingly important in nuclear imaging due to their biocompatibility, precise targeting capability, and suitability to various diagnostic and therapeutic applications.^1,2^ Chemical modification of naturally occurring peptides can serve as an avenue toward biologically more stable peptide derivatives, for example, by extending their biological half-life *in vivo*.^3^ Additional functional groups can be included in the peptide structure, enabling chemoselective late-stage bioconjugation reactions.^4^ Due to the ideal physical half-life and imaging properties of the radioisotope (*t*_1/2_ = 109.8 min, positron range in a tissue maximum of 2.4 mm), ^18^F-labeled peptides are desirable alternatives for radiometallated analogues used for clinical somatostatin receptor (SSTR) positron emission tomography (PET) imaging, such as Tyr^3^-octreotate (TATE) and Tyr^3^-octreotide (TOC) derivatives [^68^Ga]Ga-DOTA-TATE and [^68^Ga]Ga-DOTA-1-Nal^3^-octreotide ([^68^Ga]Ga-DOTA-NOC) (^68^Ga, *t*_1/2_ = 68 min, positron range 3.5 mm).^4,5^ However, the direct incorporation of nucleophilic [^18^F]fluoride into a molecule often requires leaving or protecting groups and generally harsher (e.g., alkaline) conditions,^6,7^ limiting its use on structures sensitive to alkalinity or heat, such as proteins.

Mild incorporation of [^18^F]fluoride into biomolecules chemoselectively by isotopic exchange (IE) can be applied instead of the canonical nucleophilic substitution.^8^ However, some of the isotopic exchange reactions, such as the conventional silicon–fluoride (Si–F) exchange, require anhydrous conditions, adding a drying step crucial to the success of the radiolabeling.^9^ When applying the Si–F isotopic exchange to an SSTR2-targeting TATE derivative, a hydrophilic silicon–fluoride acceptor (SiFA)-derivatized [^18^F]F-SiFA*lin*-TATE is developed, and it has successfully entered clinical trials for neuroendocrine tumor (NET) imaging,^10−13^ revealing the true potential of isotopic exchange reactions for clinical radiopharmaceutical development.

Liu et al. developed the radiolabeling of an alkylammoniomethyltrifluoroborate (AmBF_3_)-based prosthetic group, [^18^F]AmBF_3_-alkyne,^14^ utilizing IE radiofluorination that tolerates aqueous conditions, making it well compatible with water-soluble molecules. The method provided [^18^F]AmBF_3_-TATE in one step using IE after click chemistry conjugation of the prosthetic group to the peptide,^15,16^ and the radiosynthesis of [^18^F]AmBF_3_-TATE was successfully modified into a cassette system, yielding up to 10 patient doses in a single run by Lau et al.^17^ Both SSTR targeting tracers, [^18^F]F-SiFA*lin*-TATE and [^18^F]AmBF_3_-TATE, showed favorable pharmacokinetics, high *in vivo* stability, and high image contrast. The AmBF_3_-chemistry has since been utilized for direct IE radiolabeling of various other peptides.^18−22^ As an alternative modular strategy, Iddon et al. reported the development of 2-[^18^F]fluoroethyl azide fluorination reagents suitable for radiolabeling ^18^F-octreotides with reaction times as short as only 5 minutes at room temperature, using copper as a catalyst,^23^ a method specifically useful for sensitive biomolecules. However, compared to other click-based methodologies, the exquisite reaction rate, absence of catalyst, and the biocompatibility of the bioorthogonal inverse electron-demand Diels–Alder (IEDDA) reaction have made it the focal point of click chemistry-based development in biomolecule radiolabeling, especially for pretargeted PET imaging.^24^

Here, leveraging the aqueous compatibility of the AmBF_3_ IE reaction in combination with the unsurpassed kinetics and selectivity of the IEDDA reaction, we report the development of a novel prosthetic group [^18^F]AmBF_3_ tetrazine ([^18^F]AmBF_3_-Tz) suitable for the chemoselective radiolabeling of *trans*-cyclooctene (TCO)-modified biomolecules. As a model system, we radiolabeled two Tyr^3^-octreotides (TOCs), analogues of somatostatin,^25^ in a proof-of-concept study evaluating the influence of the novel prosthetic group on the pharmacokinetics of the well-known peptide analogues *in vivo*.

## Results and Discussion

### Synthesis of AmBF_3_ Tetrazine Precursor (**6**) for Radiolabeling

**Scheme 1:**
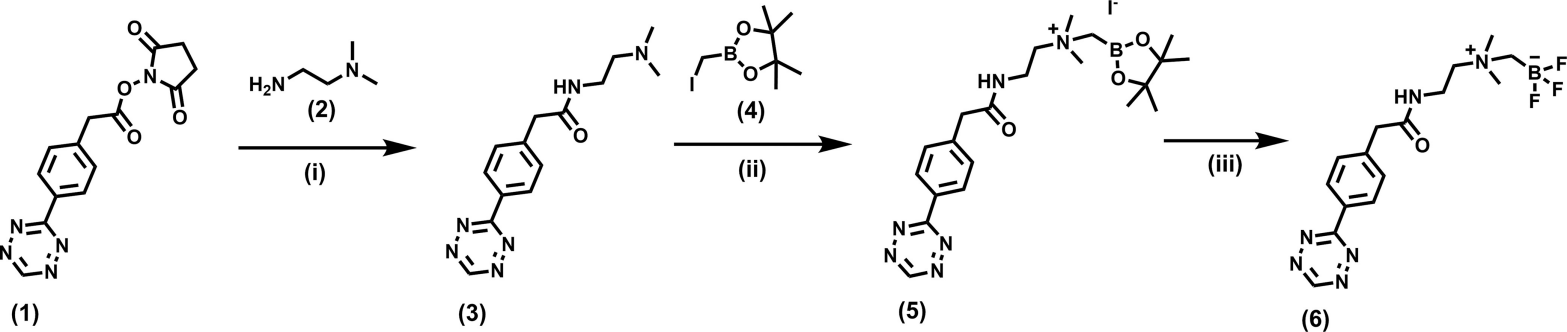
Synthesis of AmBF_3_-Tz (**6**); (i) Dichloromethane, Argon, Ambient Temperature, 1.5 h; (ii) Acetonitrile, Argon, Ambient Temperature, Overnight; and (iii) 3 M KHF_2_, 4 M HCl, Water, Dimethylformamide (DMF), 30 min at 70 °C

The synthesis of the AmBF_3_ tetrazine was designed in a stepwise manner to incorporate the boronic acid pinacol ester selectively into the tertiary amine, followed by acid-catalyzed fluorination of the pinacol ester to afford the trifluoroborate. During the synthesis, it was crucial to take into account the susceptibility of the tetrazine, a redox mediator,^26^ to readily reduce into “unreactive” dihydrotetrazine in the presence of a reducing agent, especially when heated. The synthesis of the trifluoroborate required anhydrous conditions for the nucleophilic substitution of the haloalkane in the pinacol ester, and the subsequent fluorination step required a corrosive-resistant reaction vessel, careful handling, and good ventilation due to the formation of corrosive and toxic HF (g), even if in small quantities. AmBF_3_-Tz (**6**) was synthesized with an overall yield of ∼36% (Scheme 1). The nuclear magnetic resonance (NMR) spectroscopy analysis revealed in the ^1^H NMR a characteristic signal at the para-position of the Tz ring at 10 ppm (Supporting Figure S1), and the presence of the Tz ring was verified by high-performance liquid chromatography coupled to a diode-array detector (HPLC–DAD) at 534 nm, by the characteristic absorbance wavelength for Tz (>500 nm) (Supporting Figure S2). ^19^F NMR spectra of **6** displayed splitting of the signal due to coupling to the trifluoroborate boron, and the ^11^B NMR spectra likewise revealed the boron-11 coupling to fluorine-19, detected as a split quartet signal (Supporting Figures S3–S5 for ^11^B, ^19^F and ^13^C NMR). Compound **6** eluted at *t*_R_ = 4.59 min, when analyzed by ultrahigh-performance liquid chromatography high-resolution mass spectrometry (UHPLC-HRMS), with a detected molecular ion peak corresponding to the protonated [M + H]^+^ ion (Supporting Figure S6).

### Modification of Tyr^3^-Octreotide-PEG_4_-ONH_2_ with *trans*-Cyclooctene and IEDDA Cycloaddition

**Figure 1:**
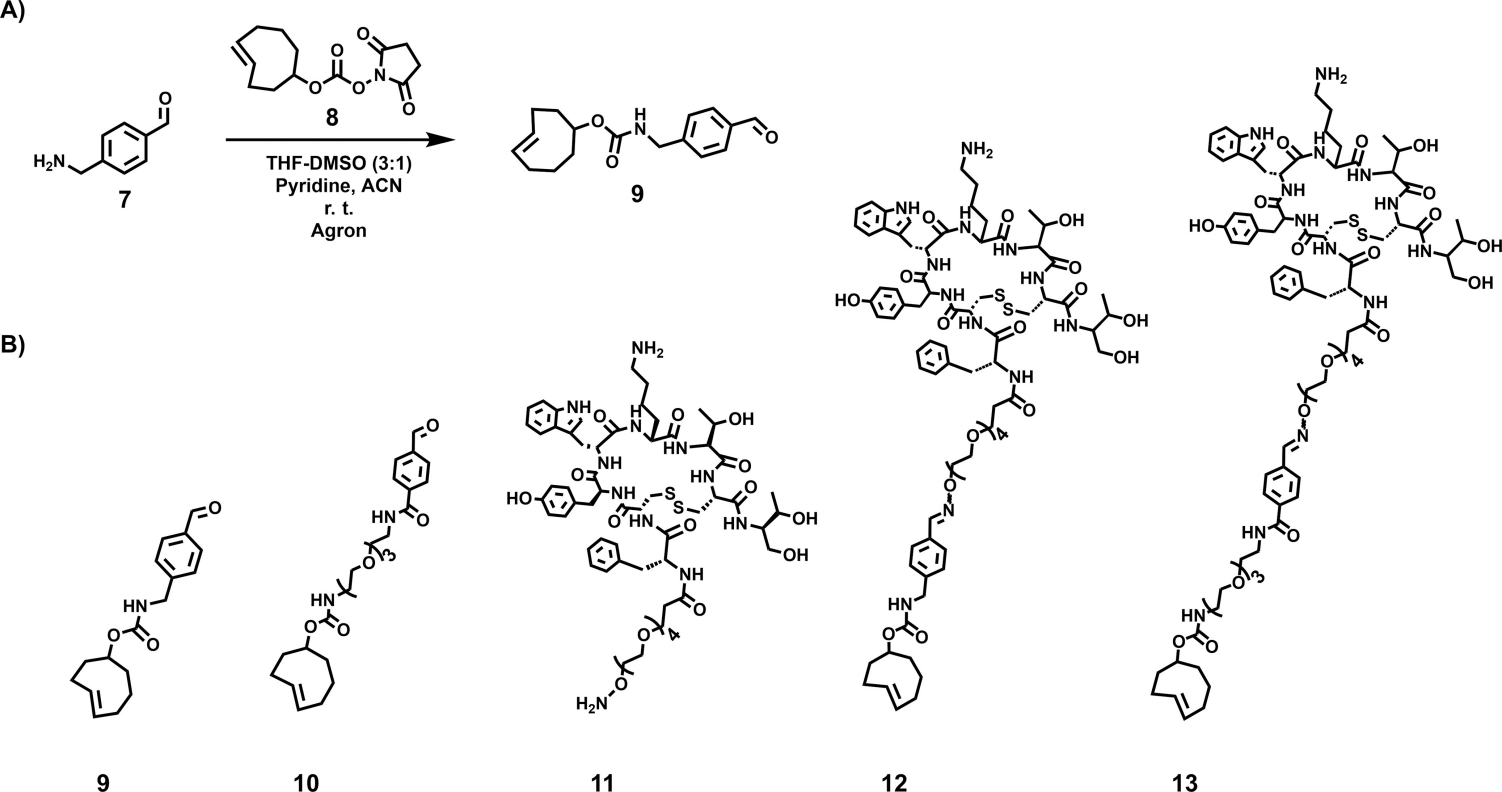
(A) Synthesis of TCO-CHO (**9**). (B) Chemical structures of TCO compounds: TCO-CHO (**9**), TCO-PEG_3_-CHO (**10**), TOC-PEG_4_-ONH_2_ (**11**), TCO-PEG_4_-TOC (**12**), and TCO-PEG_7_-TOC (**13**).

TCO-CHO (**9**) was synthesized at 21 ± 5% (*n* = 3) yield in one step, characterized by NMR and HPLC (Figures 1A and S7–S9 for ^1^H and ^13^C NMR and HPLC chromatogram). TCO-aldehydes **9** (synthesized in-house) and **10** (commercially available) were conjugated to TOC (**11**, custom-synthesized, purchased from CSBio, Menlo Park, CA, USA, Figure 1B). TCO-TOCs **12** and **13** (TCO-modified in-house, purity ≥ 99%) were purified with HPLC (see the Supporting HPLC method A). Compound **13** eluted at *t*_R_ = 5.22 min as two protonated molecule ions: [M + 3H]^3+^ at 586.61316 *m*/*z* with Δ = −2.35545 ppm (calculated 586.61454 *m*/*z* for C_85_H_125_O_23_N_13_S_2_^3+^) and [M + 2H]^2+^ at 879.41663 *m*/*z* with Δ = −1.75922 ppm (calculated 879.41817 *m*/*z* for C_85_H_124_O_23_N_13_S_2_^2+^) when analyzed with UHPLC-HRMS. Compound **12** eluted at *t*_R_ = 12.8 min on liquid chromatography mass spectrometry (LC-MS) and was found as a protonated molecule ion corresponding to protonated [M + 2H]^2+^ (found *m*/*z* 784.7, calculated *m*/*z* 784.4 for C_77_H_108_N_12_O_19_S_2_^2+^). After purification, **6** was incubated with **12** or **13** in an aqueous solution for conjugating the TCO-peptides with **6** as reference compounds **14** and **15** by IEDDA. Products **14** and **15** were analyzed with LC-MS and UHPLC-HRMS, respectively. The NMR, HPLC, liquid chromatography mass spectrometry (LC-MS), and UHPLC-HRMS data for the synthesized compounds are presented in the Supporting Information (Supporting Figures S1–S14).

### [^18^F]Fluorination of **6**

**Figure 2:**
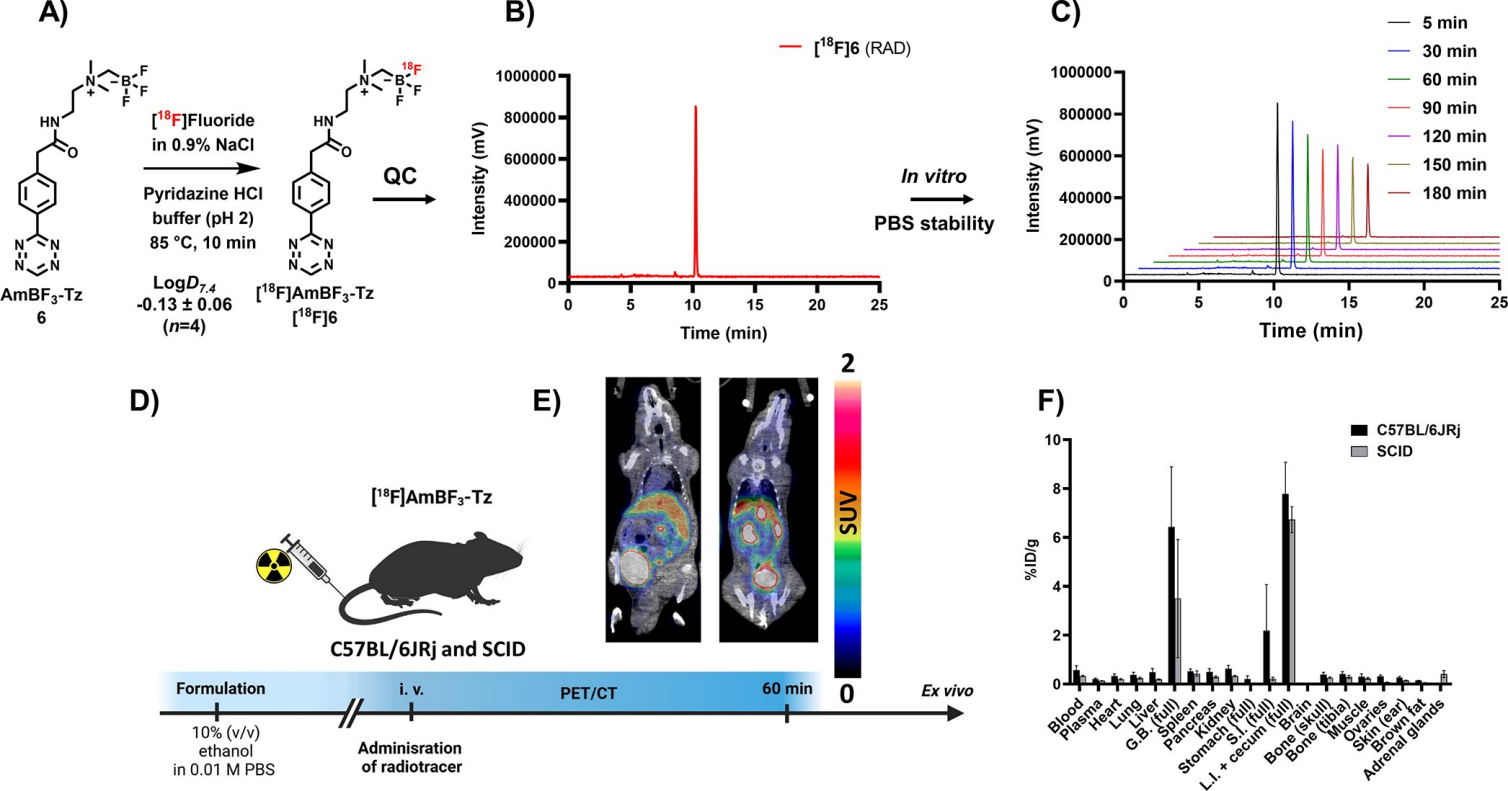
Summary of *in vitro*, *ex vivo*, and *in vivo* evaluations of **[**^**18**^**F]6**. (A) Radiolabeling conditions and log D_7.4_ of **[**^**18**^**F]6**. (B) Quality control (QC) of **[**^**18**^**F]6** (radio-HPLC). (C) Hydrolytic stability of **[**^**18**^**F]6** in 0.01 M phosphate-buffered saline (PBS). (D) Graphic depiction of PET/CT and *ex vivo* study of **[**^**18**^**F]6**. (**E)** PET/CT image of **[**^**18**^**F]6** in male severe combined immunodeficient (SCID) mouse (left panel) and healthy female C57BL/6JRj mouse (right panel) at 60 min post injection. (F) *Ex vivo* biodistribution of **[**^**18**^**F]6** after PET/CT imaging (*t* = 270 min) in SCID (male) and C57BL/6JRj (female) mice. (G.B., gallbladder; S.I., small intestines; L.I., large intestines). The data points present the mean ± standard deviation of the % ID/g values.

Prosthetic group **6** was radiolabeled with a protocol partly based on a methodology developed by Liu et al.^14^ The radiosynthesis of **[^18^F]6** is presented in Figure 2. Modifications to the [^18^F]fluoride eluent and radiolabeling buffer were done to alter the conditions more suitable for our prosthetic group and setup, ensuring repeatable radiolabeling yields (20.8 ± 10.3%, *n* = 7) in microliter volumes. The optimal reaction volume in our conditions was a mere 10–20 μL. Decreasing the volume by 2.5 times increased the yield by 6 times at 85 °C (0.9% NaCl elution, 200 nmol of **6**, Supporting Figure S15), and the radiochemical yield (RCY) decreased dramatically if the reaction mixture was evaporated to dryness or when the final volume exceeded 20 μL. However, for elution of reasonable amounts of [^18^F]fluoride out of the PS-HCO_3_ (Macherey-Nagel, Düren, Germany) solid-phase extraction (SPE) ion exchange cartridge, a minimum 20–30 μL of 0.9% NaCl was required. Therefore, we chose to substitute the commonly used aqueous 0.9% NaCl as the [^18^F]fluoride eluent altogether and opted for a pyridazine HCl eluent formulation, similarly as reported by Kwon et al.^27^ The pyridazine HCl buffer recipe was modified to best serve our setup, as a combination of pyridazine (9 v/v%)–acetonitrile (61 v/v%)–DMF (13 v/v%)–H_2_O (13 v/v%)–12 M HCl (4 v/v%), and the pH was adjusted to 2. With the modified buffer, the [^18^F]fluoride release efficiency from the cartridge remained high (93 ± 2%, *n* = 3), providing a suitable reaction medium for radiolabeling directly after rapid concentration (∼10 min), achieved by decreasing the evaporation time from 45 min (100 μL of 0.9% NaCl as the eluent) to 10 min (100 μL of the modified pyridazine HCl buffer, pH 2.0) in our setup . The evaporation time was further cut in half by adding more DMF to the buffer (water quantity from ∼38 to ∼12% *v/v*), which made the control of the final volume easier, and improved the RCY (**[**^**18**^**F]6**; 8–37% DCY), which reached the range of previously published [^18^F]AmBF_3_ tracers (∼16–35%).^14,18,19,22^**[**^**18**^**F]6** was obtained with molar activity (A_m_) of 6–39.8 GBq/μmol from the concentrated [^18^F]fluoride in 15 min at 85 °C. The radiochemical yield (RCY) and radiochemical purity (RCP) for **[**^**18**^**F]6** were 20.8 ± 10.3% (*n* = 7, DCY) and ≥98%, respectively (Figures 2B for radio-HPLC and S16 for radio-TLC). Typically, 0.2–2.1 GBq of [^18^F]AmBF_3_-Tz with molar activity of 15.4 ± 9.2 GBq/μmol was obtained starting with 2–12 GBq of [^18^F]fluoride.

### Radiolabeling of Tyr^3^-Octreotide Analogues **14** and **15**

**Figure 3:**
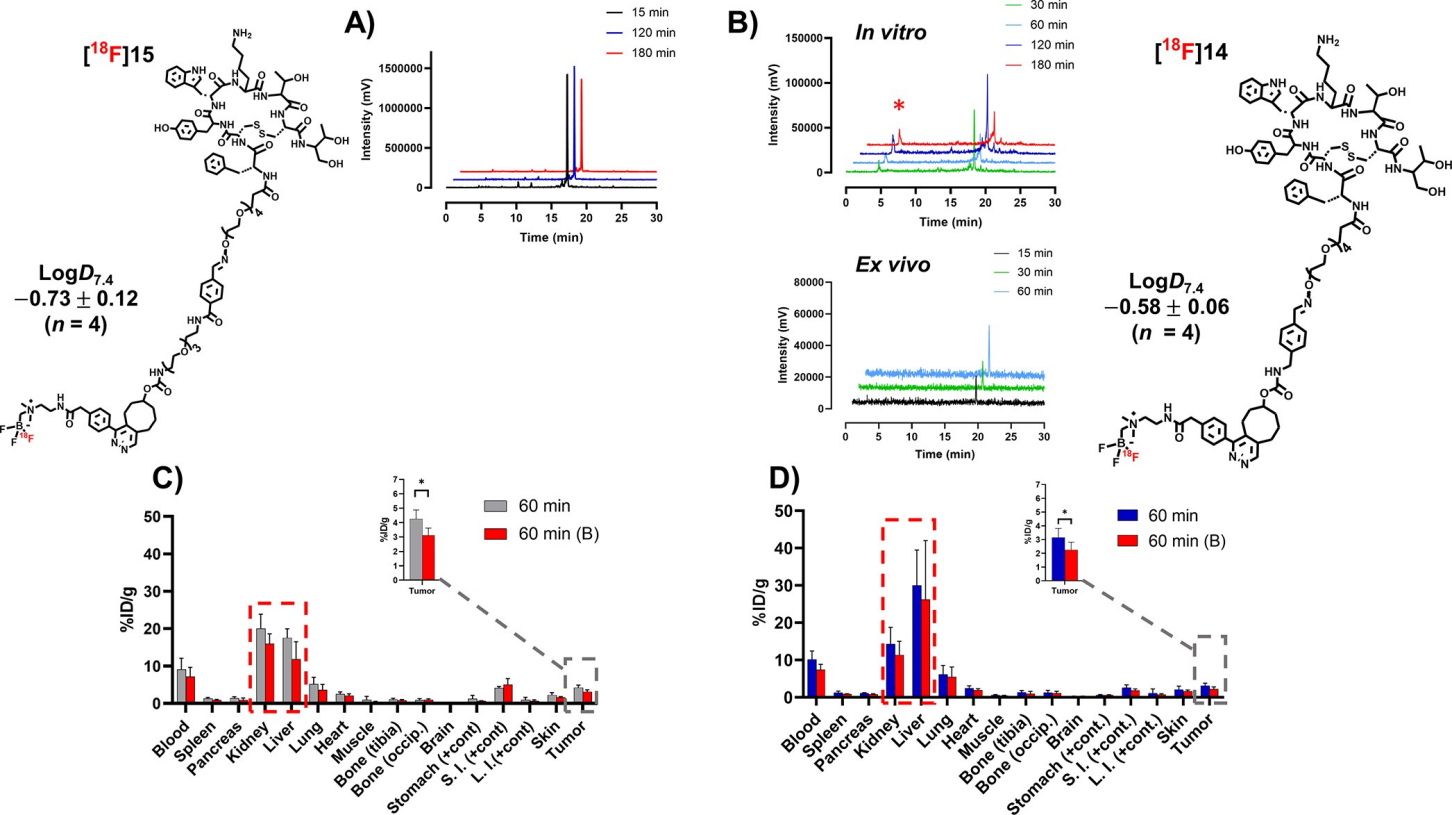
Chemical structures, together with log D_7.4_ values, and a summary of *in vitro* and *ex vivo* evaluations of **[**^**18**^**F]14** and **[**^**18**^**F]15**. Enzymatic stability *in vitro* in 50% human plasma in 0.01 M PBS (pH 7.4) of (A) **[**^**18**^**F]15** (radio-HPLC) and (B) **[**^**18**^**F]14** together with *ex vivo* mouse blood stability of **[**^**18**^**F]14** (radio-HPLC). Red asterisk (red star) denotes a detected radiometabolite of **[**^**18**^**F]14** in the radiochromatogram in the *in vitro* enzymatic stability sample. *Ex vivo* distribution of radioactivity after intravenous administration of (C) **[**^**18**^**F]15** and (D) **[**^**18**^**F]14** (occip., occipital; cont., content; S.I., small intestines; L.I., large intestines). In graphs (C, D), the values are presented as mean ± standard deviation.

**Scheme 2:**
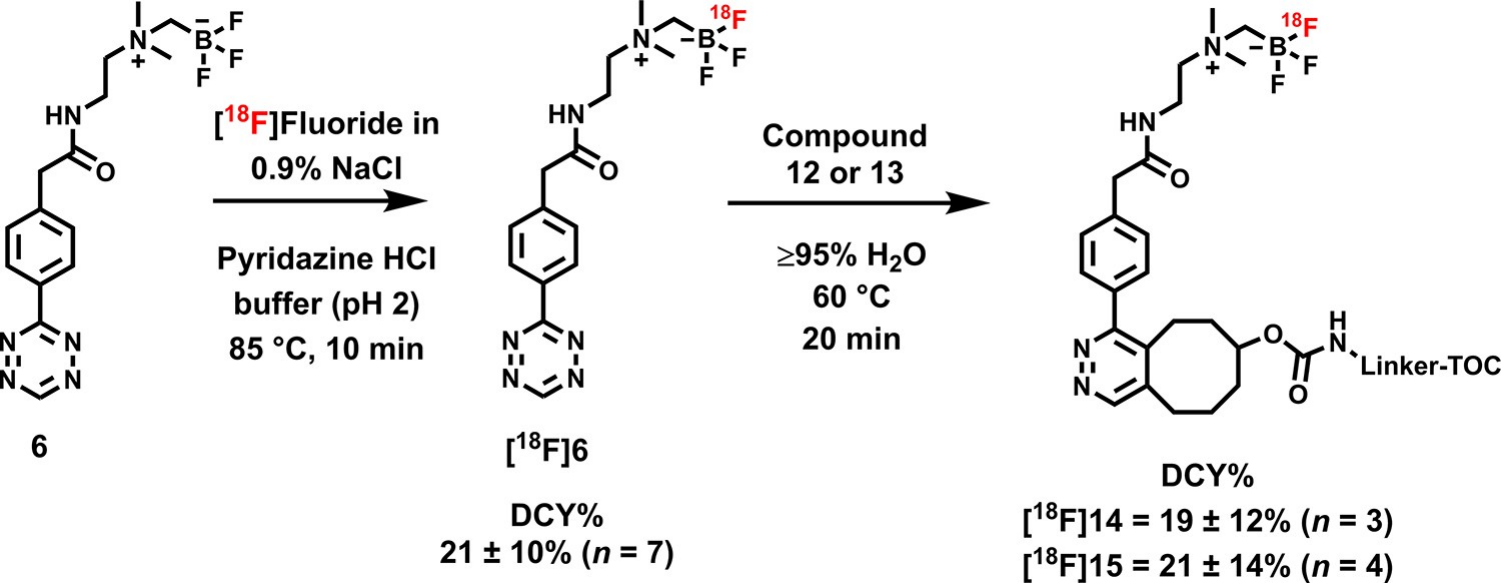
Radiosyntheses of [^18^F]F-TOCs **[**^**18**^**F]14** and **[**^**18**^**F]15**

*Trans*-cyclooctene-modified TOCs **12** and **13** were radiolabeled with **[**^**18**^**F]6** providing **[**^**18**^**F]14** and **[**^**18**^**F]15** (Scheme 2 and Figure 3). The total synthesis time was in an average of 85–102 min (Table 1). The radiochemical yields for **[**^**18**^**F]14** and **[**^**18**^**F]15** starting from the prosthetic group **[**^**18**^**F]6** ranged from 8 to 34%. The decay-corrected RCYs of the radiolabeled TOCs, comprising the production of **[**^**18**^**F]6** and of the subsequent IEDDA reaction (two steps), starting from [^18^F]fluoride, ranged between approximately 2 and 8%, with the radioactivity obtained at 53–130 MBq for **[**^**18**^**F]14** and 78–267 MBq for **[**^**18**^**F]15**, with RCPs of ≥ 99% (Supporting Figures S17 and S18), and molar activity range of 1.0–9.4 GBq/μmol. The RCYs were low, partly due to the compromise of using the prosthetic group **[**^**18**^**F]6** (100 nmol) in a molar excess of minimum 2:1 to the TOC precursor **14** or **15** (50 nmol) during IEDDA in order to consume the TCO-modified peptide completely to avoid having unlabeled TOC–TOC as a competitor in the final formulation. **[**^**18**^**F]6**, **[**^**18**^**F]14**, and **[**^**18**^**F]15** required only a SPE cartridge purification prior to administration, rendering the method suitable for a cassette-based radiolabeling system, similar to that reported by Allott et al.^28^ The loss of radioactivity could be decreased by altering the ratio of the TCO biomolecule to the radiolabeled tetrazine during IEDDA, but the biggest loss of radioactivity was attributed to [^18^F]fluoride escaping likely as [^18^F]HF in the acidic conditions already during the concentration step. This could be hypothetically resolved by employing microfluidic trapping in lieu of heat-induced evaporation for the [^18^F]fluoride concentration. The synthesis times for **[**^**18**^**F]14** and **[**^**18**^**F]15** were relatively long (85–102 min) when compared to the 60 minute synthesis time with the Trasis AllinOne module reported by Lau et al.^17^ and to the 25 min reported by Liu et al.,^29^ both for [^18^F]AmBF_3_-TATE. In the aforementioned studies, the molar activities of [^18^F]AmBF_3_-TATE (Lau et al., 435 ± 162 GBq/μmol; Liu et al., >111 GBq/μmol) were considerably higher than those in our study (**[**^**18**^**F]14** = 2.8 ± 1.8 GBq/μmol; **[**^**18**^**F]15** = 6.0 ± 3.4 GBq/μmol), likely as a result of the stepwise radiosynthesis of **[**^**18**^**F]14** and **[**^**18**^**F]15** (Scheme 2), resulting in a loss of radioactivity in each step, circumvented in the one-step radiofluorination of [^18^F]AmBF_3_-TATE. Furthermore, the molar ratio of **[**^**18**^**F]6** to the TCO-peptide **12** or **13** was kept at least at 2:1, resulting in anticipated loss of radioactivity during the IEDDA.

**Table 1:** Radiolabeling Results of TOC Tracers **[**^**18**^**F]14** and **[**^**18**^**F]15^a^**

	[^18^F]14	[^18^F]15
synthesis time (min)	85 ± 8 (*n* = 3)	102 ± 29 (*n* = 17)
RCY (%) from **[**^**18**^**F]6**	19.3 ± 11.6 (*n* = 3)	21.4 ± 13.5 (*n* = 4)
overall RCY (%) from [^18^F]fluoride	3.3 ± 1.7 (*n* = 3)	5.1 ± 3.4 (*n* = 5)
RCP (%)	≥99	≥99
A_m_ (GBq/μmol)	2.8 ± 1.8 (*n* = 3)	6.0 ± 3.4 (*n* = 13)

a Yields are decay-corrected to the start of synthesis.

### *In Vitro* Stability and Lipophilicity

**Figure 4:**
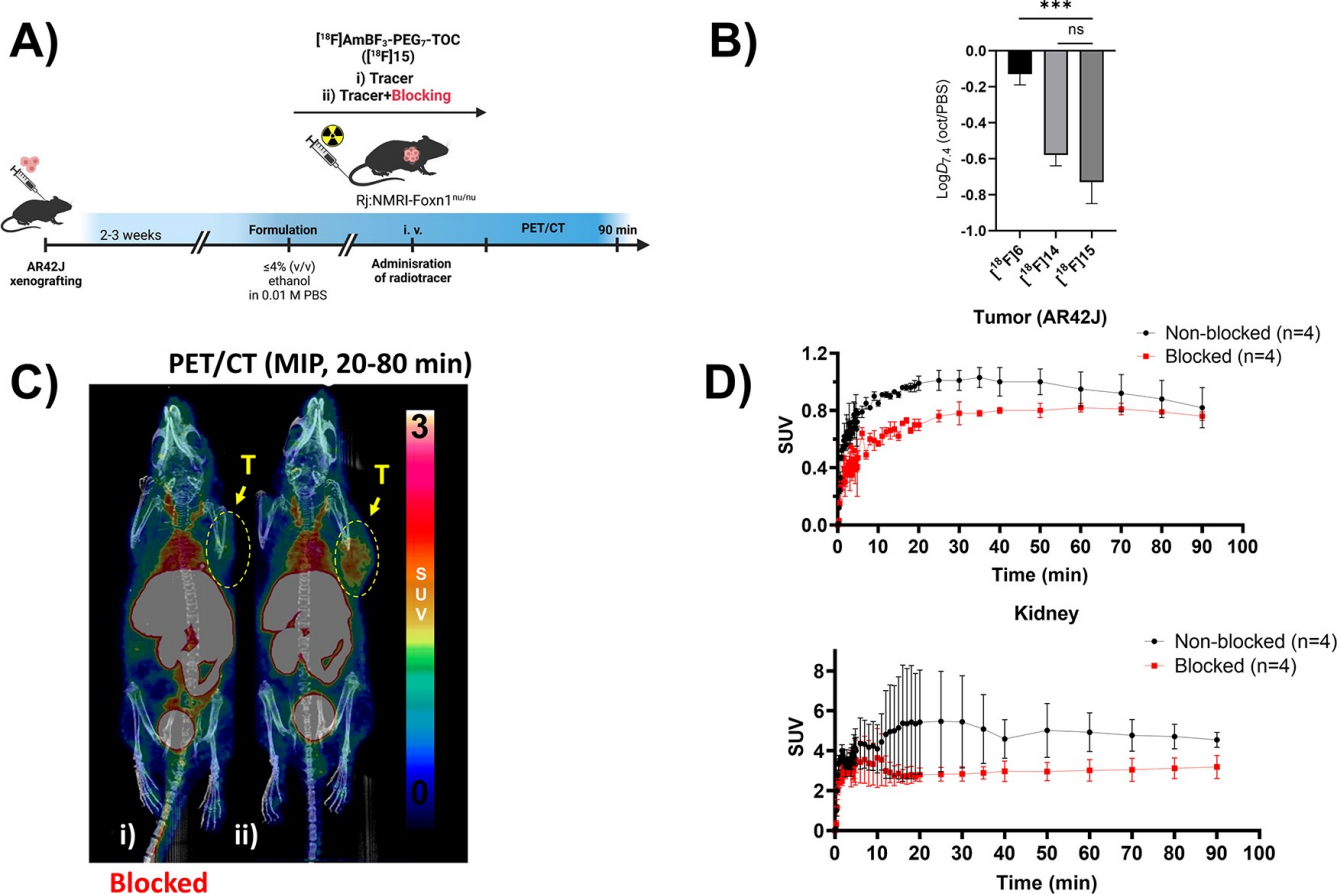
Summary of *in vivo* evaluation of **[**^**18**^**F]15**, together with lipophilicities of **[**^**18**^**F]6**, **[**^**18**^**F]14**, and **[**^**18**^**F]15**. General depiction of the study design from implantation of tumor cells (AR42J) to PET/CT imaging of mice (A). Measured log D_7.4_ values of **[**^**18**^**F]6**, **[**^**18**^**F]14**, and **[**^**18**^**F]15** (B). Maximum intensity projection (MIP) PET/CT images from the selected time-window (*t* = 20–80 min) post injection of **[**^**18**^**F]15** in AR42J tumor-bearing nude mice in blocking (i) and baseline (ii) conditions (C). Tumor uptake and the elimination of radioactivity into kidneys as a function of time during the PET/CT imaging presented as time–activity curves (TACs) plotted from standardized uptake values (SUVs) (D). Data points are expressed as mean ± standard deviation.

**[**^**18**^**F]6** demonstrated favorably low lipophilicity (log D_7.4_ = −0.13 ± 0.06, *n* = 4) and good hydrolytic stability (≥ 99% intact at *t* = 3 h, 0.01 M PBS, pH 7.4) (Figure 2C). Log D_7.4_ values for **[**^**18**^**F]14** and **[**^**18**^**F]15** were −0.58 ± 0.06 and −0.73 ± 0.12 (*n* = 4), respectively, both demonstrating a lower lipophilicity than the prosthetic group alone and a decrease in log D_7.4_ with increasing PEG chain length, as expected (Figure 4B). **[**^**18**^**F]15** had a higher lipophilicity (−0.7 ± 0.1, *n* = 4) than that reported for [^18^F]F-SiFA*lin*-TATE (−1.2 ± 0.1), which likely results from the IEDDA cycloaddition product. **[**^**18**^**F]14** and **[**^**18**^**F]15** were found stable in the formulated solution, 4% ethanol–0.01 M PBS (pH 7.4), when sampled at 9 h and at several time points up to 6 h, respectively (Supporting Figures S19 and S20). The enzymatic stability assay in 50% (v/v) human plasma–0.01 M PBS revealed that **[**^**18**^**F]15** was stable up to at least 180 min (Figure 3A). **[**^**18**^**F]14**, on the other hand, demonstrated a lower enzymatic stability than expected, and a polar radiometabolite was detected in the HPLC chromatogram during the *in vitro* plasma stability study of **[**^**18**^**F]14** (Figure 3B).

### Cell Uptake Studies

The cell uptake of **[**^**18**^**F]14** and **[**^**18**^**F]15** was studied in SSTR2-expressing rat pancreatic adenocarcinoma AR42J cells, where **[**^**18**^**F]14** showed a significant difference (*p* < 0.05) in the cell uptake in baseline versus blocking conditions (baseline = 1.0 ± 0.2% at 120 min, *n* = 3, vs blocking = 0.5 ± 0.1% at 120 min, *n* = 3, *p* = 0.001) from 60 min onward (Supporting Figure S21). **[**^**18**^**F]15** demonstrated an overall higher cell uptake *in vitro*, which was effectively blocked by an excess of native TOC (baseline = 6.1 ± 0.6% at 120 min, *n* = 3, vs blocking = 0.7 ± 0.1% at 120 min, *n* = 3, *p* < 0.005, Supporting Figure S22), corroborating that the uptake was specific and receptor-mediated.

### PET/CT and *Ex Vivo* Biodistribution of **[**^**18**^**F]6**

The prosthetic group **[**^**18**^**F]6** was studied as a standalone tracer for evaluating the stability of its radiolabel (B-^18^F) *in vivo*. Moreover, **[**^**18**^**F]6** was hypothesized to have beneficial properties, if stable *in vivo*, as a pretargeting tool. **[**^**18**^**F]6** in 10% (v/v) ethanol–0.01 M PBS, 11 nmol, 150 μL, was administered intravenously to male SCID (11.0 ± 0.5 MBq) and female C57BL/6JRj (11.3 ± 0.3 MBq) mice (*n* = 4 per strain) (Figure 2D). Five minutes post injection, **[**^**18**^**F]6** demonstrated low uptake in major organs and fast clearance from the blood, as illustrated by the time–activity curve (TAC) for the heart (left ventricle, Supporting Figure S23). An elevated liver uptake, possibly due to the tetrazine moiety, which decreased steadily throughout the 50 min dynamic image acquisition, was also visible. The elimination of radioactivity from the tissues during the PET/CT image acquisition, presented as TACs, indicated that the prosthetic group eliminates quickly, mainly through the kidneys (Supporting Figure S23). PET/CT was followed by *ex vivo* biodistribution 270 min post injection, which confirmed the optimal pharmacokinetics and high *in vivo* stability of the radiolabel in **[**^**18**^**F]6**, indicated by the fast clearance of radioactivity from the major organs and the low bone uptake in the tibia (0.4 ± 0.1% ID/g for C57BL/6JRj, 0.3 ± 0.1% ID/g for SCID; Figure 2F). Pronounced elimination into the gallbladder (6.4 ± 2.5% ID/g for C57BL/6JRj, 3.5 ± 2.4% ID/g for SCID) was also seen, but the major elimination pathway was renal clearance. The radiolabel stability and the beneficial pharmacokinetic characteristics of **[**^**18**^**F]6** prompted its use for peptide radiolabeling and revealed its potential as a pretargeting radiotracer, currently under investigation by our group.

### *Ex Vivo* Biodistribution of **[**^**18**^**F]14** and **[**^**18**^**F]15**

After intravenous administration *ex vivo*, the tumor uptake of **[**^**18**^**F]14** and **[**^**18**^**F]15** in AR42J xenografts was partly blocked by octreotide (**[**^**18**^**F]14** baseline = 3.1 ± 0.7, vs blocked = 2.2 ± 0.6, *p =* 0.0120; and **[**^**18**^**F]15** baseline = 4.5 ± 1.0 vs blocked = 3.1 ± 0.5, *p* = 0.0143). In comparison to the other SSTR2-targeting radiotracers, **[**^**18**^**F]14** and **[**^**18**^**F]15** demonstrated tumor uptakes in the range of other reported TOCs and TATEs with similar molar activities (^18^F-FETE-PEG-TOCA, A_m_ = 5.9 GBq/μmol and 5.14% ID/g in tumor; A_m_ = 3.9 GBq/μmol and 8.23% ID/g in tumor; ^18^F-FETβAG[W-*c*-K] A_m_ = 12.3 GBq/μmol and 0.10% ID/^18^F-FET-βAG-TOCA in tumors) in AR42J tumor-bearing mice,^30^ likely arising from the moderate molar activities in this study (Table 2). The bulky molecular size of the cycloadducts in **[**^**18**^**F]14** and **[**^**18**^**F]15**, including the linker as well as the structural modifications might also have led to alteration of the performance and the somewhat inferior pharmacokinetics. The prolonged blood pool retention made the radiotracers readily available for an extended period of time, enabling the increase of nonspecific tracer accumulation in the tumor. Higher uptakes in AR42J tumors were obtained for both [^18^F]AmBF_3_-TATE (10.1 ± 1.7% ID/g) and [^18^F]F-SiFA*lin*-TATE (18.5 ± 4.9% ID/g), with significantly higher molar activities of >111 and 44–63 GBq/μmol, respectively.^13,29^ The uptake of radioactivity after administration of **[**^**18**^**F]14** and **[**^**18**^**F]15** in the pancreas was lower in comparison to the uptake of [^18^F]AmBF_3_-TATE in the pancreas published by Lau et al. (**[**^**18**^**F]14**: baseline = 1.1 ± 0.2% ID/g, blocked = 0.8 ± 0.5% ID/g, *p* = 0.0087 vs **[**^**18**^**F]15**: baseline = 1.6 ± 0.5% ID/g, blocked = 0.9 ± 0.5% ID/g, *p* = 0.1655 ns. vs [^18^F]AmBF_3_-TATE: baseline 14.3 ± 1.6% ID/g, blocked = 0.2 ± 0.1% ID/g).^17^ This apparent nonspecificity likely also arises from high RBC binding of the tracer **[**^**18**^**F]14** that cannot be blocked in the organs with a large blood pool, together with the low molar activity of the tracer, which might be increased by preconjugation of **6** to the TOC analogues prior to radiolabeling. However, the pancreatic uptake was of similar magnitude reported earlier for [^18^F]AmBF_3_-TATE by Liu et al. (pancreas; baseline = 2.8 ± 1.5% ID/g, blocked = 0.2 ± 0.1% ID/g).^29^ Furthermore, the obtained molar activities in this study influenced the receptor uptake and the blocking efficiency of the radiotracers, which is challenging to address when using isotopic exchange as the radiolabeling strategy. The elimination was predominantly by renal clearance at 60 min post injection (**[**^**18**^**F]14**: baseline ∼85% ID/g; **[**^**18**^**F]15:** baseline ∼174% ID/g), accompanied by a high accumulation into the gallbladder for both tracers **[**^**18**^**F]14** (baseline = 17.8 ± 5.2% ID/g, blocked = 10.7 ± 6.3% ID/g) and **[**^**18**^**F]15** (baseline = 9.1 ± 7.0% ID/g, blocked = 18.8 ± 10.3% ID/g), a phenomenon typically present when IEDDA is used as the radiolabeling strategy. Based on the pronounced renal clearance, **[**^**18**^**F]15** resembled [^18^F]AmBF_3_-TATE and [^18^F]F-SiFA*lin*-TATE and would likely provide lower kidney reabsorption rates than the radiometallated SSTR targeting peptides currently in clinical use. The higher accumulation of radioactivity in the abdominal region with **[**^**18**^**F]15**, which can be partly attributed to the PEG chain prolonging residence in circulation, will likely result in lower tumor-to-background ratios than those reported for [^18^F]AmBF_3_-TATE and [^18^F]F-SiFA*lin*-TATE.^29,31^ The good hydrolytic stability, revealed by the low bone uptake of the tracers **[**^**18**^**F]14** and **[**^**18**^**F]15** at 60 min post injection, is at an equal level as for the previously published [^18^F]AmBF_3_-TATE (femur = 1.5–1.7% ID/g at 30 min) by Lau et al.^17^ (**[**^**18**^**F]14**: tibia, baseline = 1.3 ± 0.6% ID/g, blocked = 1.0 ± 0.6% ID/g, vs **[**^**18**^**F]15**: tibia, baseline = 1.1 ± 0.4% ID/g, blocked = 0.8 ± 0.2% ID/g). Notably, tracers **[**^**18**^**F]14** and **[**^**18**^**F]15** were sampled at a later time point than [^18^F]AmBF_3_-TATE,^17^ indicative of at least comparable stability of the radiotracers *in vivo*. Interestingly, the radioactivity in bone increased from 60 to 120 min post injection only for **[**^**18**^**F]14** (tibia: 2.9 ± 1.4% ID/g; occipital: 1.7 ± 0.1% ID/g) but not for **[**^**18**^**F]15** (tibia: 0.6 ± 0.4% ID/g; occipital: occipital 0.6 ± 0.1% ID/g). The *ex vivo* radiometabolite analysis by radio-TLC indicated that **[**^**18**^**F]14** was metabolized and two radiometabolites were detected in blood at 5 and 30 min post injection (radio-TLC; Supporting Figure S26), in accordance with the *in vitro* enzymatic stability assay results (Figure 3B). A sample taken at 60 min post-injection revealed the same polar metabolite in blood, while in urine a less-retained, less-polar metabolite in trace amounts was seen, leaving approximately 99% of the radiotracer intact in both urine and blood. The prolonged blood residence of the TOC derivatives persisting at 60 min warrants further evaluation. After administration of **[**^**18**^**F]14**, blood samples were taken, and the radioactivity in separated blood components was analyzed. The free fraction of the tracer was 72.9 ± 5.1% at 5 min and remained high until 60 min post injection (68.5 ± 5.3%). This indicates that the tracer was readily available at a steady rate throughout the study. Radioactivities of 22 and 25%, respectively, at 5 and 60 min, were bound to red blood cells (RBCs) (Supporting Table S1). In blocking conditions at 60 min, the free fraction seemed to decrease (55.7 ± 11.4%), and the RBC-bound fraction grew (29.7 ± 2.9%). The binding to RBCs slightly grew from 5 to 60 min post injection. This could have contributed to the long circulation time and high background radioactivity levels in organs with a large blood reservoir, such as the liver, and a slight rise in bone uptake detected for both **[**^**18**^**F]14** and **[**^**18**^**F]15** at 60 min in the tibia containing the bone marrow. A minor degree of defluorination could not be ruled out for the compound **[**^**18**^**F]14**, but with **[**^**18**^**F]15**, there was no indication of defluorination. Based on the overall superior performance over **[**^**18**^**F]14**, tracer **[**^**18**^**F]15** was chosen as the lead compound for further evaluation with PET/CT.

**Table 2:** A_m_ and *Ex Vivo* Results for TOC Tracers **[**^**18**^**F]14** and **[**^**18**^**F]15** of Selected Organs at 60 min Post Injection^a^

	[^18^F]14	[^18^F]15
tracer A_m_ (GBq/μmol)	2.8 ± 1.8	6.0 ± 3.4
tumor (baseline, % ID/g)	3.1 ± 0.7	4.5 ± 1.0
tumor (blocked, % ID/g)	2.2 ± 0.6	3.1 ± 0.5
T/blood ratio (baseline)	0.30	0.5
T/blood ratio (blocked)	0.30	0.4
urine (baseline, % ID/g)	84.9 ± 58.6	174.2 ± 73.4
urine (blocked, % ID/g)	41.9 ± 15.7	303.4 ± 145.8
liver (baseline, % ID/g)	30.0 ± 9.4	19.0 ± 5.4
bone,tibia(baseline, % ID/g)	1.3 ± 0.6	1.1 ± 0.4

a The numerical values represent the mean ± standard deviation of the % ID/g values. *n* ≥ 3.

### PET/CT Imaging of **[**^**18**^**F]15**

Based on the higher tumor uptake, more efficient blocking, better stability, and superior pharmacokinetics *ex vivo*, peptide **[**^**18**^**F]15** was selected over **[**^**18**^**F]14** for further evaluation by PET/CT imaging. After intravenous administration of **[**^**18**^**F]15** (0.2 nmol), the radioactivity in the subcutaneous AR42J tumor increased slowly and peaked at 20–30 min, as demonstrated by the TACs (Figure 4D). The tumor was well visualized, as seen in the maximum intensity projection (MIP) PET/CT image (Figure 4C,i). The tumor uptake was partly blocked (Figure 4C,ii) with the coadministration of octreotide (45 μg, 44 nmol). The maximum intensity projection (MIP) images of **[**^**18**^**F]15** at 20–80 min post injection in AR42J tumor-bearing mice (Figure 4C) showed good and single slice PET images (Supporting Figures S37, S38, and S39) moderate tumor-to-background contrast. The prolonged availability of the radiopeptide in the blood pool likely contributed to the observed plateau in tumor uptake seen in baseline conditions (Figure 4D upper panel), with no significant difference observed at 90 min post injection in the baseline and blocked conditions (baseline = 0.82 ± 0.14 SUV, *n* = 2, vs blocking = 0.76 ± 0.03 SUV, *n* = 2). As a possible contributor, close to 25% radioactivity in blood 60 min after administration of the other peptide analogue **[**^**18**^**F]14** was shown to be bound in RBCs *ex vivo*, contributing to the uptake in both tumor and nontarget tissues, such as the pancreas. This phenomenon, even when not studied for the more stable peptide **[**^**18**^**F]15**, possibly accounted for the low efficiency seen in the PET/CT study. Furthermore, due to the highly similar biological behaviors and relatively small differences of the TOC analogues **14** and **15**, the investigation of a non-PEGylated version would be warranted to assess the true benefit of adding a PEG chain to the structure.

### Dosimetry of **[**^**18**^**F]15**

The *ex vivo* biodistribution of **[**^**18**^**F]15** suggested certain organs were subject to elevated radiation burden. Regions of interest from the dynamic PET scans of **[**^**18**^**F]15** were used to estimate absorbed doses in selected organs, which were extrapolated to adult humans. Kidneys and the liver received the highest absorbed dose (kidney = 0.0366 ± 0.0016 mGy/MBq; liver = 0.0334 ± 0.0050 mGy/MBq) in baseline conditions, with negligible difference in the absorbed dose in blocking conditions (kidney = 0.0337 ± 0.0043 mGy/MBq; liver = 0.0313 ± 0.0040 mGy/MBq), as well as for all other organs. The second highest dose was in the adrenal glands (baseline = 0.0190 ± 0.0008 mGy/MBq; blocked = 0.0185 ± 0.0001 mGy/MBq) and the gallbladder wall (baseline = 0.0194 ± 0.0011 mGy/MBq; blocked = 0.0190 ± 0.0011 mGy/MBq) (Supporting Table S3 and Figure S32). The urinary bladder (baseline = 0.0134 ± 0.0003 mGy/MBq; blocked = 0.0135 ± 0.0000 mGy/MBq) and pancreas (baseline = 0.0156 ± 0.0002 mGy/MBq; blocked = 0.0154 ± 0.0001 mGy/MBq) received lower absorbed doses than those reported for the closest analogue [^18^F]AmBF_3_-TATE, for which the bladder received 0.027–0.030 mGy/MBq and the pancreas received 0.018–0.028 mGy/MBq.^17^ The dose in the lungs (0.006–0.013 mGy/MBq) for [^18^F]AmBF_3_-TATE reached near equal levels as to **[**^**18**^**F]15** (baseline = 0.0109 mGy/MBq), but the kidneys received a notably higher dose after administration of [^18^F]AmBF_3_-TATE (female, 1.24 mGy/MBq; male, 1.13 mGy/MBq) than after **[**^**18**^**F]15** (0.0334 mGy/MBq). All organs after administration of **[**^**18**^**F]15** received below 0.04 mGy/MBq dose, and apart from the kidneys and liver responsible for eliminating the radiotracer, all other organs received a dose of 0.02 mGy/MBq or below. The dosimetry calculation results indicate that the use of **[**^**18**^**F]15** as an imaging agent does not pose a greater radiation safety concern than that associated with other ^18^F-labeled SSTR radiotracers.

## Conclusions

We aimed to design a small tetrazine radiotracer that would harbor the beneficial characteristics of the zwitterionic trifluoroborate, including the excellent *in vivo* stability of fluorine-18 in the trifluoroborate moiety and the ease of IE radiolabeling. A novel AmBF_3_ tetrazine **[**^**18**^**F]6** was developed as a prosthetic group for radiolabeling biomolecules in mild conditions. Using two TCO-modified TOC derivatives as model peptides, we demonstrated that TCO-functionalized peptides can be radiolabeled using this method. While the development of novel SSTR2 radiotracers was not the goal of this investigation, the preconjugation of **6** with the TCO-modified peptide followed by radiolabeling might provide a radiopeptide of higher molar activity and hence potentially better performance. Nevertheless, the universal potential of **[**^**18**^**F]6** for the radiolabeling of biomolecule-based PET tracers by IEDDA bioorthogonal chemistry was corroborated. Future efforts should be aimed at radiolabeling a variety of biomolecules with **[**^**18**^**F]6**, especially those of higher molecular weight and more tolerant of the added hydrophobicity from the IEDDA cycloaddition product, for fully exploiting the benefits of this method. However, due to the optimal pharmacokinetics and radiolabel stability of **[**^**18**^**F]6** as a standalone tracer, the investigation of **[**^**18**^**F]6** in pretargeted PET imaging is warranted.

## Experimental Procedures

### Reagents and Equipment

Tetrazine NHS ester (BroadPharm, San Diego, CA), iodomethylboronic acid pinacol ester (Enamine, Riga, Latvia), TCO-*N*-hydroxysuccinimide (NHS) ester (Jena Bioscience, Jena, Germany), and TCO-PEG_3_-aldehyde (Conju-Probe, San Diego, CA) were used as received. Custom-synthesized aminooxy-functionalized Tyr^3^-octreotide was purchased from CSBio (Kelly Ct. Menlo Park, CA). Octreotide was purchased from Sigma-Aldrich (Saint Louis, Missouri, USA). Human plasma was received from Finnish Red Cross Blood Service, Helsinki, Finland (anonymous donor FFP-24). Heparin (5100 IU/mL) was purchased from Leo Pharma (Copenhagen, Denmark). Rat pancreatic tumor cell line AR42J (ATCC CRL-1492), expressing SSTR2, was obtained from the American Type Culture Collection (Manassas, VA, USA). Dry acetonitrile (DNA synthesis quality, max. 10 ppm H_2_O) was purchased from Sigma Aldrich (Supelco, Saint Louis, Missouri, USA). Sep-Pak C18-Light cartridges were purchased from Waters and PS-HCO_3_-cartridges (Macherey-Nagel, Düren, Germany) from Fisher Scientific (Waltham, MA). No-carrier-added ^18^F-fluoride was produced in-house with an IBA Cyclone 10/5 medical cyclotron from ^18^O-enriched water (≥97%) purchased from Rotem Industries Limited (Arava, Israel) and Campro Scientific (Berlin, Germany). The compounds were analyzed by nuclear magnetic resonance spectroscopy (NMR, 400 MHz Bruker Avance NEO NMR spectrometer) radio-thin-layer-chromatography (radio-TLC, silica; TLC silica gel 60 F_254_, reverse phase; Supelco TLC silica gel 60 RP-18 F_254_s) and radio-high-performance liquid chromatography (radio-HPLC) utilizing a diode array detector (DAD) and radiodetection (HPLC method A, Supporting Information). The radioactivity in organs was quantified by measuring with a Wizard γ counter. Positron emission tomography (PET) scans with computed tomography (CT) were acquired with a Molecubes PET (β-CUBE) coupled with a CT (X-CUBE) (Ghent, Belgium) (compound **[^18^F]15**) or Inveon (compound **[**^**18**^**F]6**). Detailed descriptions of the liquid chromatography methods are presented in the Supporting Information.

### Chemistry

#### Synthesis of AmBF_3_-Tz (6)

##### 2-[4-(1,2,4,5-Tetrazin-3-yl)phenyl]-*N*-[2-(dimethylamino)ethyl]acetamide (**3**)

*N*,*N*-Dimethylethylenediamine **2** (13 μL, 0.12 mmol) in 2 mL of DCM under argon and Tz NHS ester **1** (25 mg, 0.08 mmol) in 3 mL of DCM (added dropwise) were stirred at room temperature (1.5 h), evaporated to dryness, resuspended in water (1 mL), and purified with Sep-Pak Silica (MeOH:DCM 1:9). Pink solid **3** was obtained, with a yield of 68 ± 26% (*n* = 3) (11.5 mg, 0.04 mmol). ^1^H NMR (300 MHz, acetonitrile-*d*_3_) δ 10.26 (s, 1H), 8.50 (d, *J* = 8.4 Hz, 2H), 7.56 (d, *J* = 8.2 Hz, 2H), 3.60 (s, 2H), 3.26 (s, 2H), 2.40 (s, 2H), 2.21 (s, 6H).

##### 2-(2-(4-(1,2,4,5-Tetrazin-3-yl)phenyl)acetamido)-*N*,*N*-dimethyl-*N*-((4,4,5,5-tetramethyl-1,3,2-dioxaborolan-2-yl)methyl)ethan-1-aminium (**5**)

Compound **3** (11.5 mg, 0.04 mmol) in 1 mL of dry acetonitrile under argon and 2-(iodomethyl)-4,4,5,5-tetramethyl-1,3,2-dioxaborolane **4** (10.8 mg, 0.04 mmol) in 300 μL of dry acetonitrile were stirred at room temperature overnight and evaporated to dryness. Diethyl ether (2 mL) was added, and the flask was vortexed (30 s). The diethyl ether phase was discarded, and the residue was washed with diethyl ether twice, yielding 58 ± 31% (*n* = 3) (11.5 mg, 0.04 mmol) of compound **5**. ^1^H NMR (300 MHz, acetonitrile-*d*_3_) δ 10.28 (s, 1H), 8.52 (d, *J* = 8.3 Hz, 2H), 7.58 (d, *J* = 8.2 Hz, 2H), 3.68 (s, 2H), 3.58 (s, 2H), 3.48 (s, 2H), 3.13 (s, 6H), 2.14 (s, 2H), 1.28 (s, 12H).

##### {[(2-{2-[4-(1,2,4,5-Tetrazin-3-yl)phenyl]acetamido}ethyl)dimethylammonio]methyl}-trifluoroborate (AmBF_3_-Tz, **6**)

Compound **5** (18 mg, 0.043 mmol) in approximately 100 μL of acetonitrile was evaporated to dryness with argon gas flow the bottom of a 15 mL Falcon (LPDE) tube. DMF (1153 μL), water (387 μL), 4 M HCl (577 μL) and 3 M KHF_2_ (577 μL) was added into the 15 mL Falcon tube containing compound **5**.The tube was closed with septum and heated for 30 min at 70 °C. The reaction was monitored by HPLC (DAD detector 534 nm, 0.1%TFA-ACN:0.1% TFA-Milli-Q (80:20) isocratic 2.5 mL/min *t*_R_(AmBF_3_-Tz) = 10.3 min). The reaction mixture was diluted (water, 6 mL) and purified with two parallel SPE C18 PLUS cartridges (preconditioning: 5 mL of ACN, 10 mL of water) by washing with water (20 mL), dried with syringe infusion of air (10 mL), and eluted with ACN (1 mL) to afford 13.9 mg (yield 90%) of **6**. ^1^H NMR (400 MHz, CD_3_CN) δ 10.30 (s, 1H), 8.54 (d, *J* = 8.5 Hz, 2H), 7.58 (d, *J* = 8.6 Hz, 2H), 3.68–3.56 (m, 4H), 3.34 (t, *J* = 6.7 Hz, 2H), 3.01 (s, 6H), 2.38 (s, 2H). ^11^B NMR (128 MHz, CD_3_CN) δ 2.19, 1.80, 1.43, 1.03. ^19^F NMR (376 MHz, CD_3_CN) δ −138.77, −138.89, −139.04, −139.17. ^13^C NMR (101 MHz, CD_3_CN) δ 171.47, 167.25, 158.98, 141.95, 131.82, 131.42, 129.05, 118.30, 65.43, 54.32, 43.42, 34.75, 1.32. HRMS calculated for C_15_H_21_BF_3_N_6_O^+^ [M + H]^+^ 369.18165 m/z, found C_15_H_21_BF_3_N_6_O^+^ [M + H]^+^ 369.18134 *m*/*z* (mass error −0.85 ppm).

#### Synthesis of TCO-CHO

##### Synthesis of (*E*)-Cyclooct-4-en-1-yl (4-formylphenyl)carbamate(*trans*-cyclooctene aldehyde, **9**)

4-Aminobenzaldehyde (**7,** 15.6 mg,91 nmol, 1.5 equiv) was dissolved in THF (500 μL) and DMSO (150 μL) under argon.Pyridine (9.7 mg,122 nmol, 2.0 equiv) in THF (100 μL) was added to the solution of compound **7** and stirred for 10 min. (*E*)-cyclooct-4-enyl-2,5-dioxo-1-pyrrolidinyl carbonate (*trans*-cyclooctene-NHS ester, **8**, 16.3 mg, 61 nmol, 1.0 equiv) was dissolved in acetonitrile was added to the reaction mixture, and the solution was stirred overnight (room temperature). The reaction was monitored with TLC [normal-phase TLC, ethylacetate/cyclohexane, 1:1; KMnO_4_ stain; *R*_f_ (pyridine) = 0.00; *R*_f_ (benzaldehyde, **7**) = 0.00; *R*_f_ (TCO-NHS ester, **8**) = 0.90; *R*_f_ (TCO-CHO, **9**)] = 0.80. Fractionation: Sep-Pak SPE-Sil cartridge (preconditioning: 50 mL of water). The mixture was pushed through an SPE-Sil cartridge (fraction 1) and eluted with 1 mL of DCM (fraction 2), and the fractions were purified by semipreparative HPLC (Method B) yielding 21% ± 5% (*n* = 3). **9** eluted at *t*_R_ = 6 min on HPLC (Method B), on TLC (1:1 ethylacetate/cyclohexane, Sil-TLC + KMnO_4_ stain), at R*_f_* = 0.8. LC-MS (+) *m*/*z* (%) = 288.15942 *m*/*z* calculated for C_17_H_22_NO_3_^+^ and found 288.36 (27) [M + H]^+^, 310.14136 *m*/*z* calculated for C_17_H_21_NNaO_3_^+^ and found 310.30 (19) [M + Na]^+^ at *t*_R_ = 9.3 min. ^1^H NMR (400 MHz, CDCl_3_) δ ppm, 10.00, 7.86, 7.84, 7.45, 7.43, 5.53, 4.99, 4.41, 2.35, 1.97, 1.75, 1.57, 1.27, 1.26. ^13^C NMR (101 MHz, CDCl_3_) δ ppm, 191.81, 145.79, 135.64, 134.89, 133.01, 130.13, 127.77, 81.14, 44.70, 41.14, 38.67, 34.27, 32.50, 30.96.

#### *Trans*-Cyclooctene (TCO) Modification of Tyr^3^-Octreotide (TOC) and IEDDA

The peptide (1.4 mg, 1.08 μmol, 1 equiv) in 600 μL of 0.3 M anilinium acetate buffer (pH 4.6) was mixed with *trans-*cyclooctene-PEG_3_-aldehyde (**10**, 1.62 μmol, 1.5 equiv) or TCO-CHO (**11**, 1.64 μmol, 1.5 equiv) in ∼140 μL of chloroform and added dropwise. The reaction was monitored with HPLC (PDA detector at 280 nm). After 10 min, the peptide was purified with HPLC (method A). ACN was evaporated, and the residual water-containing fraction was frozen in a freezer (−80 °C) or with a liquid nitrogen bath. The frozen fraction was lyophilized and stored in a freezer (∼20 °C). The fractions, which were used as such, were mixed with **6** immediately as a diluted aqueous solution (diluted to ≥95% H_2_O). AmBF_3_-Tz (**6**, ∼200 nmol) in ACN (20 μL) was mixed at room temperature with TCO-PEG_4_-TOC (**12**, ∼200 nmol) or TCO-PEG_7_-TOC (**13**, ∼200 nmol) in water to constitute a solution ≥95% water. The resulting product, compound **14** or **15**, was purified by HPLC. The nonradiolabeled reference compounds **14** and **15** were analyzed by HPLC (method A) and MS (LC-MS or UHPLC-HRMS, methods C2 and D).

#### Radiosynthesis of [^18^F]AmBF_3_ Tetrazine (**[**^**18**^**F]6**)

Precursor **6** (∼37–74 μg, 100–200 nmol) in ACN (∼5 μL) and pyridazine HCl buffer pH 2.0 (10 μL) were pipetted into a 5 mL polypropylenetube fitted with a septum. Separate needles for transporting [^18^F]fluoride in to the tube and for venting the system through an ascarite cartridge and decay coil were connected to valves on the synthesis unit. [^18^F]Fluoride was transported to the hot cell and trapped with a PS-HCO_3_-cartridge (preconditioning sequence: 3 mL of water + 3 mL of brine + 3 mL of water). The precursor in the polypropylene tube was placed in a preheated (85 °C) thermomixer, and the [^18^F]fluoride was eluted with 100 μL of pyridazine HCl buffer pH 2.0. After heating (∼8 min) under an argon flow, the solution reached 10–20 μL reaction volume. The reaction mixture was quenched after 15 min at 85 °C with water (600 μL), diluted with water (8 mL), and purified, if not used as such, with a Sep-Pak C18 SPE cartridge. The cartridge was washed with water (40 mL) to remove [^18^F]fluoride. Air (10 mL) was pushed through the cartridge, and **[^18^F]6** was eluted out with ethanol (200 μL) and 0.9% NaCl (1 mL) or 0.01 M PBS (1 mL). The final product was analyzed with HPLC method A.

#### Radiosynthesis of [^18^F]AmBF_3_-Octreotides (**[**^**18**^**F]14** and **[**^**18**^**F]15**)

The crude radiolabeled mixture of [^18^F]AmBF_3_-Tz (**[**^**18**^**F]6**) was used for the radiolabeling of TCO-octreotides **12** and **13** without cartridge purification. *Trans-*cyclooctene functionalized peptide **12** or **13** (25–50 nmol, 500 μL of water) was added into the radiolabeling reaction mixture (∼10–20 μL) of **[**^**18**^**F]6** (100–200 nmol) and heated at 60 °C (95:5 H_2_O:ACN). After 20 min, the reaction mixture was diluted with water (8 mL) and purified with two SPE C18 cartridges [protocol: water (40 mL), 20% ethanol (3 mL), elution with 400 μL of ethanol and 400 μL of 10 × PBS]. The purified peptide solution was diluted with water to a 0.01 M PBS concentration and further with 1 × PBS to constitute ≤5% ethanol. The product was analyzed with HPLC method A.

### Lipophilicity, Hydrolytic Stability, and Enzymatic Stability

Lipophilicity was determined with the shake-flask method as a distribution coefficient between 0.01 M PBS and octanol. Purified radiotracer **[**^**18**^**F]6** (10 μL, ∼200 kBq, 1.5 nmol), **[**^**18**^**F]14** (20 μL, ∼260 kBq, 125 pmol), or **[**^**18**^**F]15** (20 μL, 144 kBq, 67 pmol) in a polypropylene tube containing 1500–2000 μL of each in a 1:1 mixture (1-octanol, 0.02 M PBS, pH 7.4) was shaken mechanically (500 rpm, 10 min) and centrifuged (1000*g*, 5 min), and samples from each layer (400 μL, *n* = 4) were measured with a γ-counter. The log D_7.4_ was calculated as a distribution between the two layers at pH 7.4. **[**^**18**^**F]6** (10 MBq, 40 nmol) diluted with 0.01 M PBS at pH 7.4 (≤1% ethanol, 5300 μL) was incubated for 180 min. At selected time points (5, 30, 60, 90, 120, 150, and 180 min), a sample (100 μL) was injected to radio-HPLC. **[**^**18**^**F]14** and **[**^**18**^**F]15** (29 MBq, 10 nmol) diluted in 2000 μL of 0.01 M PBS (≤1% ethanol) were left to incubate. A sample of **[**^**18**^**F]14** (100 μL) after 9 hours and **[**^**18**^**F]15** at selected time points between 30 and 335 min were injected into HPLC (method A). **[**^**18**^**F]15** (0.6 MBq, 0.134 nmol, 200 μL) formulated in ≤5% EtOH-1 × PBS was added and incubated in 2000 μL of PBS-50% human plasma at 37 °C. A 100 μL sample (at 60, 120, 180, and 240 min, *n* = 2) was diluted with 200 μL of cold acetonitrile and centrifuged (10,000*g*, 5 min). After centrifugation, a 100 μL sample of the supernatant was injected for radio-HPLC analysis.

### Cell Uptake Assay

AR42J cells were grown to >90% confluence. One million cells/well were seeded overnight on 6-well plates. The growth media was removed, and the reaction media (1 mL) containing **[**^**18**^**F]14** (11 kBq, 10 pmol per well) or **[**^**18**^**F]15** (97 kBq, 40 pmol per well) was added. Additionally, cells were coincubated with 2.4 nmol per well of nonmodified octreotide for blocking of radiotracer uptake. Radioactivity in the free, membrane-bound, and internalized fractions was determined at designated time points (15, 30, 60, and 120 min) by treating the cells in a sequence of (1) ice-cold 0.01 M PBS, (2) glycine buffer, and (3) 1 M NaOH, respectively. The fractions were collected and measured with a γ-counter. The detailed protocol is given in the Supporting Information.

### Animal Experimentation

The animal experiments were conducted under a project license approved by the National Board of Animal Experimentation in Finland (Helsinki; license number ESAVI/12132/04.10.07/2017). The animals were group-housed in polycarbonate cages using aspen bedding in HEPA-filtered housing units (UniProtect, Ehret, Emmendingen, Germany) with food (Envigo Teklad Global Diet 2016) and tap water available *ad libitum*. Conditions were maintained at 21 ± 1 °C and 55 ± 15% relative humidity with a 12:12 lighting cycle.

### Biological Evaluation

**[**^**18**^**F]14** and **[**^**18**^**F]15** in ≤4% ethanol–0.01 M PBS were administered intravenously (1.2 ± 0.0 and 2.0 ± 0.1 MBq, respectively, 0.2 nmol, 150 μL) to AR42J tumor-bearing Rj:NMRI-*Foxn1*^nu/nu^ mice. At predetermined time points (*t* = 30, 60, 120, and 240 min), animals were euthanized with CO_2_ asphyxiation and cervical dislocation, and then the organs were harvested, washed with water, and blotted dry, following with weighing and γ counting from which the % ID/g in tissues was determined.

#### Mouse Plasma Stability of [^18^F]AmBF_3_-PEG_7_-TOC (**[**^**18**^**F]15**) during *Ex Vivo* Studies

After tracer injection, CO_2_ asphyxiation, and cervical dislocation, blood was collected from a cardiac puncture into a tube containing 2 μL of 1% heparin (diluted from 5100 IU/mL) in 0.9% NaCl (aq.). The sample was centrifuged (1000*g*, 10 min) to separate the plasma from the blood cells. Cold acetonitrile (2 × vol of plasma) was added and centrifuged (10,000*g* for 5 min) to precipitate the proteins. A sample (100 μL) of the supernatant was injected into HPLC for radio-HPLC analysis. For the tracer **[**^**18**^**F]14**, the supernatant was sampled also on TLC for digital autoradiography analysis.

#### Distribution of Radioactivity in Blood Components after Intravenous Administration of **[**^**18**^**F]14**

Whole blood from mice were extracted during *ex vivo* studies, using cardiac puncture. The sample was applied in a microtube containing 1% heparin solution in 0.9% NaCl (aq.) (2 μL); the sample was centrifuged (1000*g*, 10 min), the total radioactivity in the sample was measured with a γ counter, the supernatant was separated from the pellet (RBC containing fraction), and cold ACN (500 μL) was added. The sample was centrifuged (10,000*g*, 5 min) to remove the free fraction from the precipitated protein-containing pellet. The pellet (protein-bound fraction) and the supernatant (free fraction) were measured with a γ counter, and a sample (100 μL) was injected into HPLC and spotted (4 μL) on a TLC plate (radio-TLC, TLC silica gel 60 F_254_, ACN/water 80:20).

#### PET/CT Imaging and Biodistribution after PET/CT of **[**^**18**^**F]6** and **[**^**18**^**F]15**

**[**^**18**^**F]6** in 10% ethanol–0.01 M PBS was administered intravenously to male Fox Chase SCID mice (CB.17 SCID) (11.0 ± 0.5 MBq, ∼11 nmol, ∼150 μL, *n* = 4) and healthy female C57BL/6JRj mice (11.3 ± 0.3 MBq, ∼11 nmol, ∼150 μL, *n* = 4) under 2% isoflurane anesthesia. The PET/CT image was acquired with Inveon PET/CT for 60 min followed by a 15 min static scan 4 h after injection of the tracer. The images of the dynamic scan were reconstructed to time frames 60 × 10, 10 × 60, 4 × 300, and 3 × 600 s. After the second PET imaging (*t* = 270 ± 1.9 min post injection, *n* = 4), the organs were harvested and the radioactivity in each tissue sample was measured with a γ-counter and reported as a percentage of injected dose per gram of tissue (% ID/g). **[**^**18**^**F]15** was formulated in 4% ethanol in 0.01 M PBS and administered intravenously (0.2 nmol, 0.80 ± 0.30 MBq; tracer: 150 μL; tracer + blocking dose: 200 μL) to AR42J tumor-bearing Rj:NMRI-*Foxn1*^nu/nu^ mice (*n* = 4) under 2% isoflurane anesthesia. PET/CT images were acquired with Molecubes PET (β-CUBE) coupled with a CT (X-CUBE) (MOLECUBES NV, Ghent, Belgium) with two mice being imaged at the same time under 2% isoflurane anesthesia. Images were reconstructed to time frames 30 × 10, 15 × 60, 4 × 300, and 5 × 600 s. Quantitative image analysis was done with Carimas software (v 2.10, Turku PET Centre, Turku, Finland). Spherical ROIs were hand-drawn based on anatomical CT data and PET signal for the desired organs. The results are presented as standardized uptake values (SUVs).

### Dosimetry

Dosimetry of **[**^**18**^**F]15** was calculated from the acquired PET/CT imaging data with Molecubes PET (β-CUBE) coupled with a CT (X-CUBE) (Ghent, Belgium). Regions of interest were drawn on source organs, namely, the heart, kidneys, liver, and lungs. Time–activity curves (TAC) were converted from mouse to human time −activity curves with the following equation
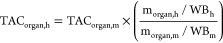
where m_organ,h_ and WB_h_ are the organ and whole-body weights for human, respectively. Mass m_organ,m_ and WB_m_ are the organ weight and the whole-body weight for mouse, respectively. Time–activity curves were normalized to 1 MBq injection, and the physical decay correction was removed. After this, the TAC’s were extrapolated into 3000 min, which corresponds in practice to infinity. Numbers of disintegrations in source organs are defined by integrating TAC from time 0 to 3000 min, and this value is input for OLINDA/EXM (version 2.1, Vanderbilt University, 2012) dosimetry software, where ICRP 89 reference adult male (73 kg) and ICRP 103 radiation weighting factors were used. Absorbed doses to each target organ are given in units mGy/MBq, and the effective dose is in units mSv/MBq.

### Statistical Analysis

The data were plotted and statistically analyzed with GraphPad Prism (version 9.1.1), and the results are presented as mean ± standard deviation (s.d.) with data points of *n* ≥ 3. The statistical analysis was done with the unpaired *t*-test with Welch′s correction, where *p* < 0.05 was regarded as statistically significant. The significances (*p*-value) were *p < 0.05, **p < 0.01, and ***p < 0.001.

## Supporting Information Available

LC analysis methods, cell uptake study protocol, characterization of compounds, metabolic stability profiles of compounds, *ex vivo* biodistribution studies, and additional PET/CT images (PDF)

## Notes

The animal experiments were conducted under a project license approved by the National Board of Animal Experimentation in Finland (Helsinki; license number ESAVI/12132/04.10.07/2017).
