# Supplementary material for: Development of [18F]AmBF3 Tetrazine for Radiolabeling of Peptides: Preclinical Evaluation and PET Imaging of [18F]AmBF3-PEG7-Tyr3-Octreotide in an AR42J Pancreatic Carcinoma Model
Source: Bioconjug Chem. 2022 Jun 16;33(7):1393–404. doi: 10.1021/acs.bioconjchem.2c00231 (PMC9305971; doi:10.1021/acs.bioconjchem.2c00231)
Supplement: Supplementary file 1 — bc2c00231_si_001.pdf [file bc2c00231_si_001.pdf]

Development of [^{18}F]AmBF$_3$ Tetrazine for Radiolabeling of Peptides: Preclinical Evaluation and PET Imaging of [^{18}F]AmBF$_3$-PEG$_7$-Tyr^3-Octreotide in AR42J Pancreatic Carcinoma Model

Sofia Otaru[†], Andreas Paulus[†], Surachet Imlimthan[†], Iida Kuurne[†], Helena Virtanen[‡], Heidi Liljenbäck[‡||], Tuula Tolvanen[‡⊥], Tatsiana Auchynnikava[‡§], Anne Roivainen[‡||], Kerttuli Helariutta[†], Mirkka Sarparanta[†], Anu J. Airaksinen[†,‡,§]*

[†]Radiochemistry, Department of Chemistry, University of Helsinki

[‡]Turku PET Centre, University of Turku

[§]Department of Chemistry, University of Turku

[||]Turku Center for Disease Modeling, University of Turku

[⊥]Department of Medical Physics, Turku University Hospital, Finland

*__Corresponding author__: Anu J. Airaksinen

Turku PET Centre, University of Turku, Kiinamyllynkatu 4-8, FI-20520 Turku, Finland

E-mail: anu.airaksinen@utu.fi

Author´s information

Kerttuli Helariutta

Present address: Department of Physics, University of Helsinki, FI-00014 Helsinki, Finland

Surachet Imlimthan

Present address: Inselspital University Hospital Bern, Nuclear Medicine, Freiburgstrasse 18 Bern, 3010, Switzerland

Affiliations

Radiochemistry, Department of Chemistry, P.O. Box 55, FI-00014, University of Helsinki, Helsinki, Finland

Sofia Otaru, Andreas Paulus, Surachet Imlimthan, Iida Kuurne, Kerttuli Helariutta, Mirkka Sarparanta, Anu J. Airaksinen

Turku PET Centre, Kiinamyllynkatu 4-8, University of Turku, FI-20520 Turku, Finland

Helena Virtanen, Heidi Liljenbäck, Tuula Tolvanen, Tatsiana Auchynnikava, Anne Roivainen, Anu J. Airaksinen

Department of Chemistry, University of Turku, FI-20014, Turku, Finland

Tatsiana Auchynnikava, Anu J. Airaksinen

Turku Center for Disease Modeling, Institute of Biomedicine, University of Turku, FI-20520 Turku, Finland

Heidi Liljenbäck, Anne Roivainen

Department of Medical Physics, Turku University Hospital, FI-20521 Turku, Finland

Tuula Tolvanen

Table of contents

Experimental procedures 6

Figure S1. ^{1}H NMR of AmBF$_3$-Tz (**6**). 10

Figure S2. HPLC-DAD of AmBF$_3$-Tz (**6**). 11

Figure S3. ^{19}F NMR of AmBF$_3$-Tz (**6**). 12

Figure S4. ^{11}B NMR of AmBF$_3$-Tz (**6**). 13

Figure S5. ^{13}C NMR of AmBF$_3$-Tz (**6**). 14

Figure S6. UHPLC-HRMS ESI(+) extracted ion chromatogram and mass spectrum of AmBF$_3$-Tz (**6**). 15

Figure S7. ^{1}H NMR of TCO-CHO (**9**). 16

Figure S8. ^{13}C NMR of TCO-CHO (**9**) 17

Figure S9. HPLC-DAD of TCO-CHO (**9**). 18

Figure S10. HPLC-DAD of TCO-functionalized octreotide (TCO-PEG$_4$-TOC, **12**) 19

Figure S11. HPLC-DAD of TCO-functionalized octreotide (TCO-PEG$_7$-TOC, **13**) 19

Figure S12. UHPLC-HRMS ESI(+) mass spectrum of TCO-PEG$_7$-TOC (**13**). 20

Figure S13. LC-MS spectrum of AmBF$_3$-PEG$_4$-TOC (**14**). 21

Figure S14. UHPLC-HRMS ESI(+) extracted ion chromatograms (EICs) and ESI MS spectra of AmBF$_3$-PEG$_7$-TOC (**15**). 22

Figure S15. The influence of temperature and reaction volume on the radiochemical yield (RCY%) of [^{18}F]AmBF$_3$-Tz (**[^{18}F]6**). 23

Figure S16. Radio-TLC of [^{18}F]AmBF$_3$-Tz (**[^{18}F]6**). 23

Figure S17. Radio-HPLC chromatogram of [^{18}F]AmBF$_3$-PEG$_4$-TOC (**[^{18}F]14**) 24

Figure S18. Radio-DAD-HPLC chromatogram of [^{18}F]AmBF$_3$-PEG$_7$-TOC (**[^{18}F]15**). 25

Figure S19. Hydrolytic stability of [^{18}F]AmBF$_3$-PEG$_4$-TOC (**[^{18}F]14**) at 9 hours in injection formulation. 26

Figure S20. Hydrolytic stability of [^{18}F]AmBF$_3$-PEG$_7$-TOC (**[^{18}F]15**) up to ~6 hours in injection formulation. 27

Injection formulation of **[^{18}F]15** comprised of 4% ethanol in 0.01 M phosphate-buffered saline.. 27

Figure S21. Cell-uptake of [^{18}F]AmBF$_3$-PEG$_4$-TOC (**[^{18}F]14**) in AR42J cells. 28

Figure S22. Cell-uptake of [^{18}F]AmBF$_3$-PEG$_7$-TOC (**[^{18}F]15**) in AR42J cells. 29

Figure S23. *Ex vivo* biodistribution (**A**, **B**) and elimination during PET/CT imaging (**C**) in selected organs and tissue samples of [^{18}F]AmBF$_3$-Tz (**[^{18}F]6**). 30

Figure S24. *Ex vivo* stability of [^{18}F]AmBF$_3$-PEG$_4$-TOC (**[^{18}F]14**) in AR42J tumor bearing mouse blood (Radio-HPLC). 31

Figure S25. *Ex vivo* stability of [^{18}F]AmBF$_3$-PEG$_4$-TOC (**[^{18}F]14**) in urine (Radio-HPLC). 32

Figure S26. *Ex vivo* stability of [^{18}F]AmBF$_3$-PEG$_4$-TOC (**[^{18}F]14**) in AR42J tumor bearing mouse blood 5 and 30 minutes post-injection (Radio-TLC). 33

Table S1. *Ex vivo* blood component distribution analysis from mouse blood after intravenous administration of **[^{18}F]14**. 33

Table S2. Biodistribution and statistical analysis of **[^{18}F]14** and **[^{18}F]15** at 60 minutes after i. v. administration expressed as %ID/g. 34

Figure S27. *Ex vivo* biodistribution of [^{18}F]AmBF$_3$-PEG$_4$-TOC (**[^{18}F]14**) (*ex vivo*; 60 minutes post-injection and 30, 60 and 120 min post-injection). 35

Figure S28. *Ex vivo* biodistribution of [^{18}F]AmBF$_3$-PEG$_7$-TOC (**[^{18}F]15**) (*ex vivo*; at 30 min, 60 min and 120 min post-injection, *ex vivo* after PET/CT; 270 min). 36

Figure S29. Tumor-to-muscle (T/M) and tumor-to-blood (T/B) ratios of **[^{18}F]14** after *ex vivo* biodistribution ($n \geq 4$). 37

Figure S30. Tumor-to-muscle (T/M) and tumor-to-blood (T/B) ratios of [^{18}F]AmBF$_3$-PEG$_7$-TOC (**[^{18}F]15**) after *ex vivo* biodistribution ($n \geq 2$). 38

Figure S31. Tumor-to-muscle (T/M) and tumor-to-blood (T/B) ratios of [^{18}F]AmBF$_3$-PEG$_7$-TOC (**[^{18}F]15)** during PET/CT imaging. 39

Table S3. Dosimetry calculations. 40

Figure S34. Standalone single-slice blank-on-white PET images of **[^{18}F]6** in a male SCID mouse. 43

Figure S35. Standalone single-slice blank-on-white PET images of **[^{18}F]6** in a healthy female C57BL/6JRj mouse 43

Figure S36. Single-slice coronal PET/CT images of **[^{18}F]6** in (**A**) male SCID and (**B**) healthy female C57BL/6JRj mice. 44

Figure S37. Standalone single-slice blank-on-white PET images of **[^{18}F]15** in baseline conditions studied in a Rj:NMRI-Foxn1$^{nu/nu}$ mouse bearing subcutaneous rat pancreatic AR42J xenografts. 45

Figure S38. Standalone single-slice blank-on-white PET images of **[^{18}F]15** in blocking conditions studied in aRj:NMRI-Foxn1$^{nu/nu}$ mouse bearing subcutaneous rat pancreatic AR42J xenografts. 45

Figure S39. Single-slice coronal PET/CT images of **[^{18}F]15** in baseline (**A**) and blocking (**B**) conditions studied in Rj:NMRI-Foxn1$^{nu/nu}$ mice bearing subcutaneous rat pancreatic AR42J xenografts. 46

Experimental procedures

PET/CT imaging

The standardized uptake values (SUVs) were determined from the PET image by drawing regions-of-interest (ROI) around selected organs (heart, liver, kidney, lung, muscle, urinary bladder) with Carimas software (version 2.10, Turku PET Centre, Turku, Finland) and by measuring the ratio of radioactivity per unit volume of that ROI and normalized to the injected dose. Compound **[^{18}F]6** was imaged with Inveon® PET/CT (Siemens) and compound **[^{18}F]15** with Molecubes PET (β-CUBE) coupled with a CT (X-CUBE) (MOLECUBES NV, Ghent, Belgium).

Liquid chromatography and mass spectrometry methods

HPLC analysis (Radiodetectotion/Diode-array detection): Shimadzu HPLC system containing a DGU-20A degasser, an LC-20AD UPLC LC unit, a SIL-20A HT autosampler, a CTO-20AC column oven, a CBM-20A communications bus module, a Scionix Holland scintillation detector with a 51 BP 51/2 NaI(Tl) crystal and an SPD-M20A diode array detector.

UHPLC-HRMS analysis: The compounds **6**, **13** and **15** were analysed by UHPLC Thermo Scientific Dionex Ultimate 3000 ultrahigh performance liquid chromatography (Germering, Germany) coupled to Thermo Scientific Orbitrap Fusion mass spectrometer (San Jose, CA, USA). Ionization (HESI+), scan range of 120–1200 *m/z.* Data was processed with Xcalibur workstation (Thermo Fisher Scientific, Waltham, MA, USA). Compounds **6** and **13** were analysed for acquiring their exact masses. Compound **15** was analyzed for acquiring its exact mass and isotopic peak patterns.

LC-MS analysis: Compounds **12** and **14** were analyzed with Agilent Technologies 1260 Infinity HPLC-DAD system with Agilent Technologies 6120 Quadruple LC/MS detector.

Ionization HESI$^+$, scan range 100-2000 *m/z*. Data was processed with OpenLAB CDS Workstation.

Liquid chromatography (LC) method gradients:

Method A (HPLC and radio-HPLC): (Shimadzu), Column: Phenomenex Kinetex® 5 µm C18 100 Å, LC Column 250 × 10.0 mm

Eluents: (A) H_2O+0.1% TFA and (B) ACN+0.1% TFA

Gradient 3 mL/min at ambient temperature:

Time (min)	%B
0	20
20	50
23	20
25	20
32	20

Method B (HPLC): (Shimadzu), Column: HiChrom (Alltech) Alltima® 5 µm C18 100 Å, LC Column 250 × 10.0 mm

Eluents: (A) H_2O+0.1% TFA and (B) ACN+0.1% TFA

Gradient 3 mL/min at ambient temperature: Isocratic

Time (min)	%B
0	80
32	80

Method C (LC-MS): (Agilent 1200 Series), Column: Waters Atlantis® T3 3 µm C18 100 Å, LC Column 4.6 × 150 mm

Method 1C) for compound **9** (t_R = 11.5 min)

A: 0.1 % TFA in milli-Q water
B: 0.1 % TFA in ACN
Flowrate: 0.7 mL/min

Time (min)	%B
0	50
15	90
26	10

Method 2C) for compounds **12** (t_R = 12.8 min), **14** (t_R = 11.6 min) and **15** (t_R = 15.2 min) **(Agilent 1200 Series)**

A: 0.1 % FA in milli-Q water
B: 0.1 % FA in ACN
Flowrate: 0.7 mL/min

Time (min)	%B
0	10
33	90
35	90
40	10

Method D (UHPLC-HRMS): (Orbitrap Fusion)

Column: Waters ACQUITY UPLC® 1.7 µm BEH C18 130Å, UPLC Column 2.1 × 50 mm

Eluents: (A) H_2O+0.1% FA and (B) ACN+0.1% FA

Gradient 0.5 mL/min at 40 °C:

Scan range: 120-1200 *m/z* for compound **6** and 100-2000 *m/z* for compounds **13** and **15**.

For compound **6** and **15**:

Time (min)	%B
0	5
3	5
4	25
5	60
6	80
7	100
7.5	5
9	5

Lipophilicity. The lipophilicities were determined with the shake-flask method as a distribution profile between 0.01 M PBS and octanol at pH 7.4. The distribution of the radiolabeled compound between the octanol and the PBS layers was calculated according to equation:

$$\mathrm{Log}D_{7.4} = \frac{\mathrm{Ac}_{\mathrm{octanol}}}{\mathrm{Ac}_{\mathrm{PBS}}}$$

where Ac_{oct}=radioactivity concentration of octanol and Ac_{PBS}=radioactivity concentration of PBS. The datapoints were presented as mean ± standard deviation.

AR42J cell-uptake assay and mouse tumor model. Rat pancreatic tumor cell line AR42J that expresses SSTR2, was cultured at 37 °C in 95% relative humidity incubator containing 5% CO_2. Cells grown to >90% confluence were used for either *in vitro* or *in vivo* experiments. One million cells/well were seeded overnight on 6-well plates. The growth media was removed and the medium (1 mL/well) containing **[^{18}F]14** (11 kBq, 10 pmol per well) or **[^{18}F]15** (97 kBq, 40 pmol per well) was added. For studying the specificity of the cell-uptake, a set of cells were co-incubated in the presence of 2.4 nmol per well of non-modified octreotide representative of blocking conditions (total volume ~1 mL). For determining the amount of the radiotracer in the *free fraction*, at the designated time-points (15, 30, 60 and 120 min) the incubated media were removed, collected to a 5 mL scintillation tube, cells were washed with 1 mL of cold 0.01 M PBS (1×PBS) and again the supernatant collected into the same scintillation tube. *Membrane-bound fraction*; ice-cold glycine buffer (1 mL) was added on the cells, incubated for 5 minutes on ice, supernatant removed (procedure repeated once more) and the cells washed with ice-cold 1×PBS, and all washing solutions were collected to the same scintillation tube. *Internalized fraction*; 1 M NaOH (1 mL) was added on the cells and left incubating at an ambient temperature for 10 min. The supernatant was removed, the wells were washed 2 × 1 mL of cold 0.01 M PBS, and the supernatants collected to the same microtube. The collected samples were measured with a gamma counter for measuring the counts per minute (CPM) of each fraction (n = 3). The internalization is presented in percent (%) normalized to the total radioactivity in all three fractions.

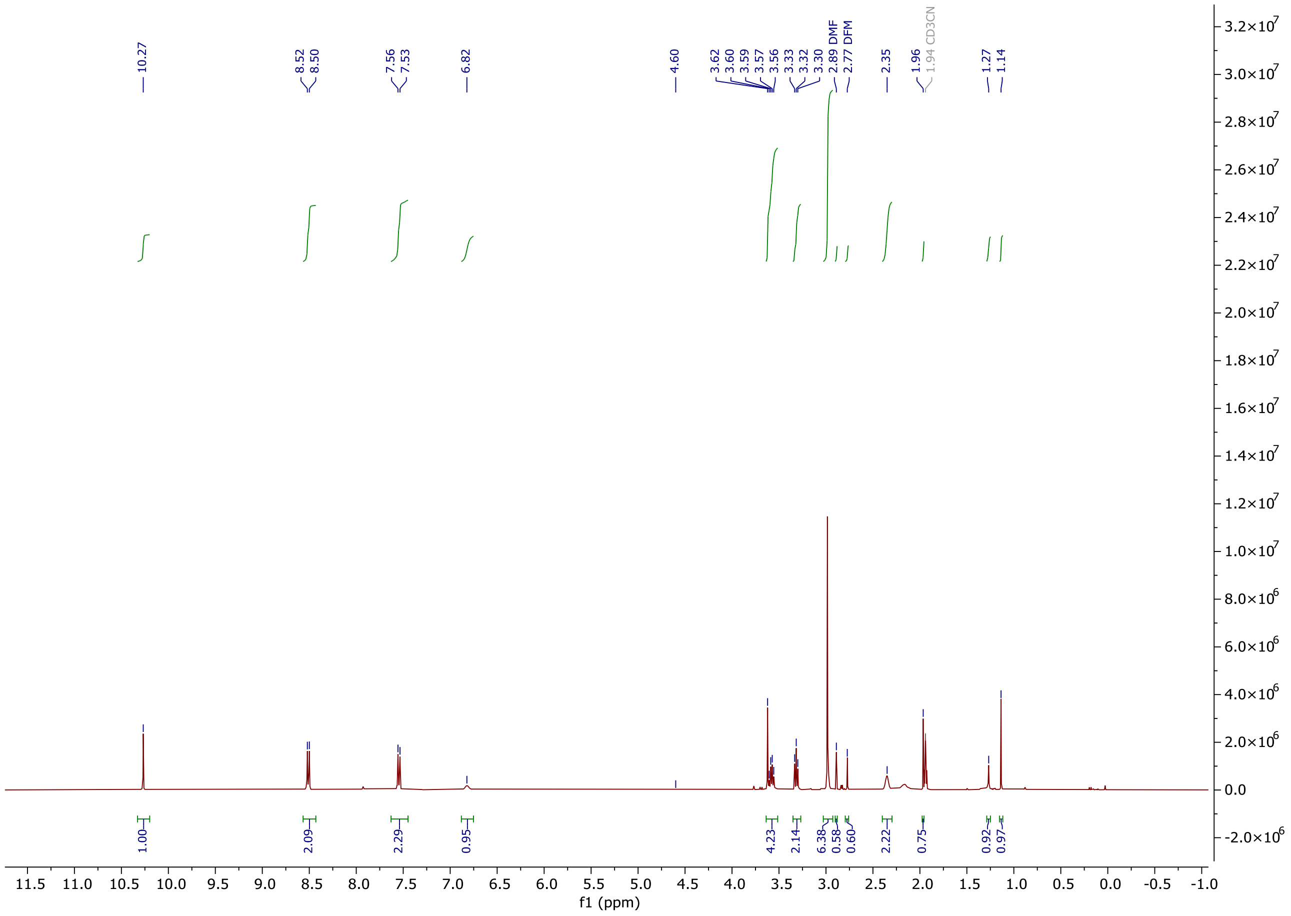

Figure S1. ^{1}H NMR of AmBF$_3$-Tz (**6**).

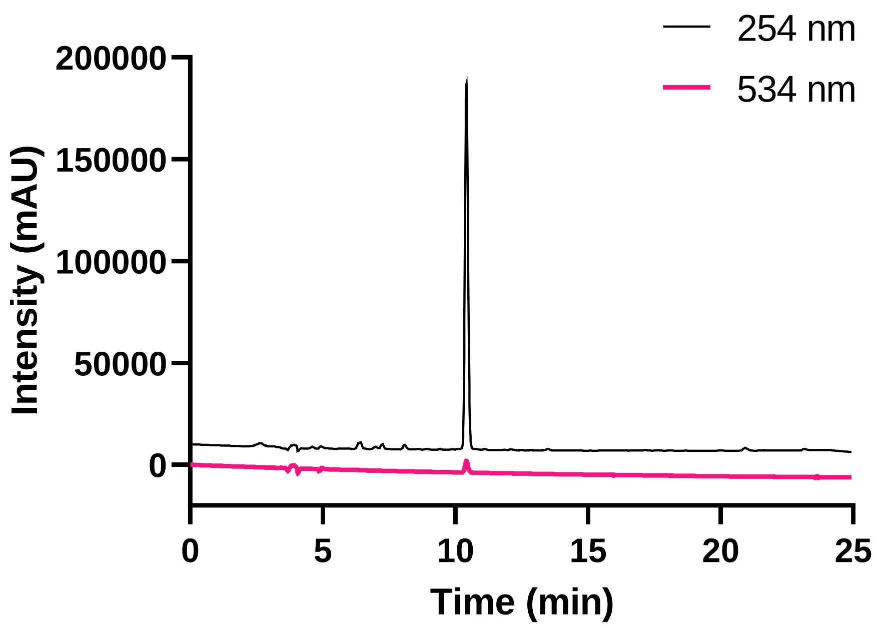

Figure S2. HPLC-DAD of $AmBF_3$-Tz (**6**).

Purity ≥99%.

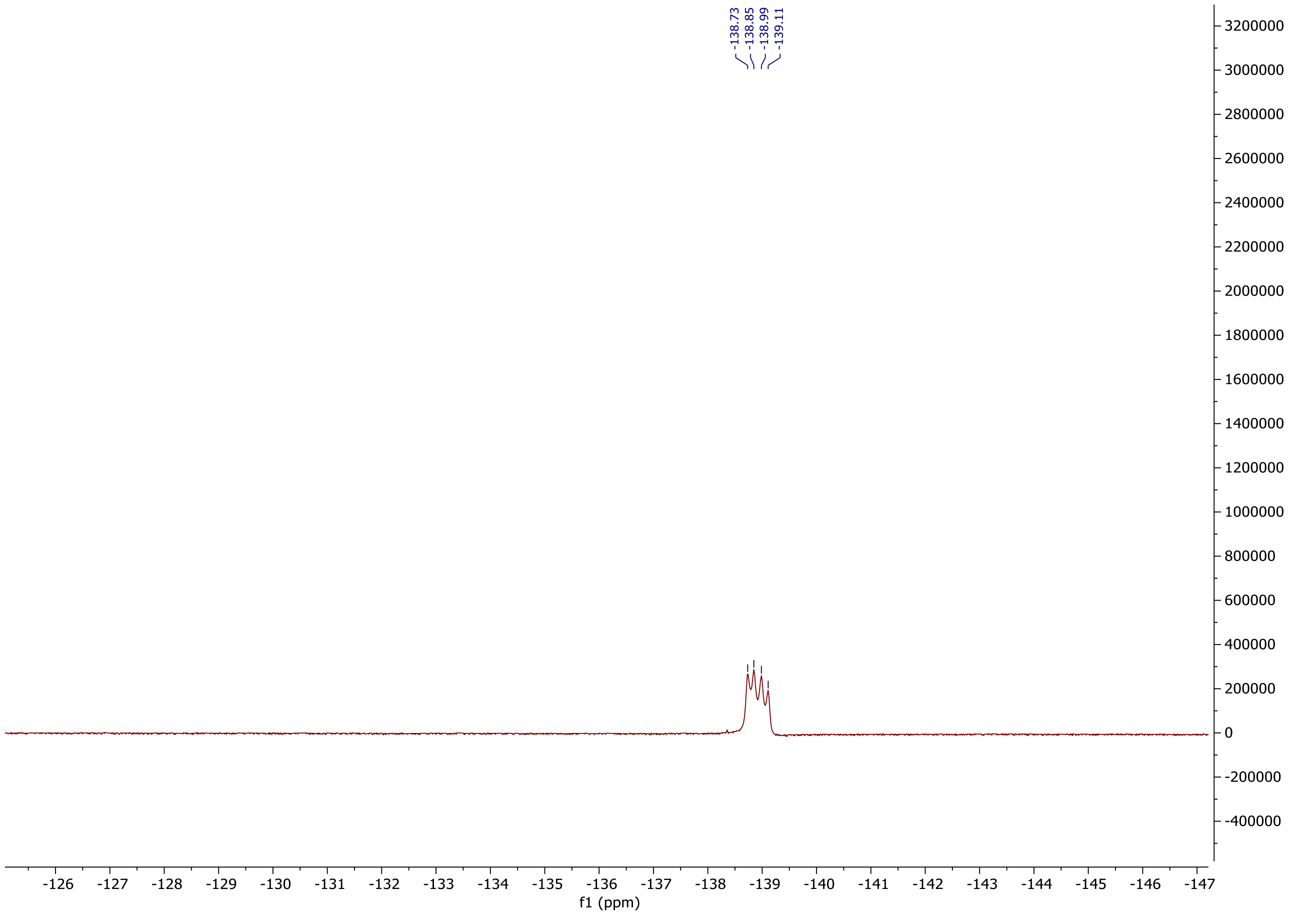

Figure S3. ^{19}F NMR of $AmBF_3$-Tz (**6**).

The signal as quartet demonstrates ^{19}F-^{11}B coupling with characteristic ratios (1:1:1:1).

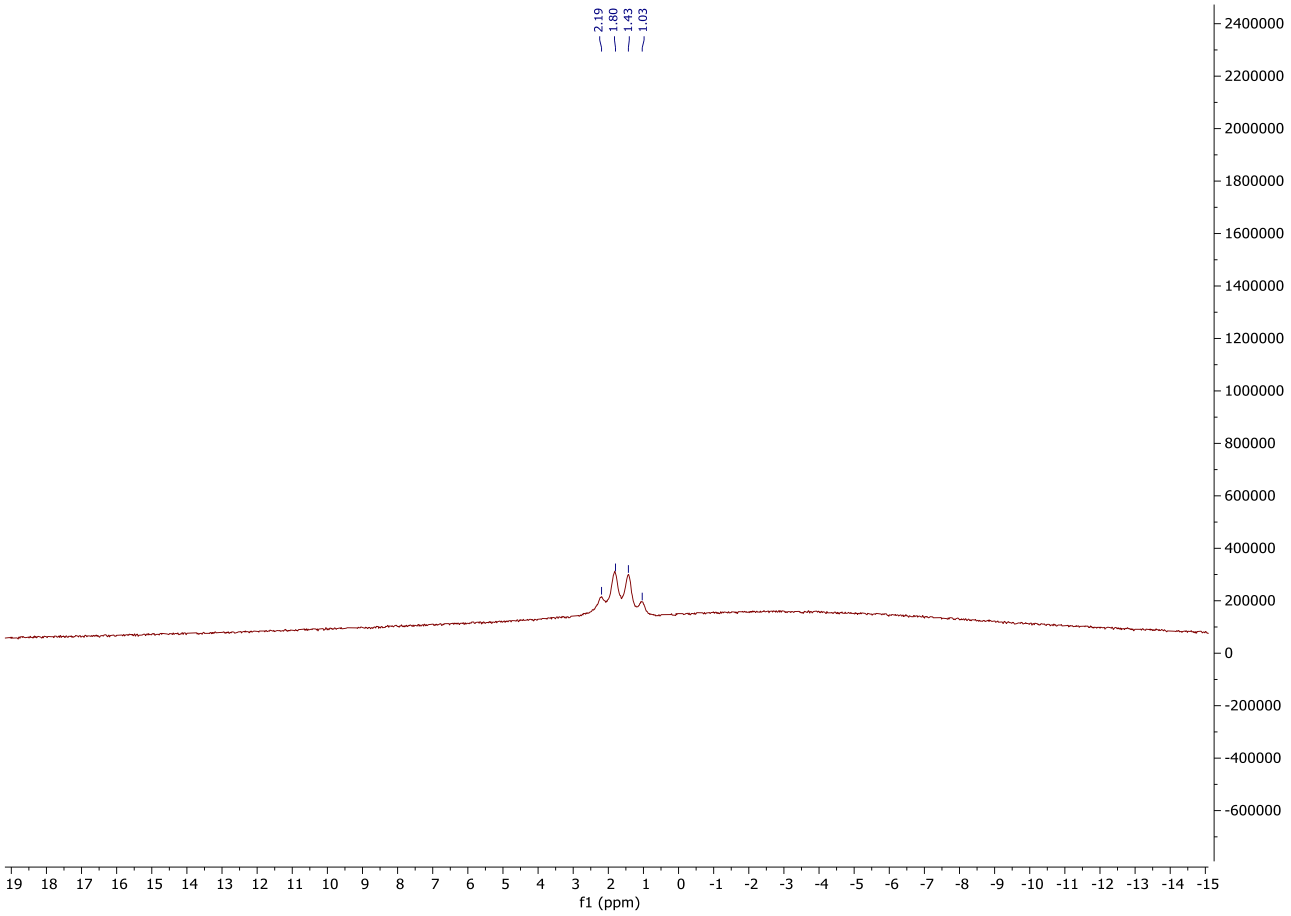

Figure S4. ^{11}B NMR of $AmBF_3$-Tz (**6**).

The signal as quartet demonstrates ^{11}B-^{19}F coupling with characteristic ratios (1:3:3:1).

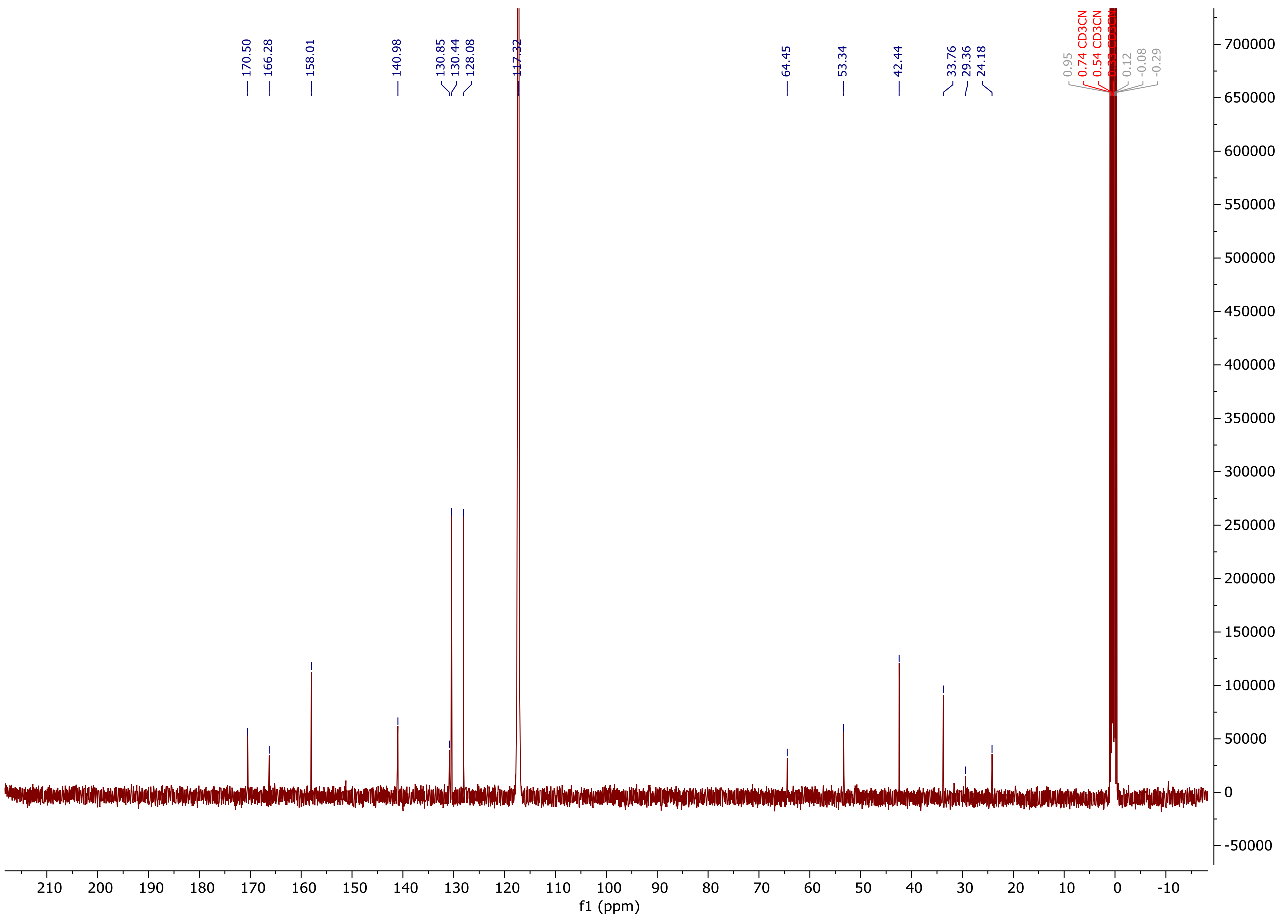

Figure S5. ^{13}C NMR of AmBF$_3$-Tz (**6**).

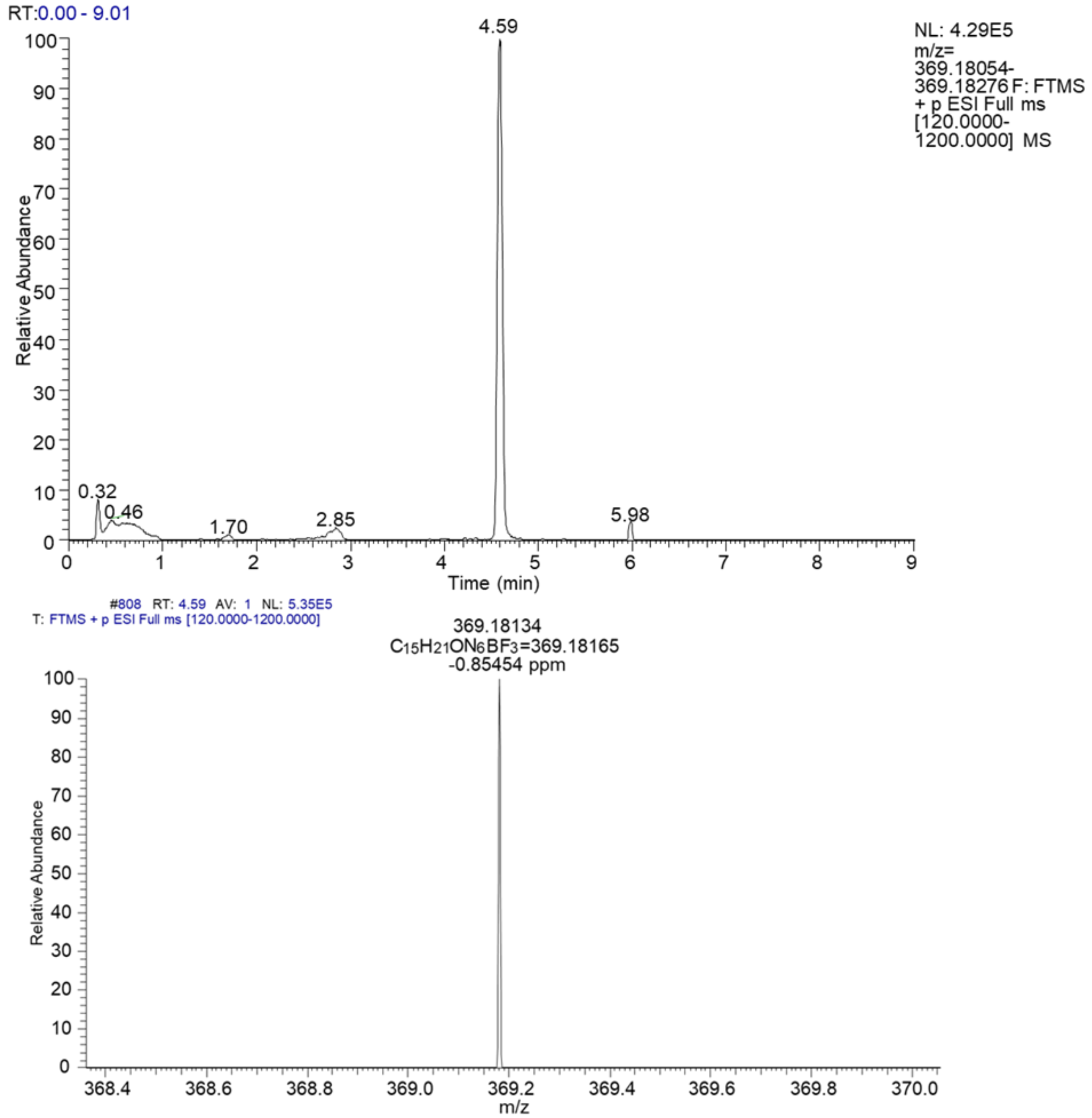

Figure S6. UHPLC-HRMS ESI(+) extracted ion chromatogram and mass spectrum of AmBF$_3$-Tz (**6**).

6 detected as a protonated molecule ($[M+H]^+$ found *m/z* 369.18134, mass error Δ=-0.85454 ppm, calculated *m/z* 369.18165 for $C_{15}H_{21}N_6BF_3O^+$

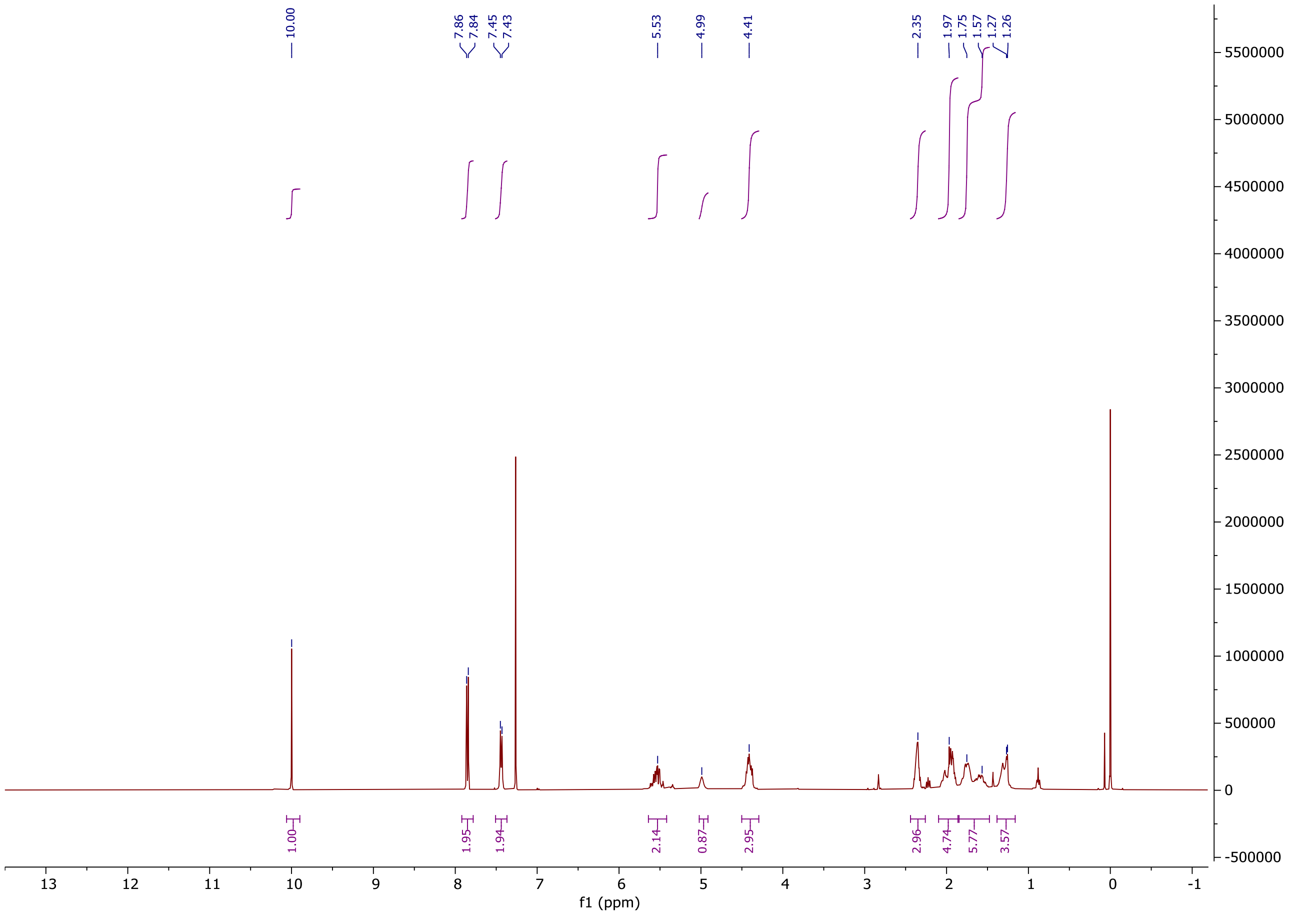

Figure S7. ^{1}H NMR of TCO-CHO (**9**).

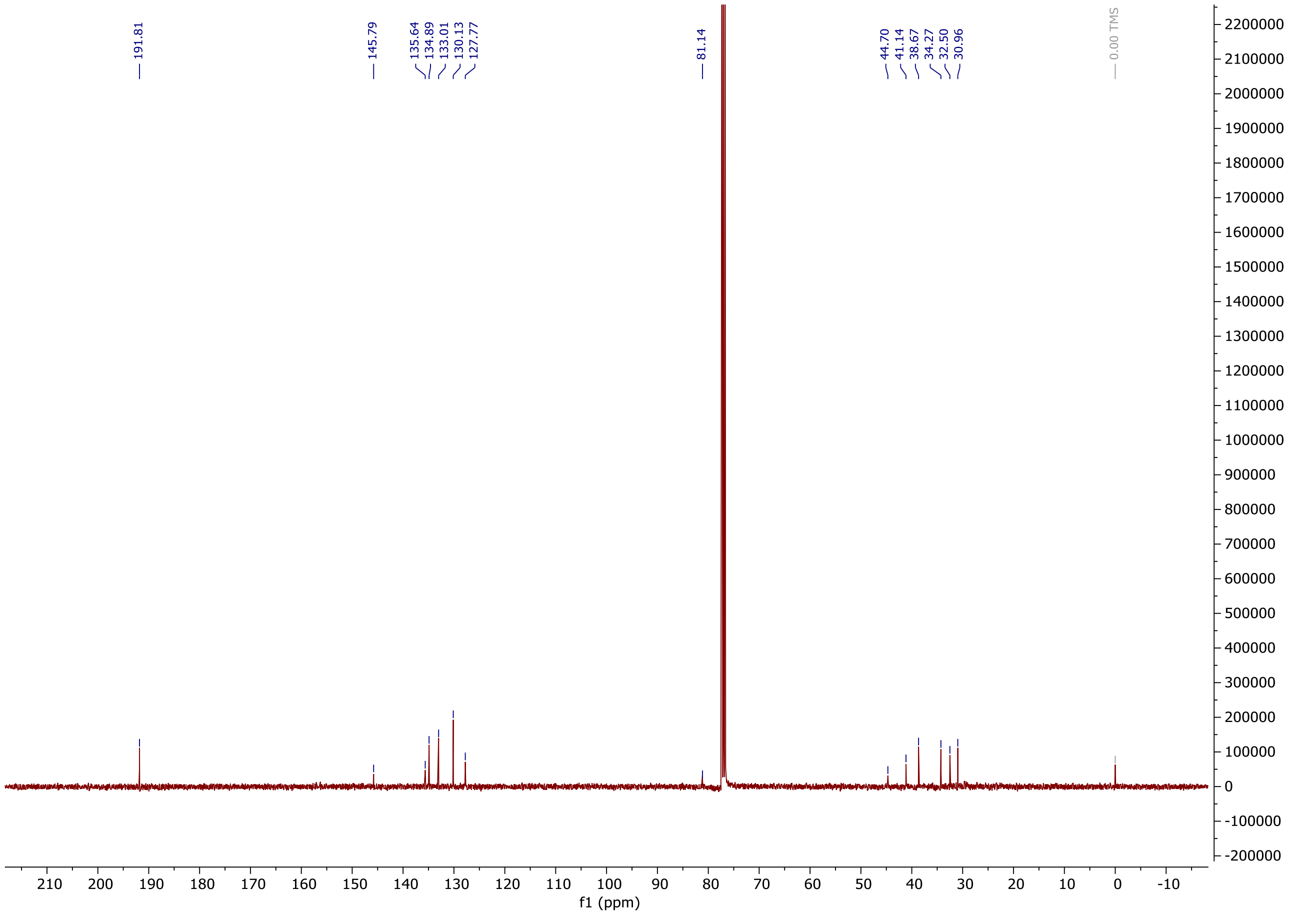

Figure S8. ^{13}C NMR of TCO-CHO (**9**).

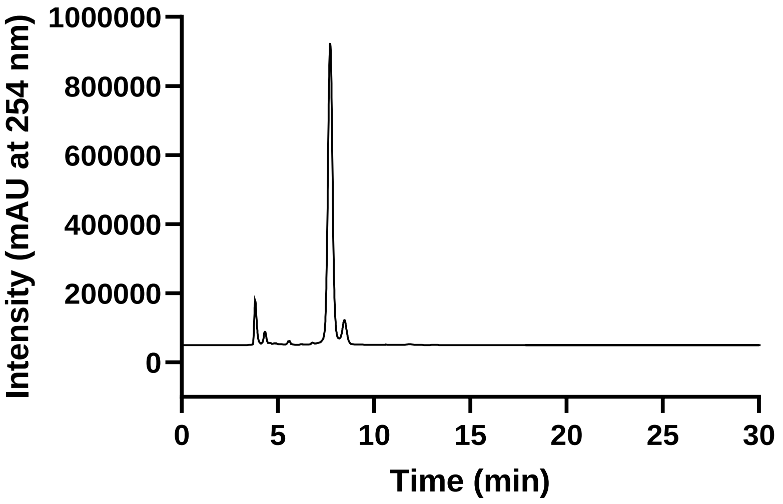

Figure S9. HPLC-DAD of TCO-CHO (**9**).

Retention time of **9** t_R = 7.8 min. Residual pyridine peak detected at 3.8 min. Purity ≥98%.

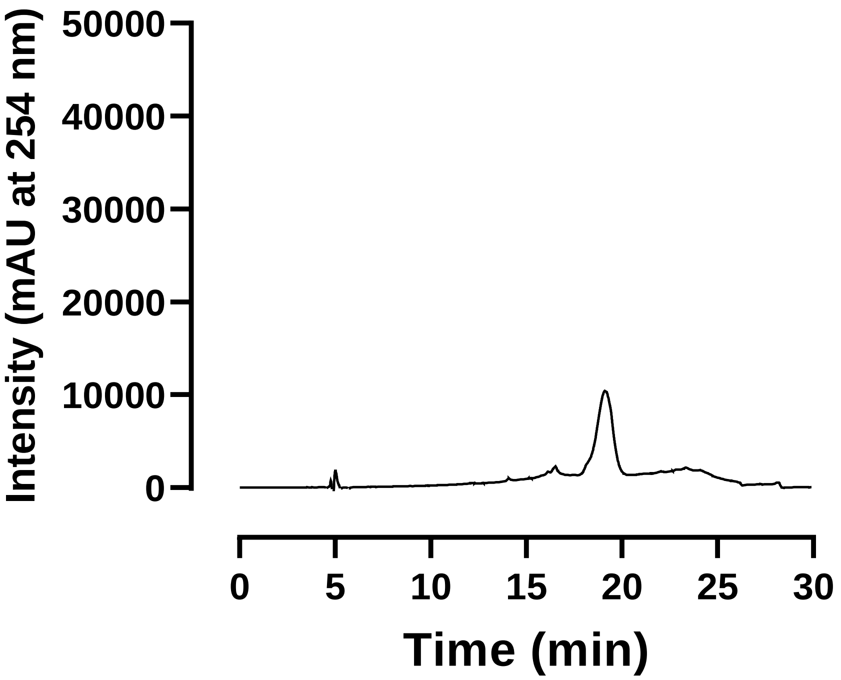

Figure S10. HPLC-DAD of TCO-functionalized octreotide (TCO-PEG$_4$-TOC, **12**) Retention time of **12** t_R = 19.1 min.

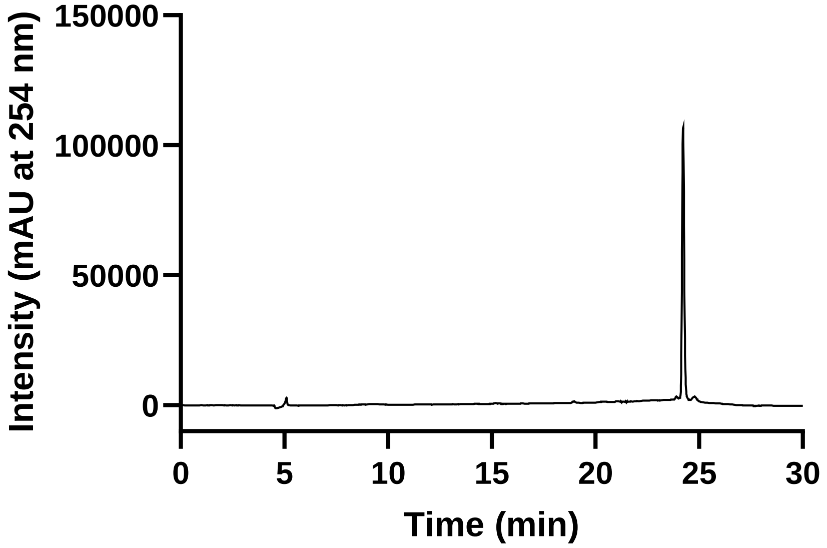

Figure S11. HPLC-DAD of TCO-functionalized octreotide (TCO-PEG$_7$-TOC, **13**) Retention time of **13** t_R = 24.1 min.

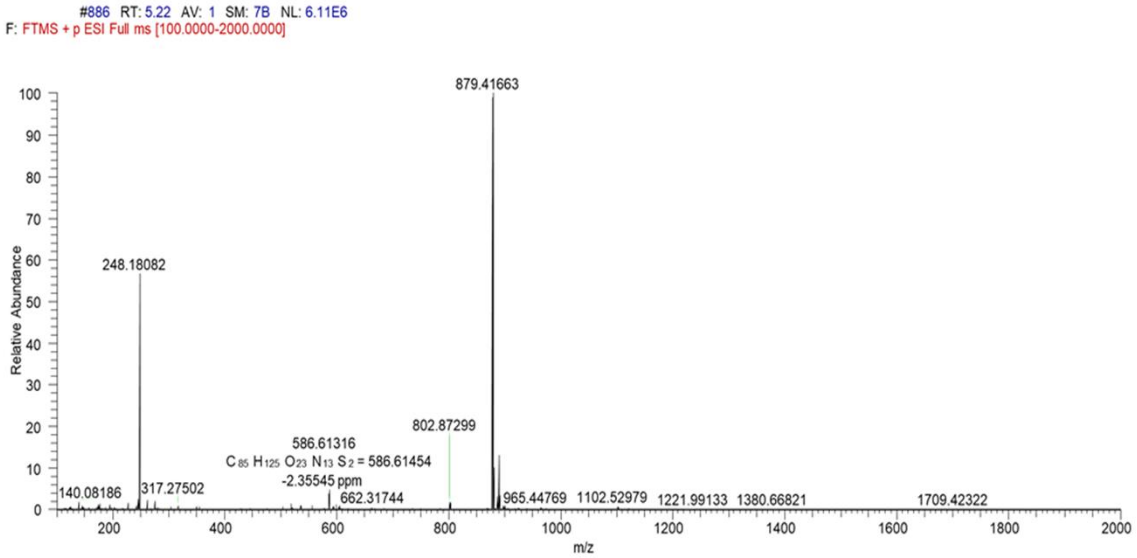

Figure S12. UHPLC-HRMS ESI(+) mass spectrum of TCO-PEG$_7$-TOC (**13**).

Compound **13** eluted at t_R = 5.22 min, detected as two protonated molecule ions: $[M+3H]^{3+}$ at 586,61316 *m/z* with Δ = -2,35545 ppm (calculated 586,61454 *m/z* for $C_{85}H_{125}O_{23}N_{13}S_2^{3+}$), and $[M+2H]^{2+}$ at 879,41663 *m/z* with Δ = -1,75922 ppm (calculated 879,41817 *m/z* for $C_{85}H_{124}O_{23}N_{13}S_2^{2+}$).

Peak RT : 11.644 min **Area % :** 18.62%

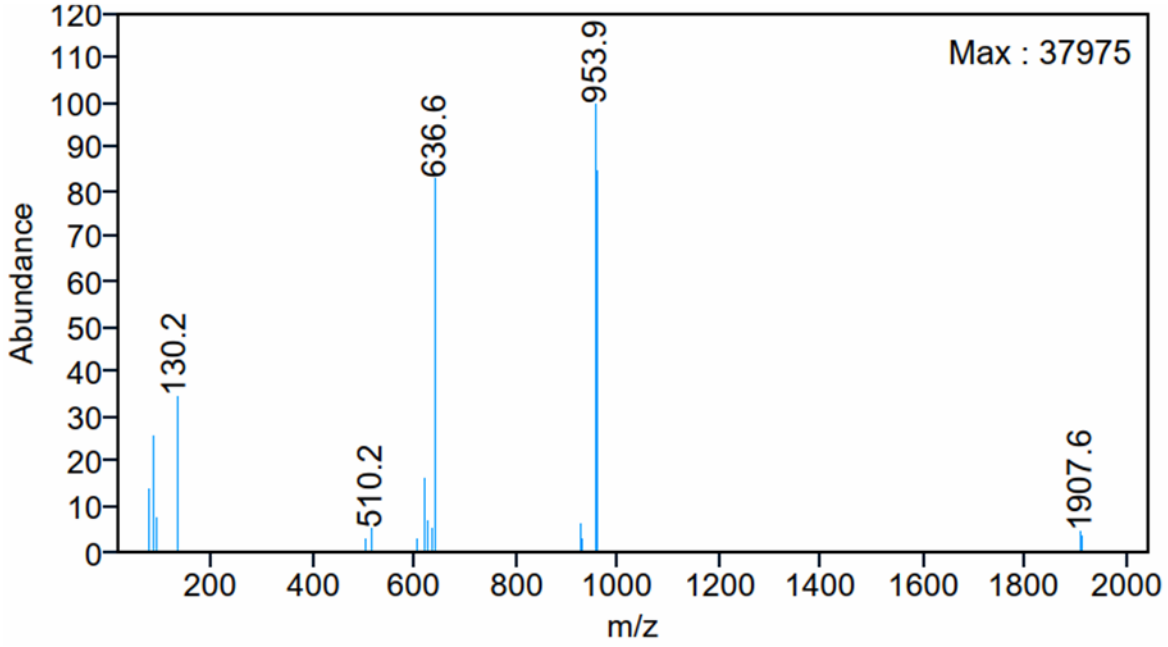

Figure S13. LC-MS spectrum of AmBF$_3$-PEG$_4$-TOC (**14**).

Retention time of **14** t_R = 11.644 min**. 14** was detected as molecular peaks corresponding to three charged states (Z=+1 - Z=+3) $[M+H]^+$ (found *m/z* 1907.6, calculated *m/z* 1907.9 for $C_{92}H_{125}BF_3N_{16}O_{20}S_2^+$), $[M+2H]^{2+}$ (found *m/z* 953.9, calculated *m/z* 953.9 for $C_{92}H_{126}BF_3N_{16}O_{20}S_2^{2+}$) and $[M+3H]^{3+}$ (found *m/z* 636.6, calculated *m/z* 636.6 for $C_{92}H_{127}BF_3N_{16}O_{20}S_2^{3+}$).

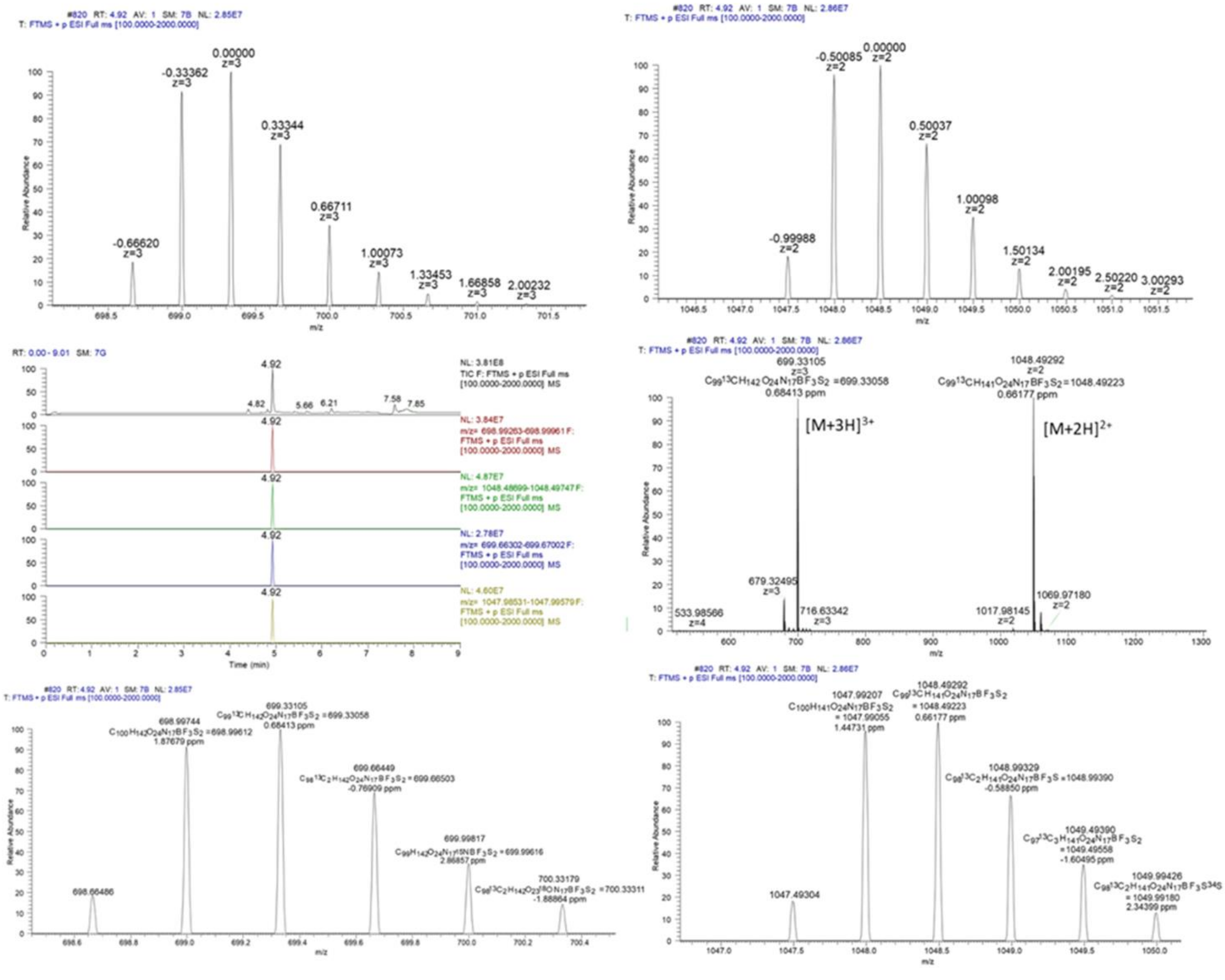

Figure S14. UHPLC-HRMS ESI(+) extracted ion chromatograms (EICs) and ESI MS spectra of AmBF$_3$-PEG$_7$-TOC (**15**).

Compound **15** was detected as well resolved isotopic molecular ion peaks, with a pattern demonstrating mass differences of 0.33 and 0.50 *m/z* for the detected molecular peaks, indicative of charged state at Z=+3 for 0.33, and a charged state of Z=+2 for 0.50 mass difference. The exact monoisotopic masses for $C_{100}H_{141}BF_3O_{24}N_{17}S_2^{2+}$ $[M+2H]^{2+}$ was found at *m/z* 1047.99207 (Δ = 1.44731 ppm, calculated *m/z* 1047.99055) and of $C_{100}H_{142}BF_3O_{24}N_{17}S_2^{3+}$ for $[M+3H]^{3+}$, was found at *m/z* 698.99744 (Δ = 1.87679 ppm, calculated *m/z* 698.99612), correlated with the calculated masses of compound **15**.

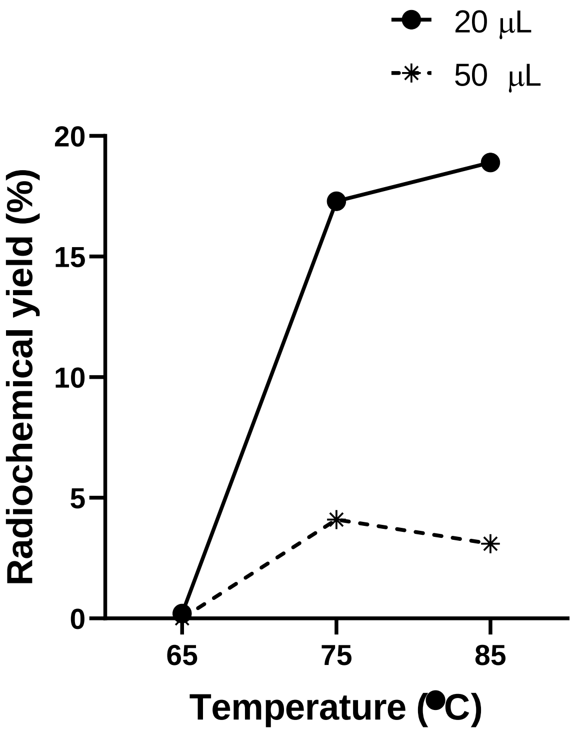

Figure S15. The influence of temperature and reaction volume on the radiochemical yield (RCY%) of [^{18}F]AmBF$_3$-Tz (**[^{18}F]6**).

Reaction with 0.9% NaCl eluted [^{18}F]fluoride.

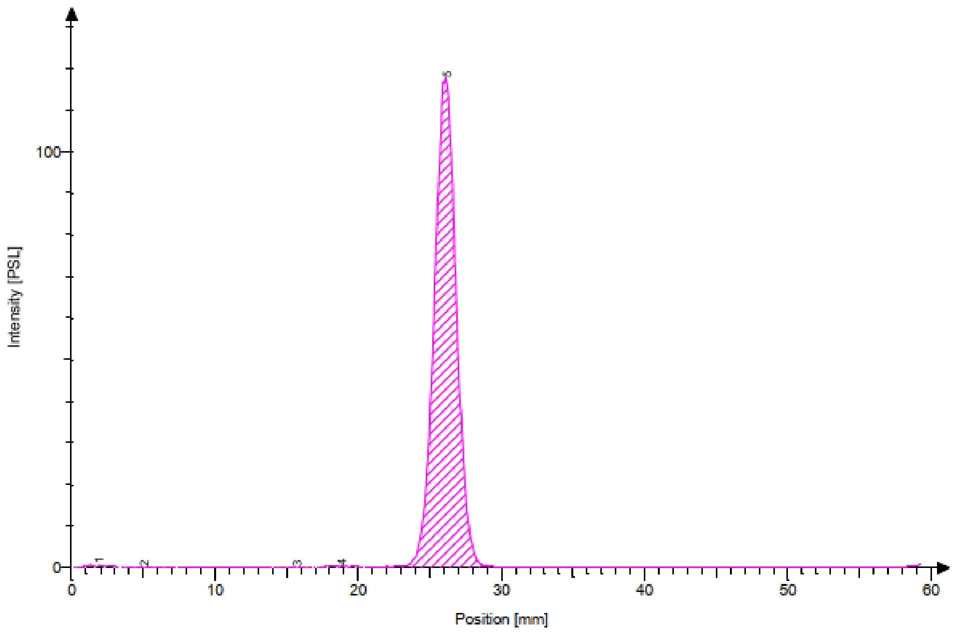

Figure S16. Radio-TLC of [^{18}F]AmBF$_3$-Tz (**[^{18}F]6**).

RCP ≥98%.

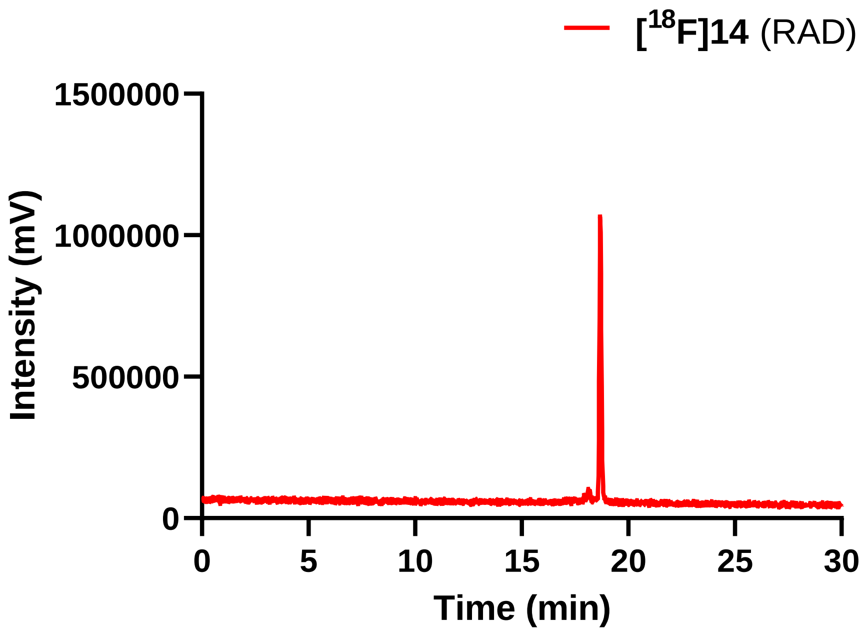

Figure S17. Radio-HPLC chromatogram of [^{18}F]AmBF$_3$-PEG$_4$-TOC (**[^{18}F]14**). Retention time of **14** t_R = 18.7 min (mV).

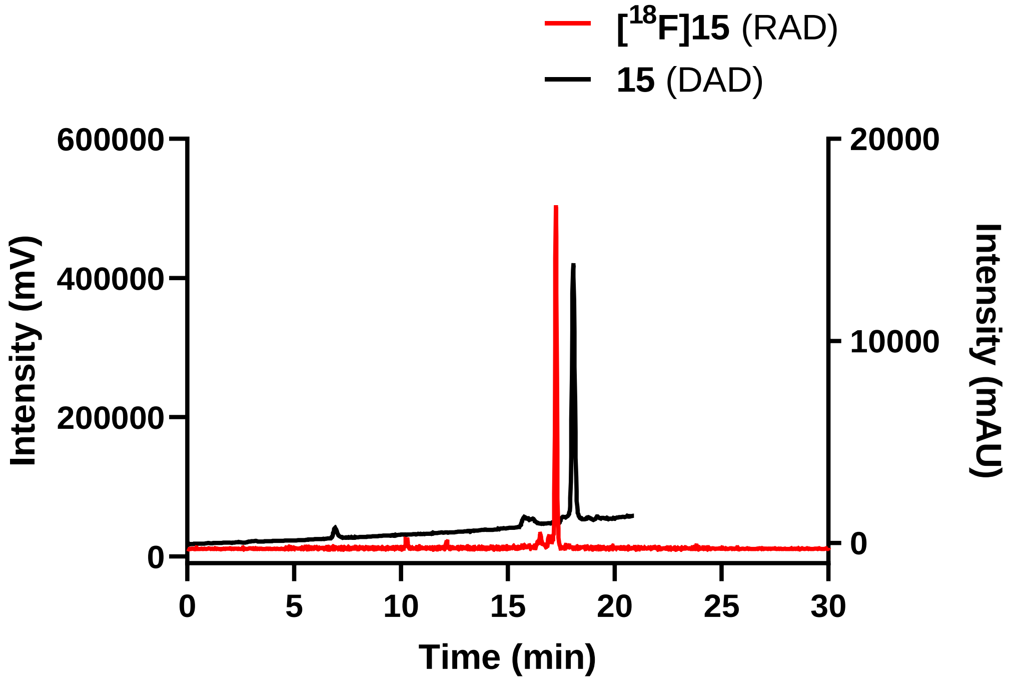

Figure S18. Radio-DAD-HPLC chromatogram of [^{18}F]AmBF$_3$-PEG$_7$-TOC (**[^{18}F]15**). Retention time of **15** t_R = 17.3 min (mV).

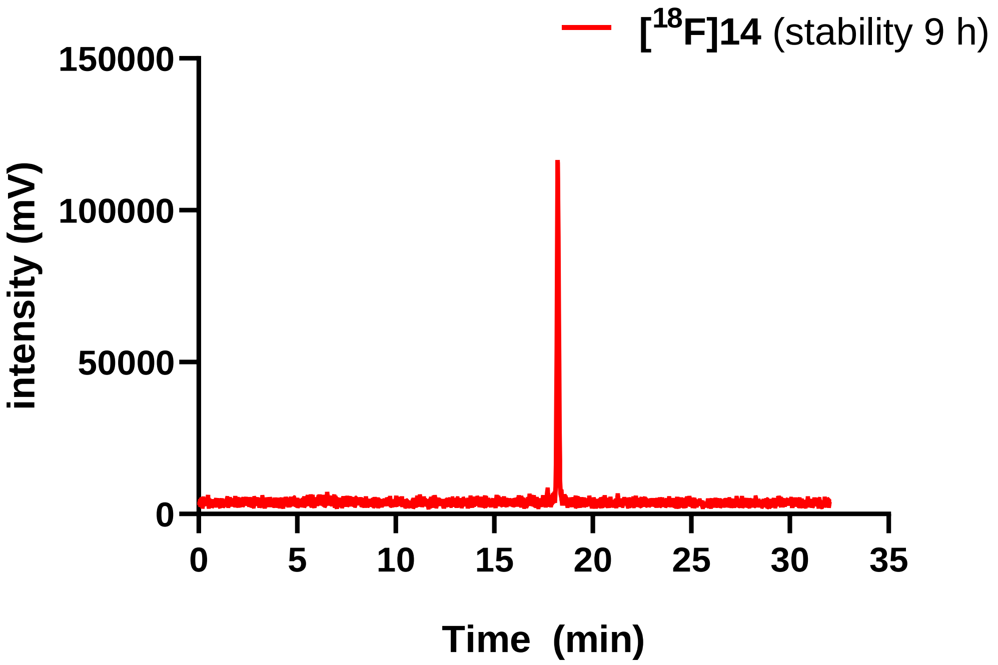

Figure S19. Hydrolytic stability of [^{18}F]AmBF$_3$-PEG$_4$-TOC (**[^{18}F]14**) at 9 hours in injection formulation.

Injection formulation of **[^{18}F]14** comprised of 4% ethanol in 0.01 M phosphate-buffered saline with a retention time of t_R = 18.7 min

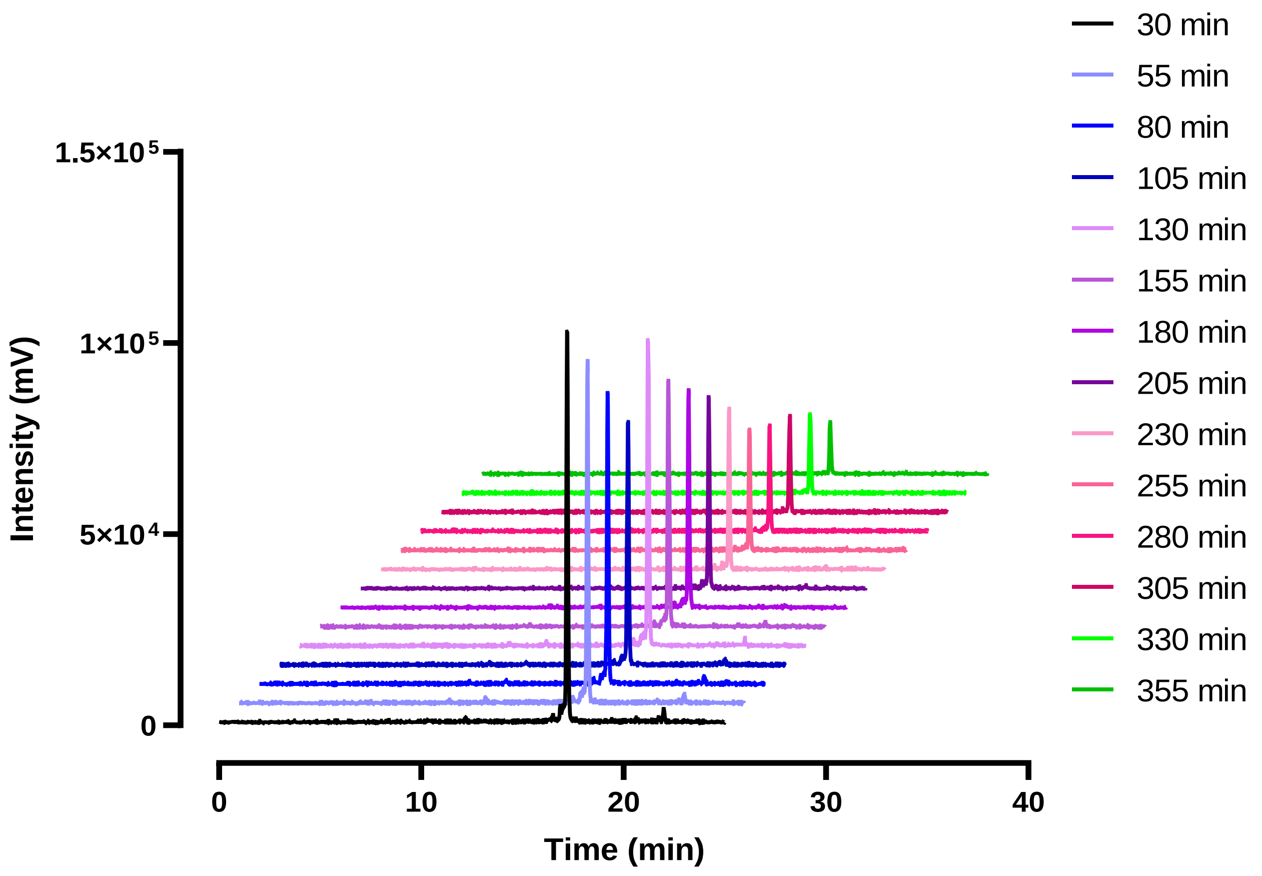

Figure S20. Hydrolytic stability of [^{18}F]AmBF$_3$-PEG$_7$-TOC (**[^{18}F]15**) up to ~6 hours in injection formulation.

Injection formulation of **[^{18}F]15** comprised of 4% ethanol in 0.01 M phosphate-buffered saline.

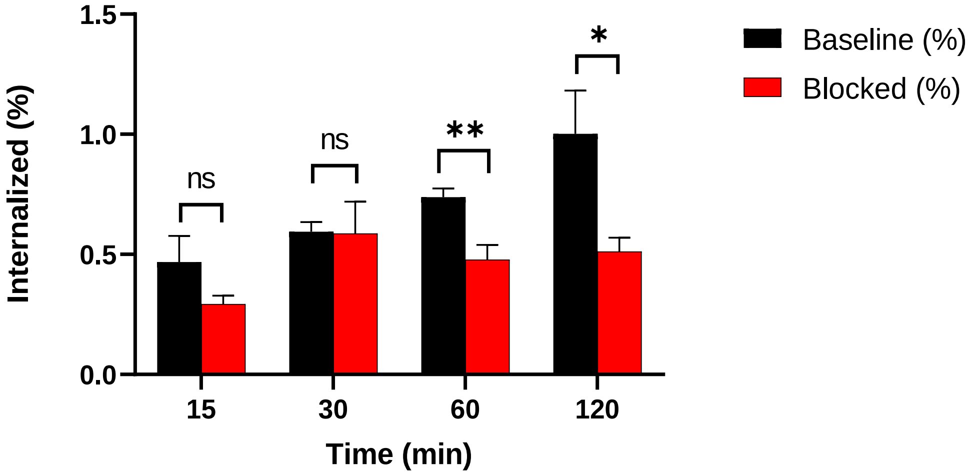

Figure S21. Cell-uptake of [^{18}F]AmBF$_3$-PEG$_4$-TOC (**[^{18}F]14**) in AR42J cells.

Cell-uptake (% internalized) of **[^{18}F]14** demonstrating specific uptake of the radiotracer in AR42J tumor cells. (*p < 0.05, **p ≤ 0.01, ns= not statistically significant, n = 3). The datapoints are presented as mean ± standard deviation.

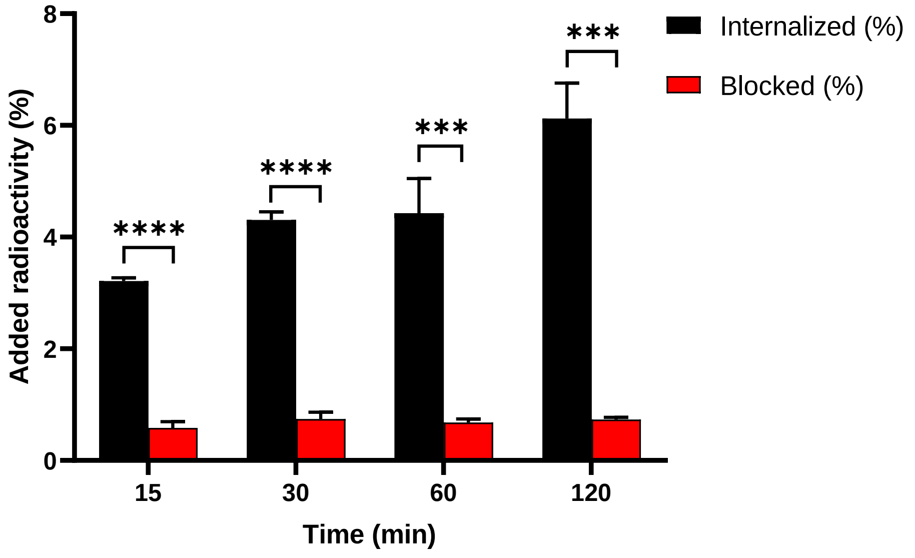

Figure S22. Cell-uptake of [^{18}F]AmBF$_3$-PEG$_7$-TOC (**[^{18}F]15**) in AR42J cells.

Cell-uptake (% internalized) of **[^{18}F]15** demonstrating specific uptake of the radiotracer in AR42J tumor cells (*** $p < 0.001$, **** $p \leq 0.0001$, $n = 3$). The datapoints are presented as mean ± standard deviation.

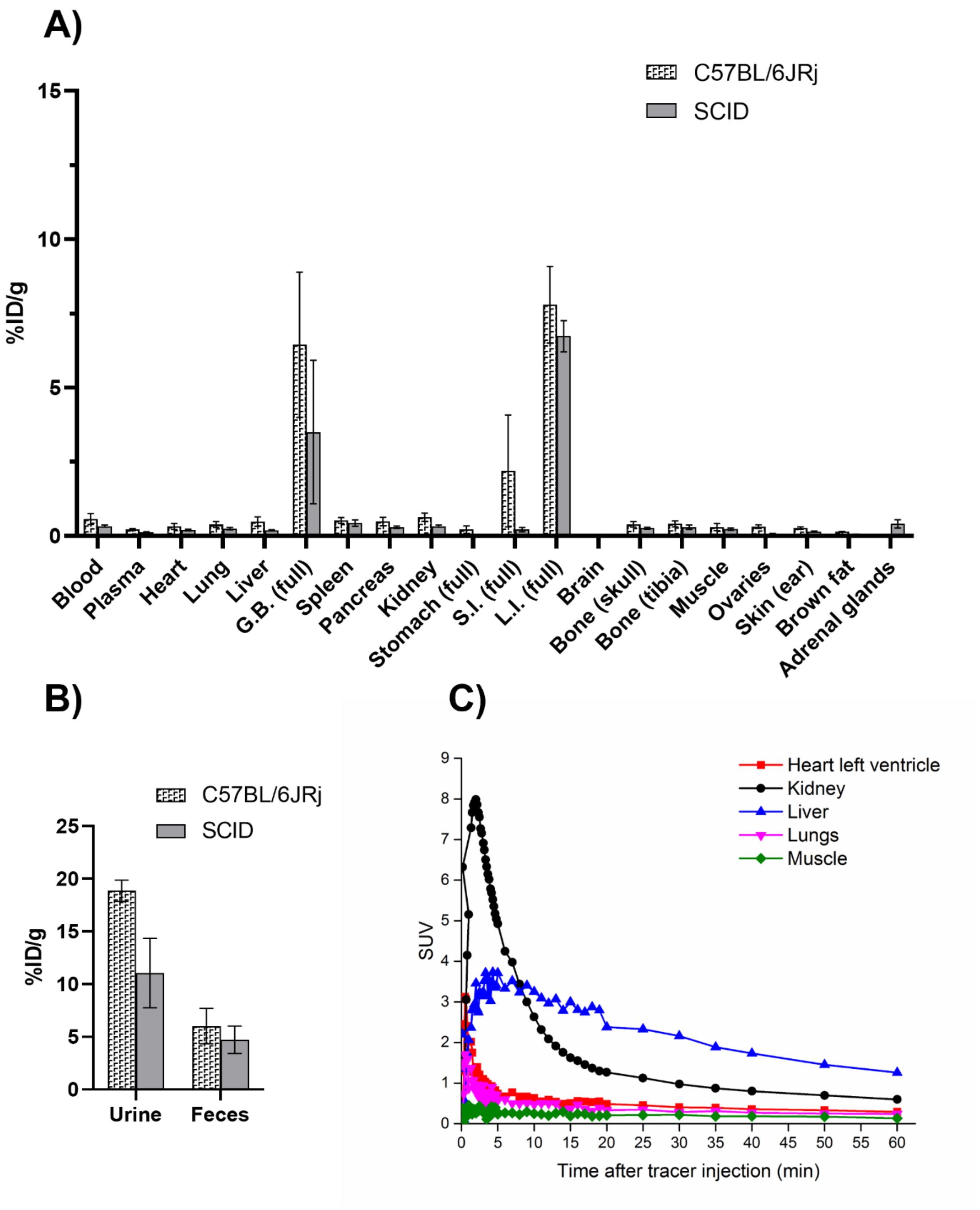

Figure S23. *Ex vivo* biodistribution (**A**, **B**) and elimination during PET/CT imaging (**C**) in selected organs and tissue samples of [^{18}F]AmBF$_3$-Tz (**[^{18}F]6**).

Ex vivo in healthy C57BL/6JRj (control) and SCID mice (control) at t = 270 minutes after intravenous administration of **[^{18}F]6** in healthy SCID mice (control) after intravenous administration (**A**: biodistribution, **B**: elimination to urine and feces from *ex vivo* biodistribution samples **C**: SUV in selected organs during PET/CT acquisition,). G.B. = gallbladder; S.I. = small intestine; LI. = large intestine. TACs from PET/CT imaging presented as SUVs. The datapoints are presented as mean ± standard deviation (n = 4).

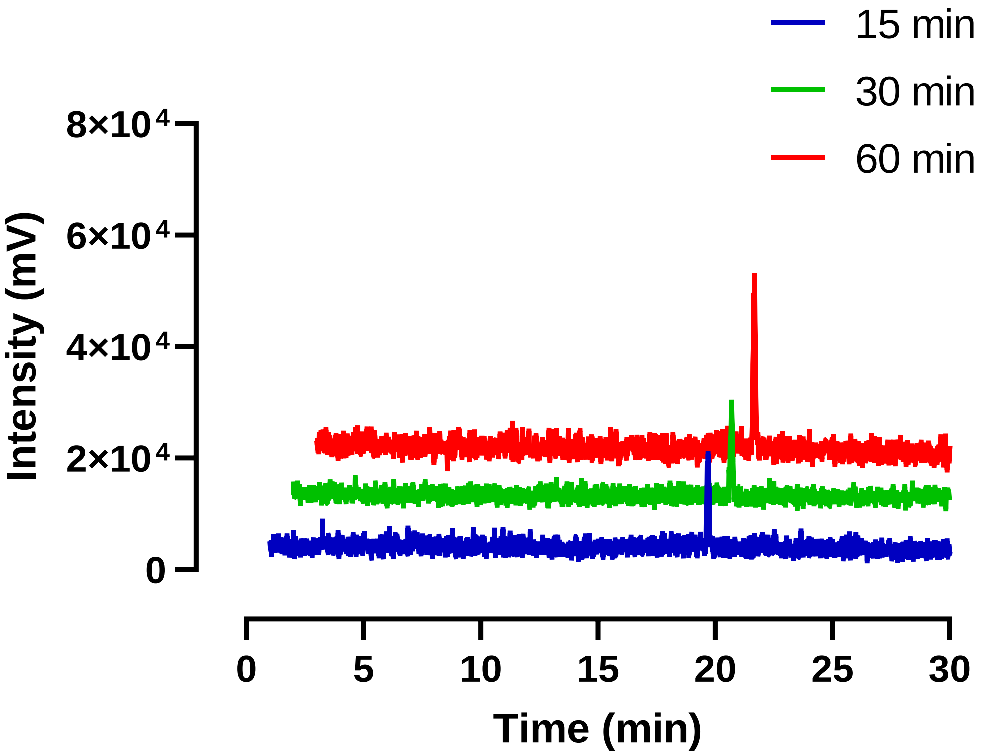

Figure S24. *Ex vivo* stability of [^{18}F]AmBF$_3$-PEG$_4$-TOC (**[^{18}F]14**) in AR42J tumor bearing mouse blood (Radio-HPLC).

HPLC chromatogram of a sample extracted from mouse blood (*ex vivo*). The radiopeak corresponding to intact **[^{18}F]14** was detected at a retention time of t_R = 18.6 min.

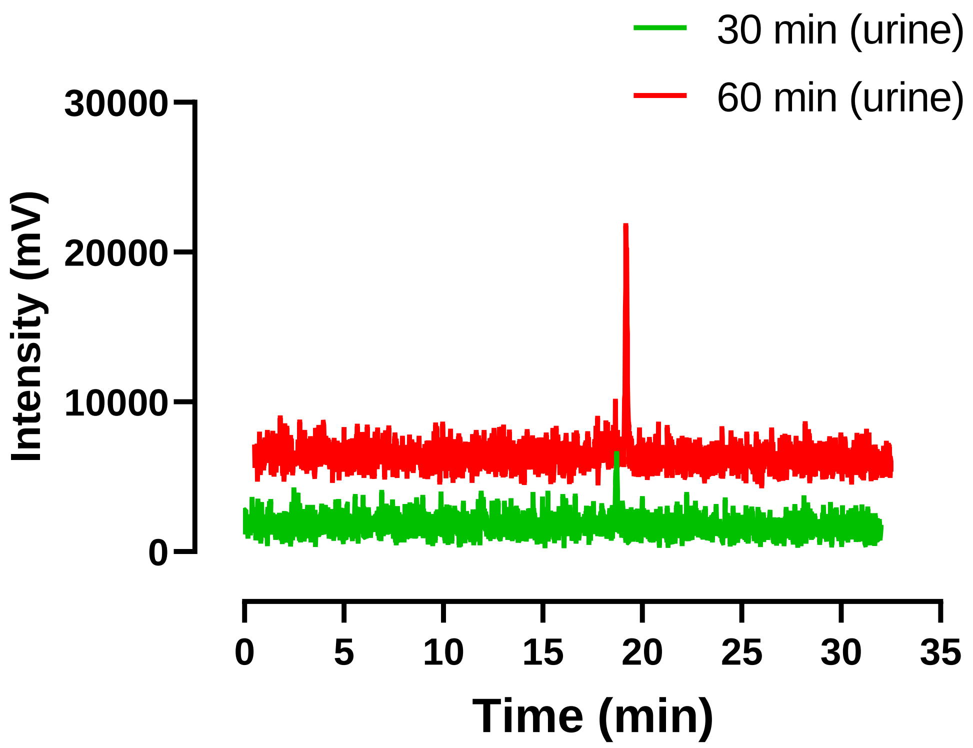

Figure S25. *Ex vivo* stability of [^{18}F]AmBF$_3$-PEG$_4$-TOC (**[^{18}F]14**) in urine (Radio-HPLC).

HPLC chromatogram of the tracer extracted from mouse urine (*ex vivo*). The radiopeak corresponding to intact **[^{18}F]14** was detected at a retention time of t_R = 18.6 min.

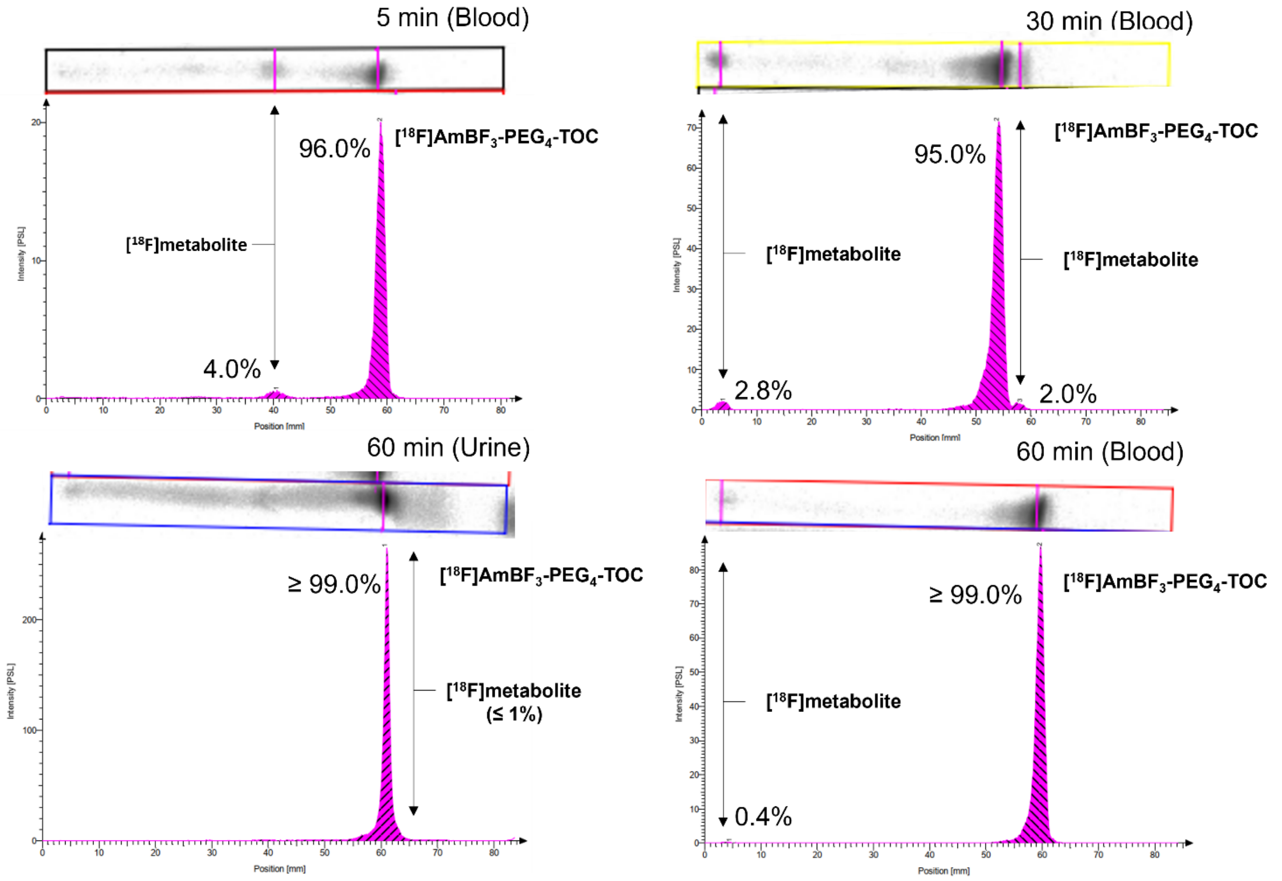

Figure S26. *Ex vivo* stability of [^{18}F]AmBF$_3$-PEG$_4$-TOC (**[^{18}F]14**) in AR42J tumor bearing mouse blood 5 and 30 minutes post-injection (Radio-TLC).

Radio-TLC revealed a slightly polar metabolite (4%) in blood 5 minutes post-injection with a retardation factor (R_f) of R_f = 40 mm. At 30 minutes post-injection the radiometabolite at 40 mm had disappeared and instead a highly polar metabolite (2.8%) at 4.0 mm was visible, and was accompanied by another metabolite (2.0%) eluting in the immediate vicinity of **[^{18}F]14** at R_f = 58 mm. At 60 minutes post-injection the tracer accounted for ≥99% of the radioactivity both in blood and urine (*ex vivo*).

Table S1. *Ex vivo* blood component distribution analysis from mouse blood after intravenous administration of **[^{18}F]14**.

	**Red blood cells**		**Protein bound**		**Free fraction**	
	Average (%)	Stdv.(%)	Average (%)	Stdv.(%)	Average (%)	Stdv.(%)
**5 min**	21.6	4.4	5.5	0.9	72.9	5.1
**60 min**	24.5	5.4	7.00	1.5	68.5	5.3
**60 min (B)**	29.7	2.9	14.6	1.4	55.7	11.4

B=blocked with octreotide, (*n* = 2).

Table S2. Biodistribution and statistical analysis of **[18F]14** and **[18F]15** at 60 minutes after i. v. administration expressed as %ID/g.

	**[18F]14 Baseline (%ID/g)**	**[18F]14 Blocked (%ID/g)**	**Statistical significance (*p*-value)**	**[18F]15 Baseline (%ID/g)**	**[18F]15 Blocked (%ID/g)**	**Statistical significance (*p*-value)**
**Urine**	84.9 ± 58.6	41.9 ± 15.7	* (0.0449)	174.2 ± 73.4	303.4 ± 145.8	ns (0.2654)
**Blood**	10.2 ± 2.3	7.4 ± 1.5	** (0.0079)	9.6 ± 3.3	7.2 ± 2.5	ns (0.2918)
**Spleen**	1.2 ± 0.5	0.9 ± 0.1	* (0.0473)	1.4 ± 0.3	0.9 ± 0.2	* (0.0375)
**Pancreas**	1.1 ± 0.2	0.8 ± 0.2	** (0.0087)	1.6 ± 0.5	0.9 ± 0.5	ns (0.1655)
**Kidney**	14.3 ± 4.5	11.4 ± 3.7	ns (0.1806)	21.0 ± 4.5	15.9 ± 2.7	ns (0.0919)
**Gallbladder**	17.8 ± 5.2	10.7 ± 6.3	ns (0.0530)	9.1 ± 7.0	18.8 ± 10.3	ns (0.3923)
**Liver**	30.0 ± 9.4	26.2 ± 15.8	ns (0.6075)	19.0 ± 5.4	11.8 ± 4.7	ns (0.1032)
**Lung**	6.2 ± 2.4	5.5 ± 2.7	ns (0.5991)	5.4 ± 2.3	3.6 ± 1.4	ns (0.1948)
**Heart**	2.4 ± 0.7	1.9 ± 0.4	ns (0.0565)	2.6 ± 0.6	2.1 ± 0.5	ns (0.2824)
**Muscle**	0.6 ± 0.2	0.5 ± 0.1	* (0.0404)	1.1 ± 0.9	0.5 ± 0.1	ns (0.2078)
**Bone (t.)**	1.3 ± 0.6	1.0 ± 0.6	ns (0.2563)	1.1 ± 0.4	0.8 ± 0.2	ns (0. 1095)
**Bone (occ.)**	1.3 ± 0.6	1.1 ± 0.5	ns (0.5751)	0.9 ± 0.3	1.0 ± 0.3	ns (0.7031)
**Brain**	0.3 ± 0.1	0.3 ± 0.1	ns (0.3096)	0.3 ± 0.1	0.2 ± 0.1	ns (0.0655)
**Stomach (+cont.)**	0.6 ± 0.2	0.6 ± 0.2	ns (0.7522)	1.4 ± 0.8	0.6 ± 0.1	ns (0.1197)
**S. I. (+cont.)**	2.6 ± 0.8	1.9 ± 0.4	* (0.0266)	3.6 ± 2.1	5.0 ± 1.7	ns (0.3371)
**L. I. (+cont.)**	1.1 ± 1.1	0.7 ± 0.3	ns (0.2071)	1.0 ± 0.7	0.7 ± 0.3	ns (0.3750)
**Skin**	2.1 ± 0.9	1.6 ± 0.4	ns (0.1348)	2.4 ± 0.9	1.6 ± 0.2	ns (0.1041)
**Tumor**	3.1 ± 0.7	2.2 ± 0.6	* (0.0120)	4.5 ± 1.0	3.1 ± 0.5	* (0.0143)

ns = not significant, **p*<0.05, ***p*<0.01, and ****p*<0.001. Differenced between groups were considered significant when $p \leq 0.05$.

t.; tibia, occ.; occipital, cont.; content, S.I.; small intestines, L.I.; large intestine. Unpaired *t*-test with Welch´s correction was used to calculate statistical significances (significant *p*<0.05). The datapoints are presented as mean ± standard deviation.

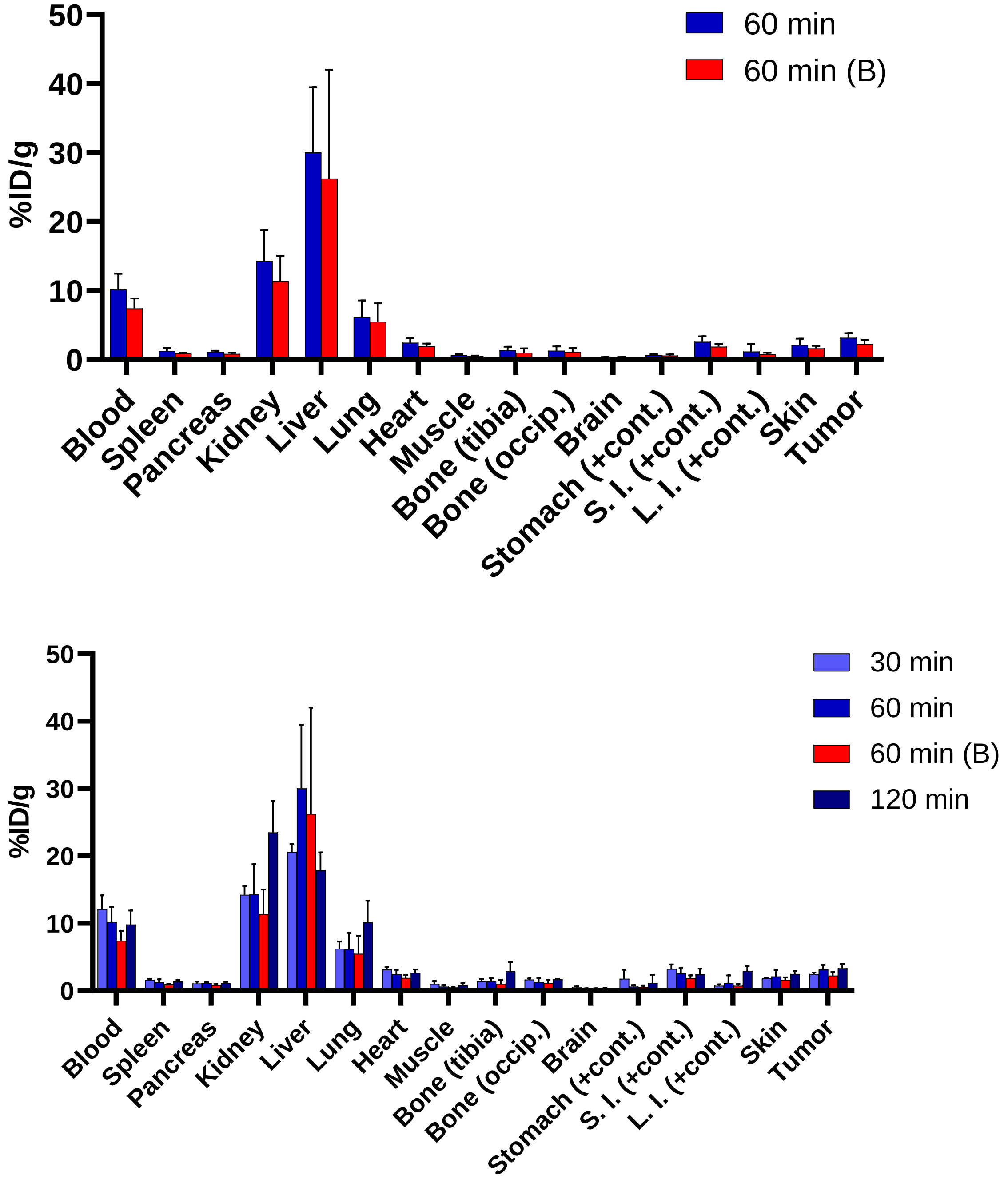

Figure S27. *Ex vivo* biodistribution of [^{18}F]AmBF$_3$-PEG$_4$-TOC (**[^{18}F]14**) (*ex vivo*; 60 minutes post-injection and 30, 60 and 120 min post-injection).

Biodistribution of radioactivity after intravenous administration of [^{18}F]AmBF$_3$-PEG$_4$-TOC (**[^{18}F]14**) in AR42J tumor bearing Rj:NMRI-*Foxn1*$^{nu/nu}$ mice. (*$p < 0.05$). S.I. = small intestine; L.I. = large intestine, occip. = occipital, cont. = contents, B = blocked. The datapoints are presented as mean ± standard deviation.

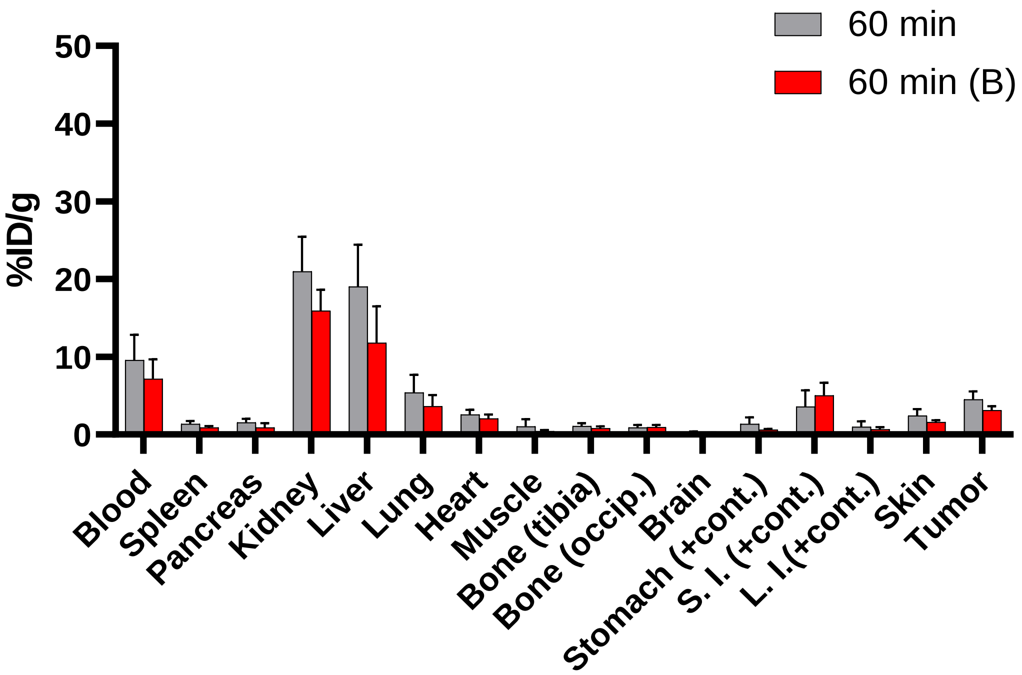

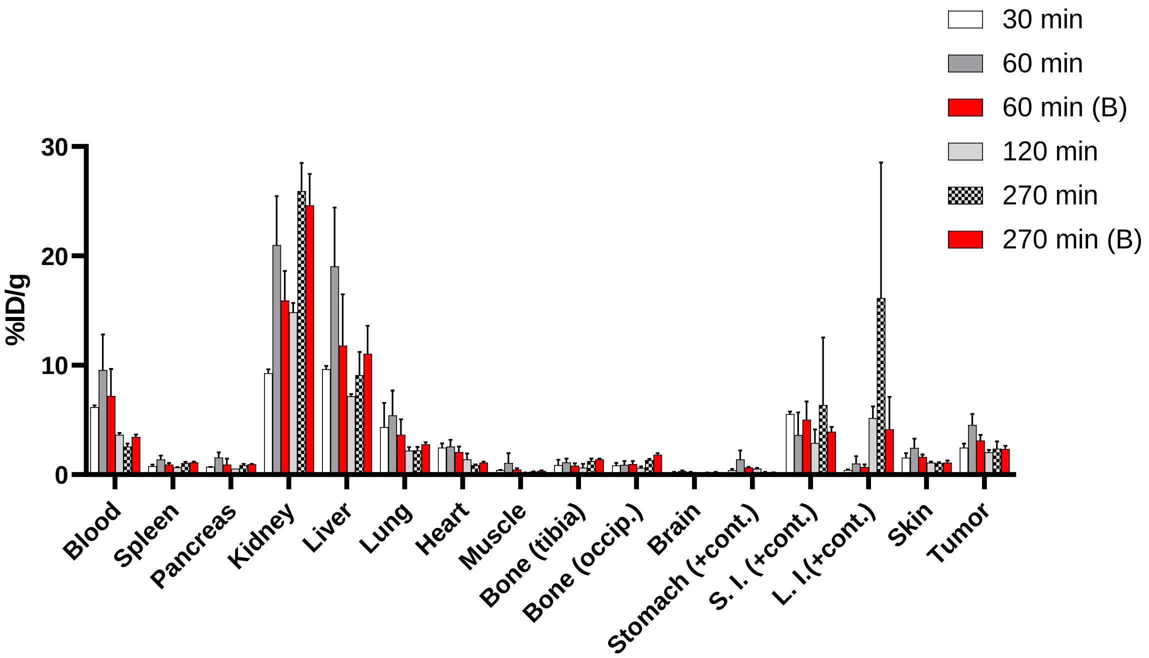

Figure S28. *Ex vivo* biodistribution of [^{18}F]AmBF$_3$-PEG$_7$-TOC (**[^{18}F]15**) (*ex vivo*; at 30 min, 60 min and 120 min post-injection, *ex vivo* after PET/CT; 270 min). Biodistribution of radioactivity in AR42J rat pancreatic tumor bearing Rj:NMRI-Foxn1$^{nu/nu}$ mice at t = 60 min after intravenous administration. The uptake was blocked using a dose (44 nmol) of octreotide that was co-administered with the radiotracer, and the mice were euthanized at selected time points. (*p < 0.05). S.I. = small intestine; L.I. = large intestine, cont. = contents, B = blocked. The datapoints are presented as mean ± standard deviation.

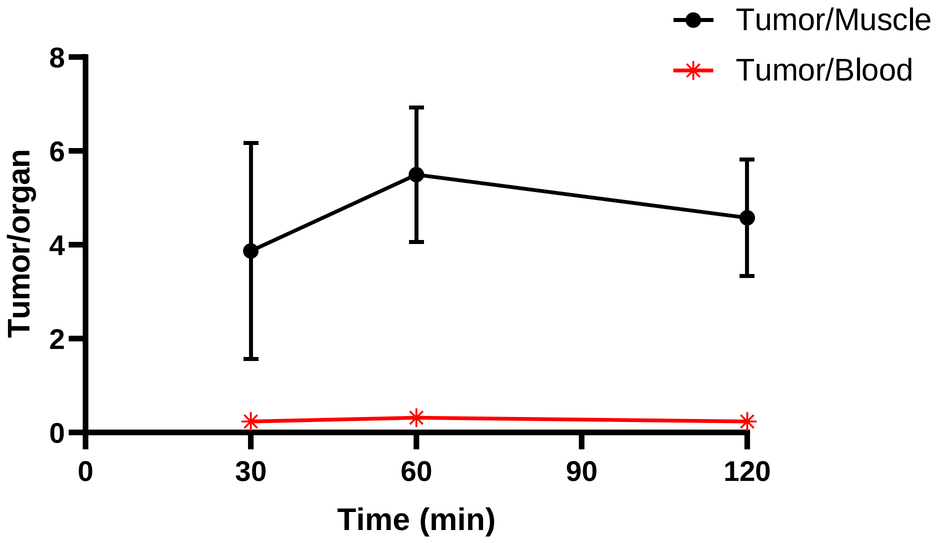

Figure S29. Tumor-to-muscle (T/M) and tumor-to-blood (T/B) ratios of **[^{18}F]14** after *ex vivo* biodistribution (*n* ≥ 4).

The datapoints are presented as mean ± standard deviation.

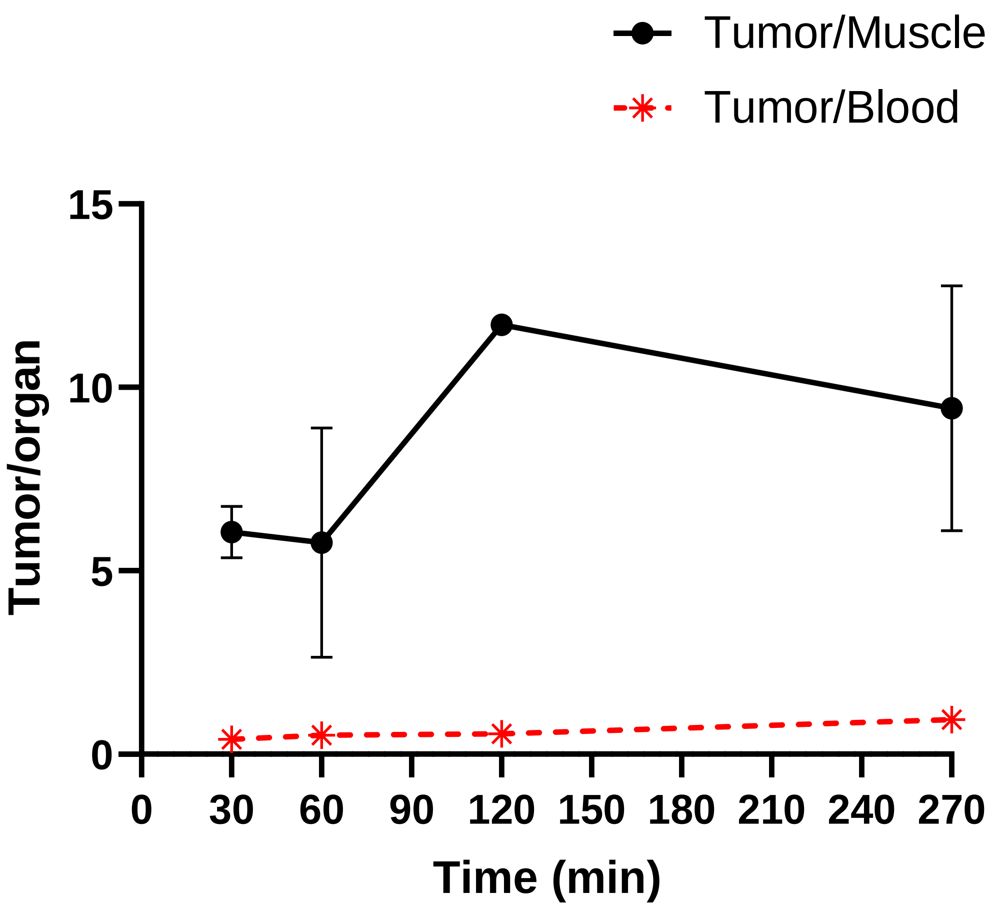

Figure S30. Tumor-to-muscle (T/M) and tumor-to-blood (T/B) ratios of [^{18}F]AmBF$_3$-PEG$_7$-TOC (**[^{18}F]15**) after *ex vivo* biodistribution (*n* ≥ 2).

The datapoints are presented as mean ± standard deviation.

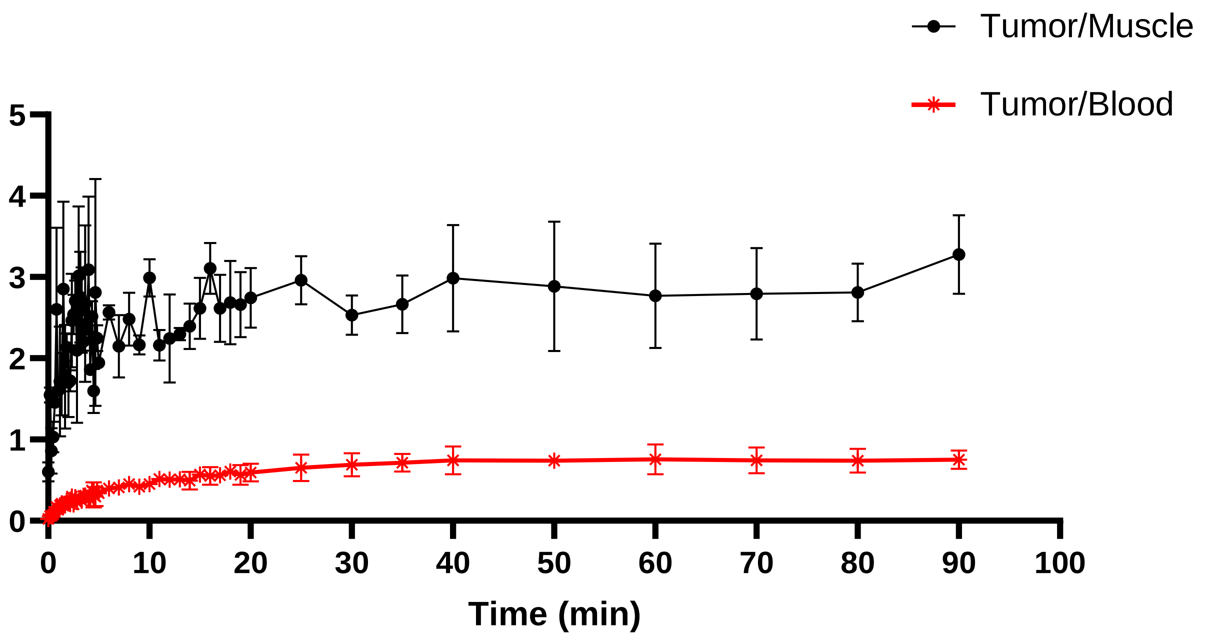

Figure S31. Tumor-to-muscle (T/M) and tumor-to-blood (T/B) ratios of [^{18}F]AmBF$_3$-PEG$_7$-TOC (**[^{18}F]15)** during PET/CT imaging.

Blood radioactivity was determined by drawing a region-of-interest (ROI) for left ventricle of the heart (n = 2). The datapoints are presented as mean ± standard deviation.

Table S3. Dosimetry calculations.

Results after intravenous administration of **[^{18}F]15** from OLINDA/EXM (Version 2.1, Vanderbilt University, 2012), where ICRP 89 reference adult male (73 kg) and ICRP 103 radiation weighting factors were used. Absorbed doses to each target organ is given in units mGy/MBq and the effective dose in units mSv/MBq. The difference in radiation burden based on baseline (non-block) and blocked mice is negligible.

**Target organ**	**Baseline Avg. (mGy/MBq)**	**Baseline Std. (mGy/MBq)**	**Blocked Avg. (mGy/MBq)**	**Blocked Std. (mGy/MBq)**
Adrenals	0.0190	0.0008	0.0185	0.0001
Brain	0.0102	0.0003	0.0103	0.0000
Esophagus	0.0129	0.0004	0.0129	0.0002
Eyes	0.0102	0.0003	0.0103	0.0000
Gallbladder Wall	0.0194	0.0011	0.0190	0.0011
Left colon	0.0143	0.0001	0.0144	0.0001
Small Intestine	0.0144	0.0001	0.0145	0.0000
Stomach Wall	0.0141	0.0002	0.0141	0.0001
Right colon	0.0151	0.0001	0.0150	0.0001
Rectum	0.0136	0.0003	0.0137	0.0000
Heart Wall	0.0148	0.0008	0.0150	0.0010
Kidneys	0.0366	0.0016	0.0337	0.0043
Liver	0.0334	0.0050	0.0313	0.0040
Lungs	0.0109	0.0030	0.0106	0.0030
Pancreas	0.0156	0.0002	0.0154	0.0001
Prostate	0.0135	0.0003	0.0136	0.0000
Salivary Glands	0.0118	0.0002	0.0119	0.0000
Red Marrow	0.0113	0.0000	0.0114	0.0001
Osteogenic Cells	0.0120	0.0001	0.0121	0.0001
Spleen	0.0137	0.0001	0.0136	0.0001
Testes	0.0112	0.0003	0.0113	0.0000
Thymus	0.0122	0.0002	0.0122	0.0003
Thyroid	0.0118	0.0000	0.0119	0.0003
Urinary Bladder Wall	0.0134	0.0003	0.0135	0.0000
Total Body	0.0121	0.0001	0.0121	0.0000
**Effective Dose (mSv/MBq)**	**0.0112**	**6.36E-04**	**0.011**	**2.83E-04**

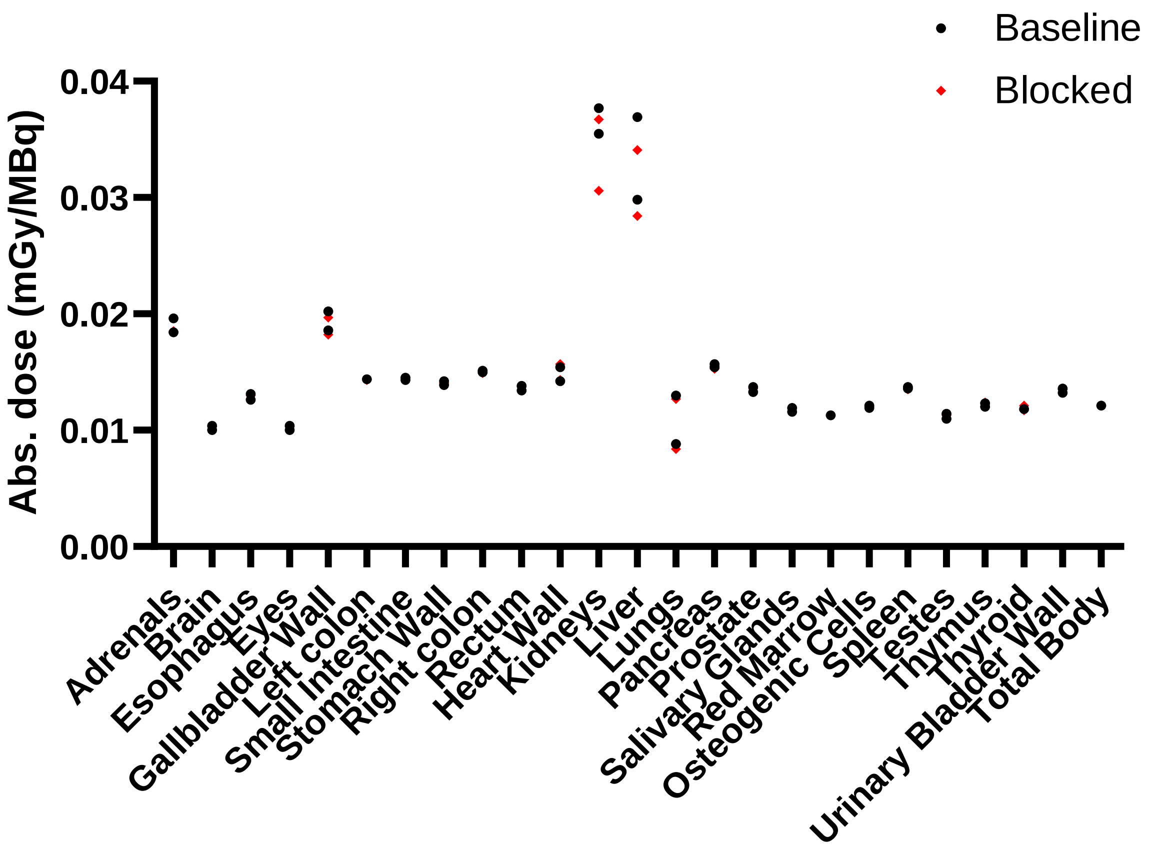

Figure S32. Absorbed doses (mGy/MBq) in selected tissues in AR42J tumor-bearing mice at baseline and blocking conditions (co-injection with 45 µg, 44 nmol octreotide) after intravenous administration of **[^{18}F]15** demonstrating highest absorbed doses to kidneys and liver. Individual values plotted as a single datapoint.

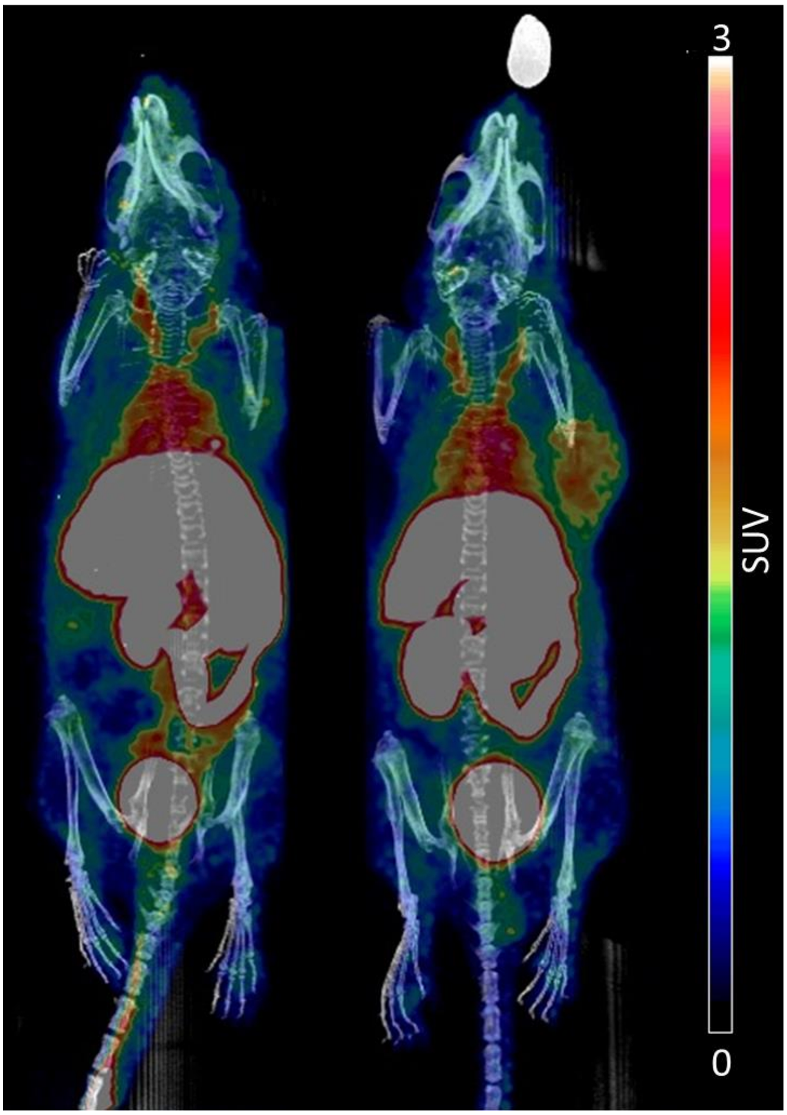

Figure S33. Maximum intensity projections of PET/CT at 20–80 min post-injection of [^{18}F]AmBF$_3$-PEG$_7$-TOC (**[^{18}F]15**) in AR42J tumor bearing mice. Blocked (co-injection with 45 µg, 44 nmol octreotide) on the left.

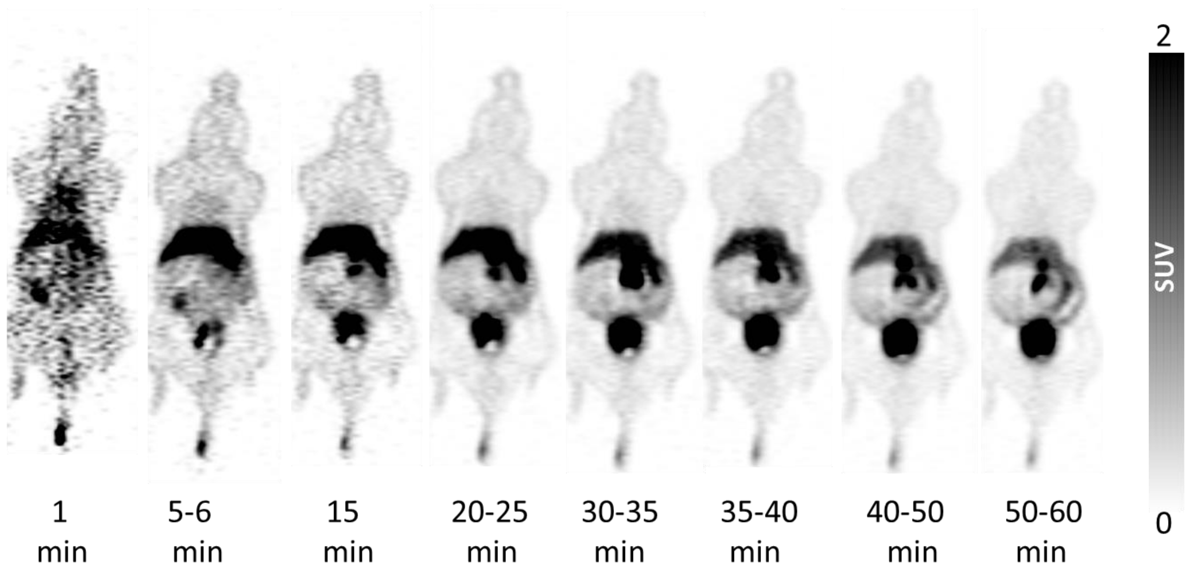

Figure S34. Standalone single-slice blank-on-white PET images of **[^{18}F]6** in a male SCID mouse.

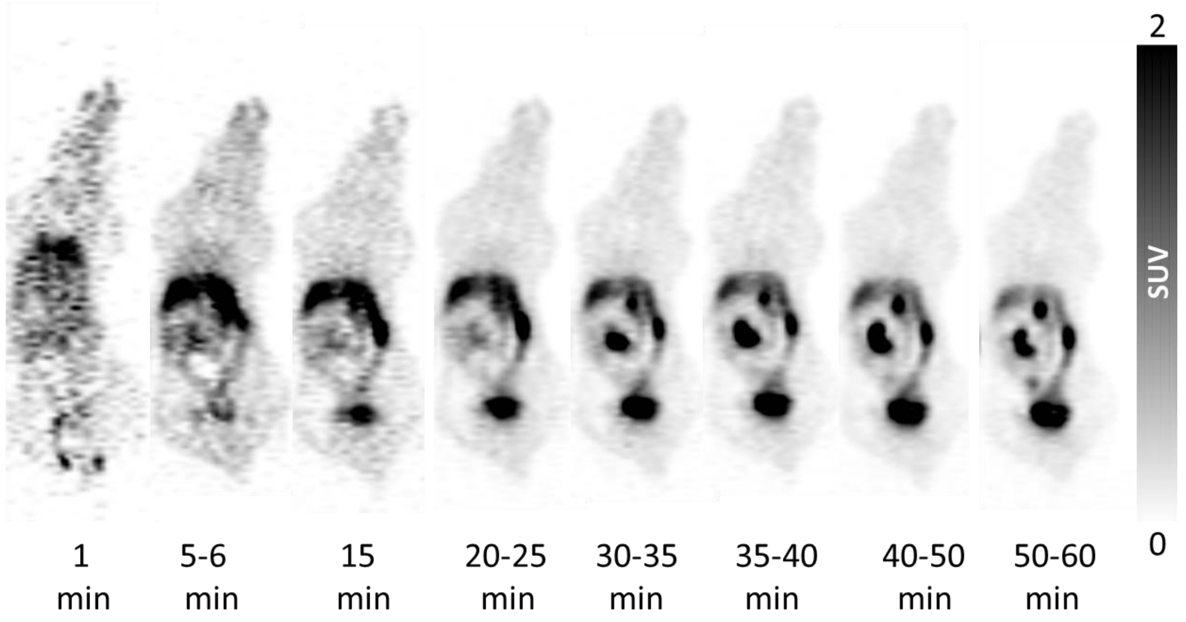

Figure S35. Standalone single-slice blank-on-white PET images of **[^{18}F]6** in a healthy female C57BL/6JRj mouse.

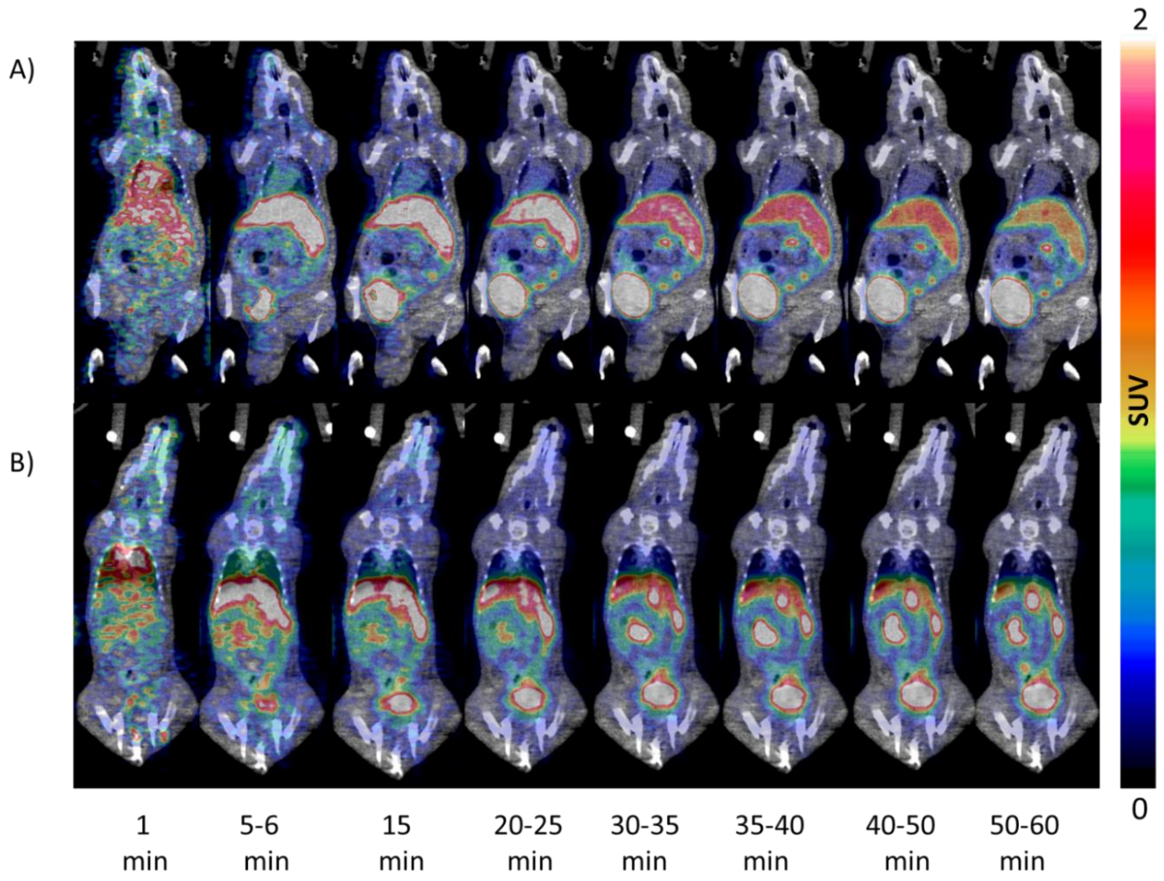

Figure S36. Single-slice coronal PET/CT images of **[^{18}F]6** in (**A**) male SCID and (**B**) healthy female C57BL/6JRj mice.

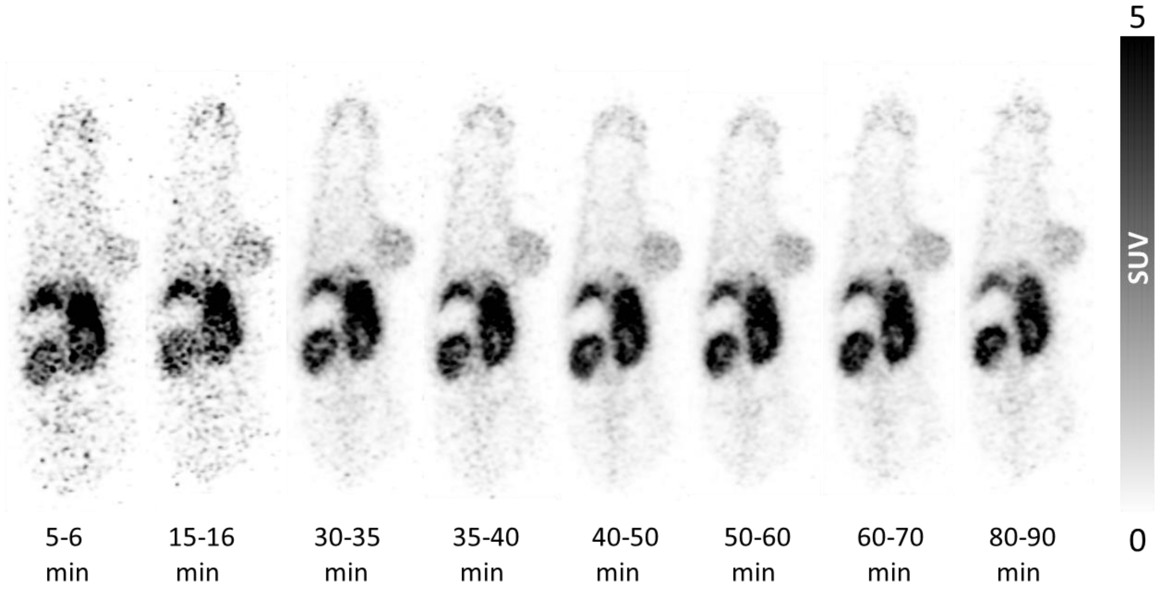

Figure S37. Standalone single-slice blank-on-white PET images of **[^{18}F]15** in baseline conditions studied in a Rj:NMRI-Foxn1$^{nu/nu}$ mouse bearing subcutaneous rat pancreatic AR42J xenografts.

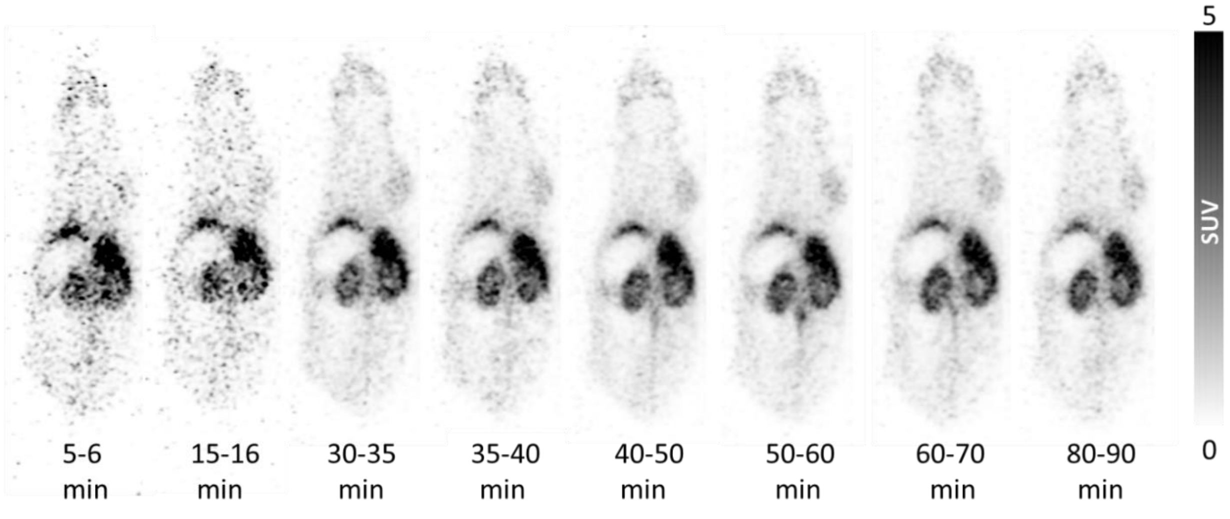

Figure S38. Standalone single-slice blank-on-white PET images of **[^{18}F]15** in blocking conditions studied in aRj:NMRI-Foxn1$^{nu/nu}$ mouse bearing subcutaneous rat pancreatic AR42J xenografts.

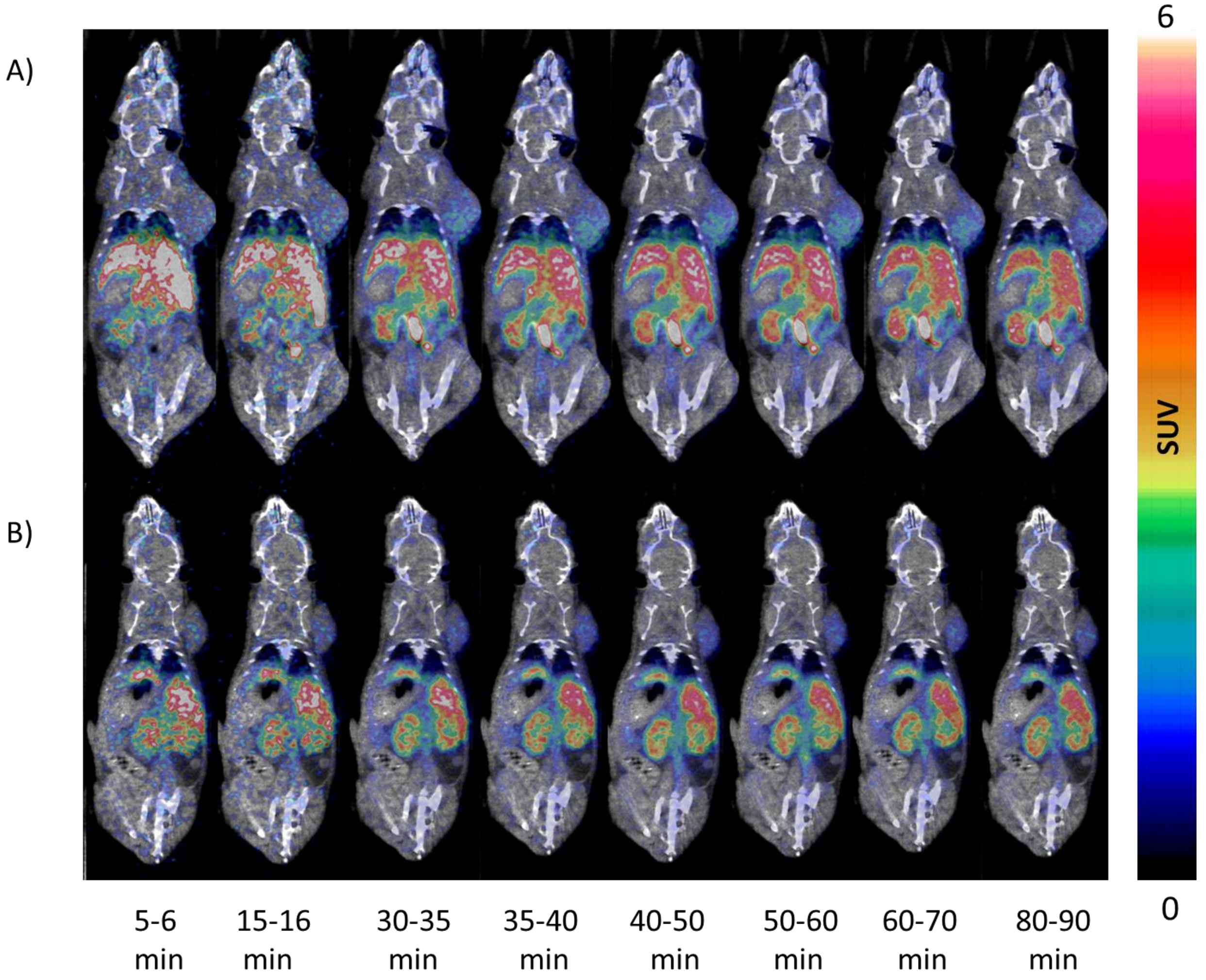

Figure S39. Single-slice coronal PET/CT images of **[^{18}F]15** in baseline (**A**) and blocking (**B**) conditions studied in Rj:NMRI-Foxn1$^{nu/nu}$ mice bearing subcutaneous rat pancreatic AR42J xenografts.
